# Determinants of Exposure Therapy Implementation in Clinical Practice for the Treatment of Anxiety, OCD, and PTSD: A Systematic Review

**DOI:** 10.1007/s10567-024-00478-3

**Published:** 2024-04-17

**Authors:** J. I. Racz, A. Bialocerkowski, I. Calteaux, L. J. Farrell

**Affiliations:** 1 School of Applied Psychology, Griffith University, Southport, QLD Australia; 2 Griffith Health, Griffith University, Southport, QLD Australia

**Keywords:** Exposure therapy, Implementation, Anxiety, Obsessive-compulsive disorder, Post-traumatic stress disorder, Theoretical domains framework

## Abstract

Exposure therapy (ET) forms a vital part of effective psychotherapy for anxiety-related presentations including anxiety disorders, obsessive-compulsive disorder (OCD), and post-traumatic stress disorder (PTSD), and is often underutilised in clinical practice. Using the Theoretical Domains Framework (TDF), this systematic review synthesised existing literature on the determinants of ET implementation for anxiety-related presentations and examined differences across presentations and developmental subgroups. Fifty-two eligible studies were assessed using the Mixed Methods Appraisal Tool, with 389 results (99%) mapped onto the TDF. Results suggested that clinicians’ negative beliefs about the consequences of ET were commonly associated with reduced implementation. It also appeared that whilst broad unspecified ET training may be related to improved implementation for anxiety disorders; greater implementation for complex presentations (i.e., PTSD) likely requires more specialised training involving practical components. A subset of domains (e.g., social/professional role and identity) accounted for most results, whilst some remain unexplored (i.e., optimism; reinforcement; memory, attention, and decision processes) or underexplored (i.e., behavioural regulation). Likewise, specific presentations and developmental subgroups (i.e., PTSD and adults) represented a greater proportion of results in the literature than others (i.e., OCD and youth). Future research exploring ET implementation, across specific presentations and developmental subgroups, would benefit from integrating implementation science frameworks to guide the development of targeted, comprehensive strategies to close the research-practice gap of ET for the treatment of anxiety-related presentations.

**Supplementary Information:**

The online version contains supplementary material available at 10.1007/s10567-024-00478-3.

Anxiety disorders (i.e., separation anxiety disorder, selective mutism, specific phobia, social anxiety disorder, panic disorder, agoraphobia, generalised anxiety disorder) are characterised by excessive anxiety, fear, and avoidance, often onset in childhood, and persist if untreated (American Psychiatric Association, 2022). Obsessive-compulsive disorder (OCD) and post-traumatic stress disorder (PTSD), previously defined as anxiety disorders in earlier taxonomic tools (e.g., American Psychiatric Association, 2000), are characterised by similar underlying mechanisms to anxiety disorders and share similar patterns of symptom expression. For instance, maladaptive threat appraisals and dysfunctional belief biases (e.g., intolerance of uncertainty) are common and accompanied by a fear response, avoidance, and other safety seeking behaviours (Farrell et al., 2019). These disorders are common with a combined 12-month prevalence of 17%, and disorder specific figures ranging from 4% for OCD, 6% for PTSD, and up to 7% for some anxiety disorders (Australian Bureau of Statistics, 2023). These anxiety-related presentations (i.e., anxiety disorders, OCD, and PTSD) commonly co-occur and are highly comorbid with mood disorders, resulting in a significant burden for clients (Bandelow & Michaelis, 2015). Alongside impairing symptoms, the long-term consequences of these disorders include educational underachievement in youth (Woodward & Fergusson, 2001), reduced quality of life, and increased suicidality (Angst et al., 2004; McFarlane, 2000; Wittchen & Hoyer, 2001), highlighting the critical importance of effective and early intervention.

Empirically supported treatment is effective for anxiety-related presentations, namely cognitive-behavioural therapy (CBT), of which exposure therapy (ET) is arguably the most important therapeutic component (Carpenter et al., 2018; Muris, 2019; Whiteside et al., 2020). Yet ET is widely underutilised in community mental health settings (e.g., Becker-Haimes et al., 2017). Whilst there have been several barriers to the implementation of ET explored in the literature (e.g., clinician attitudes; Blakey et al., 2018), to date there has not been a theoretically driven approach to synthesising the existing literature within an implementation science framework. Moreover, little is known about the determinants of implementation across different client groups, such as disorder presentations or development subgroups. Using implementation science to identify, prioritise, and synthesise determinants of ET use in practice will likely drive the development of targeted, integrated, and multi-level interventions to increase ET utilisation in practice. This systematic review aims to synthesise the existing evidence on the determinants of ET implementation in treating anxiety-related presentations, using the Theoretical Domains Framework (TDF; Cane et al., 2012; Michie et al., 2005), to identify gaps in knowledge, guide future research, and inform future implementation strategies.

## Evidence-Based Treatments for Anxiety-Related Presentations

Evidence-based treatments for anxiety-related presentations include pharmacotherapies (e.g., serotonin-noradrenaline reuptake inhibitors) and various psychotherapies, most notably CBT, the gold-standard psychotherapy for anxiety-related presentations (Bandelow et al., 2015). Combining cognitive and behavioural therapies, CBT modifies maladaptive cognitions and consequently addresses problematic behaviours that perpetuate emotional disturbances (Tolin, 2016). CBT for the treatment of anxiety-related presentations is a multi-component therapy, typically involving psychoeducation, anxiety management, cognitive therapy, ET, problem-solving, and relapse prevention (Tolin, 2016). Whilst limited research has examined the efficacy of individual components of CBT, ET is an exception (Creswell et al., 2020), with evidence suggesting that ET uniquely reduces symptom severity and improves functioning when accounting for relaxation training and cognitive restructuring (Peris et al., 2015). ET is consistently identified as an essential component of CBT for the treatment of anxiety disorders (e.g., Bandelow et al., 2022), OCD (e.g., National Institute for Health and Care Excellence, 2005), and PTSD (e.g., American Psychological Association, 2017) in clients from across the lifespan (Katzman et al., 2014).

ET is predicated on the CBT model conceptualising anxiety as originating in aversive situations, which elicit an adaptive threat response (Albano & Kendall, 2002). However, when generalised to safe contexts, these automatic threat cognitions become maladaptive, eliciting distress and behavioural avoidance in the absence of actual danger. Behavioural avoidance is proposed to maintain maladaptive threat appraisals and perceived coping by limiting opportunities for maladaptive appraisals to be challenged, perpetuating a cycle of behavioural avoidance (Moghaddam et al., 2015). ET targets this cycle using therapist-guided, within and between session, systematic, gradual, and repeated exposure to aversive stimuli, intended to disconfirm maladaptive threat cognitions, violate threat expectancies, and facilitate habituation of the feared response (i.e., steady decline in arousal and fear over time; Abramowitz et al., 2019). ET has been adapted from this common premise into distinct variations for different presentations, such as exposure and response prevention (ERP) for OCD, which involves refraining from engaging in neutralising responses during ET (Hezel & Simpson, 2019). ERP aims to break the cycle of OCD distress and avoidance by reducing compulsive rituals with the goal of assisting clients to learn new (non-threatening) information about feared stimuli and to develop distress tolerance (Foa et al., 2012). ET has also been adapted into prolonged exposure for PTSD, entailing imaginal and in vivo exposure delivered over prolonged periods with the goal of processing trauma responses (Foa, 2011). Specifically, by exposing clients to the traumatic memory, prolonged exposure aims to allow them to challenge maladaptive beliefs they may have been formed around the trauma, about the world and themselves (Foa, 2011). In addition to the different forms ET can take for various presentations, it can also be delivered via different modalities. For example, it may be delivered in vivo (i.e., exposure to actual stimuli), as imaginal exposure, via virtual reality, or interoceptively (i.e., eliciting aversive physical sensations). These variations mean that, as a therapeutic technique, ET is flexibly delivered, requiring clinicians to understand how to implement it appropriately and effectively for the needs of the client and their presentation (Abramowitz et al., 2019). Amongst anxiety-related presentations, ET’s status as the gold-standard treatment is supported by several meta-analyses. For example, a meta-analysis of results from 10 randomised placebo-controlled trials found that the use of ET resulted in a large reduction in symptom outcomes relative to placebo treatment in clients with anxiety disorders, OCD, and PTSD in adults (Carpenter et al., 2018). Similar findings have been reported amongst youth, with meta-analyses reporting larger effects for ET relative to pharmacotherapy for OCD (Abramowitz et al., 2005), and relative to waitlist conditions for anxiety disorders (Ale et al., 2015) and PTSD (Huang et al., 2022). Moreover, despite clients experiencing temporary distress during ET, it is frequently reported as useful (Cox et al., 1994) and is well tolerated (Carpenter et al., 2018; Tuerk et al., 2011).

## The Underutilisation of ET

Despite half-a-century of clinical trials and reviews establishing the effectiveness of ET (e.g., Carpenter et al., 2018) and solidifying its place as the treatment of choice for anxiety-related presentations (e.g., Katzman et al., 2014), ET remains underutilised in the routine care of anxiety-related presentations. For instance, only 37% of clinicians in community mental health settings endorse routinely using ET with clients with anxiety-related presentations (Becker-Haimes et al., 2017). This means that in routine practice ET is implemented at a rate comparable to that of non-evidence-based techniques including art therapy for the treatment of anxiety disorders, OCD, and PTSD (i.e., 17% to 28%; Hipol & Deacon, 2013). This underuse occurs across the lifespan with Whiteside, Sattler et al. (2016a, 2016b) finding 76% of youth with anxiety-related presentations treated outside specialty anxiety clinics are never introduced to ET. Beyond its underuse, even when ET is implemented, it is often done sub-optimally. For example, it is more frequently delivered as therapist-instructed homework instead of the gold-standard, therapist-assisted, within-sessions exposure (Sars & van Minnen, 2015). This underutilisation of ET is complex and may occur for many reasons, including clinicians’ perceptions of ET’s safety, tolerability, and applicability (Blakey et al., 2018). In the context of treating clients from different developmental subgroups, unique barriers to ET implementation may exist. Indeed, Becker and colleagues (2018) in their study of 209 CBT trainees and psychotherapists in Germany found that clinicians treating youth hold more negative ET attitudes than those treating adults. Given the prevalence of negative perceptions towards ET (Blakey et al., 2018) and reflecting broader psychotherapy implementation literature (Waller & Turner, 2016), research to date has primarily focused on clinician-level determinants (Deacon & Farrell, 2013). Whilst client and contextual determinants have been examined (Harned et al., 2013), they constitute few studies by comparison, and explorations are often limited to relatively few possible determinants. Overall, this underutilisation of ET represents a significant barrier for clients to access effective evidence-based treatment (Abramowitz et al., 2018) and has emerged as an important area of research in the last decade. However, there have been few efforts (e.g., Becker-Haimes et al., 2019; Ringle et al., 2015) to integrate evidence on known implementation determinants addressing clinician, client, and system predictors of ET underutilisation.

## The Contribution of Implementation Science

Implementation science refers to the use of the scientific method to promote the systematic uptake of research into evidence-based practice (Eccles & Mittman, 2006). Applying such an approach to identifying the most important predictors of ET use across client subgroups may support the development of innovative and more effective intervention strategies to improve its implementation. Several theoretical frameworks have been developed to understand the determinants of knowledge implementation (Birken et al., 2017b). One such framework, the Theoretical Domains Frameowrk (TDF) comprehensively integrates 128 explanatory constructs from 33 distinct theories (e.g., Theory of Planned Behaviour) to identify14 domains related to healthcare provider behaviour change (Cane et al., 2012; Michie et al., 2005). These include knowledge; skills; social/professional role and identity; beliefs about capabilities; optimism; beliefs about consequences; reinforcement; intentions; goals; memory, attention, and decision processes; environmental context and resources; social influences; emotion; and behavioural regulation. Importantly, these domains can be translated via the Capability Opportunity and Motivation model of behaviour (COM-B; Michie et al., 2011) to identify targeted interventions on the Behaviour Change Wheel (BCW; Michie et al., 2014) to improve implementation. It has been used in a range of settings to identify determinants of evidence implementation for acute lower back pain, hand hygiene, blood transfusion, tobacco use prevention (Cane et al., 2012), remote psychological practice (Faija et al., 2020), and shared decision-making (Hayes et al., 2019). This theoretical breadth and relationship to the BCW make the TDF particularly suited to the goal of informing strategies for improving the implementation of ET in practice.

The previous absence of such a theoretical foundation in the exploration of ET implementation, like in the broader psychotherapy implementation literature (Williams & Beidas, 2019), has created a body of evidence with competing ideas, hindering the translation of evidence into improved practice. For example, whilst training in ET appears a promising method of improving knowledge, attitudes, and self-efficacy (Trivasse et al., 2020), training alone does not translate directly to behaviour change (Cucciare et al., 2008; Kemp, 2019), due to the influence of several other factors (Godin et al., 2008). As such, the use of implementation science, and the TDF specifically, to identify, prioritise, and synthesise implementation determinants, will assist to close the research-practice gap by identifying clear targets for intervention (Ogden & Fixsen, 2014).

## The Current Review

To date, explorations of the determinants of ET implementation in routine practice have been largely atheoretical and rarely examined if determinants differ across client presentations (i.e., anxiety disorders, OCD, and PTSD) and developmental subgroups (i.e., youth and adults). To address these limitations, this systematic review aimed to synthesise the existing evidence on the determinants of ET implementation for treating anxiety-related presentations across the lifespan guided by the TDF, including identifying theoretical gaps for future research.

## Objectives of the Review

One primary review question was explored with three secondary questions. Specifically, which domains of the Theoretical Domains Framework are related to the implementation of exposure therapy by mental health clinicians in the treatment of anxiety-related presentations?

1. How do these domains differ between anxiety disorders (i.e., separation anxiety disorder, selective mutism, specific phobia, social anxiety disorder, panic disorder, agoraphobia, generalised anxiety disorder), obsessive-compulsive disorder, and post-traumatic stress disorder?
2. How do these domains differ between youth (i.e., ≤ 17 years old) and adults (i.e., ≥ 18 years old) with anxiety-related presentations?
3. Which domains remain unexplored or underexplored as possible determinants of exposure therapy implementation in the current literature?

## Methods

The protocol for this review was prospectively registered (CRD42022308100) on PROSPERO (Centre for Reviews and Dissemination, 2023). The search and reporting were conducted in accordance the Preferred Reporting Items for Systematic Reviews and Meta-Analyses (PRISMA) statement (Page et al., 2021).

### Search Strategy

Search terms and their synonyms addressing implementation, ET, and client presentations were combined into a single search query, alongside appropriate subject headings. The search strategy was then piloted in APA PsycInfo and then refined. Relevant databases (APA PsycInfo [Ovid], CINAHL Complete [EBSCO], Embase, ProQuest, PubMed, Scopus, and Web of Science) and a search engine (Google Scholar) were searched in May 2023. Where possible, searches were limited to results that were peer-reviewed and in the English language. All results were recorded from scholarly databases, whilst Google Scholar results were recorded until saturation (i.e., 800 results; Haddaway et al., 2015a). A detailed search translation summary for each database and search engine can be found in Online Resource 1. Record duplicates were automatically removed using EndNote, before been imported into the systematic review data management tool, Covidence (2023), which automatically removed further duplicates. To identify other relevant studies, prior and derivative works for eligible studies were searched using Connected Papers (2023) which uses machine learning based on co-citation and bibliographic coupling.

### Eligibility Criteria

Studies were included if they: (a) operationalised ET within a CBT theoretical orientation; (b) assessed the implementation of any form of ET (i.e., as a therapeutic approach; not solely a resource or workbook) as a distinct modality by mental health clinicians (including student clinicians completing relevant practical clinical training) for anxiety, OCD, or PTSD; (c) reported primary quantitative data on how a variable related to the actual implementation of ET (i.e., utilisation; not adherence, efficacy, or effectiveness) using inferential statistics; (d) were published in a peer-reviewed journal; and, (e) were written in the English language. Studies were excluded if they: (a) constituted grey literature (i.e., abstracts, books or book chapters, commentaries, dissertations, editorials, letters, and presentations); (b) were a secondary review of one or more primary studies; (c) were a case study, case series, or N of 1 design; or (d) had an unavailable full-text. No date restrictions were applied. For all inclusions, Ulrich’s Periodicals Directory was used to confirm publication in a peer-reviewed journal.

### Selection Process

Eligibility criteria were independently piloted by two reviewers on subsets of relevant records (Büchter et al., 2020). Initially, the eligibility criteria were piloted by the reviewers on the first 5% of records in the titles and abstract screening phase. These criteria were then piloted by the reviewers on 10% of the records screened in the full-text phase. Following each step, discrepancies were discussed, resolved, and the criteria refined to maximise accuracy. Additional rounds of full-text screening of 10% of the records were completed until 100% agreement was attained signifying the primary reviewer had consistent interpretation and application of the selection criteria. The primary reviewer then independently engaged in a two-stage eligibility screening of the remaining (a) titles and abstracts and (b) full-texts, with ineligible records excluded at each stage.

### Data Extraction and Quality Appraisal

The data extraction form and quality appraisal tool were independently piloted in the same manner as the eligibility criteria. No discrepancies were identified between the two reviewers. The data items extracted from the included papers were the study’s citation information, focus (e.g., aim, therapy), research design, sample (including presentation and developmental subgroup), and results on ET implementation (i.e., measures, analyses, and results). Each result was coded to a client presentation (i.e., anxiety disorders, OCD, or PTSD), developmental subgroup (i.e., youth or adults), and TDF domain. Results that could not be coded to a single presentation, developmental subgroup, or TDF domain either because they covered multiple groups or were unspecified were coded as anxiety-related presentations, lifespan, or unclassified, respectively. These codes were defined in a coding manual, with TDF domains guided by their original conceptualisation, definitions, and constructs (Cane et al., 2012). Some constructs (e.g., negative beliefs about ET) overlapped with several domains but were allocated to a single, dominant domain (e.g., beliefs about consequences).

Given the wide array of designs deemed relevant to the review questions, the Mixed Methods Appraisal Tool (MMAT; Hong et al., 2018) was used by the two reviewers to independently appraise the quality of each study across two universal screening questions and five core criteria that are specific to each design (i.e., quantitative randomised controlled trials, quantitative non-randomised, quantitative descriptive, and mixed-methods; Hong et al., 2018). The number of the five core design-specific criteria met on the MMAT were used to calculate a percentage indicative of overall quality, which was categorised as very low (i.e., ≤ 20%), low (i.e., 40%), moderate (i.e., 60%), high (i.e., 80%), and very high (i.e., 100%).

### Individual Results and Synthesis

The quantity and characteristics of included studies were summarised alongside the quality appraisal, using descriptive statistics. Informed by a pragmatic research philosophy and the heterogeneity of the results identified, as well as to assist the identification of theoretical gaps in the literature, the quantity of studies and results exploring each TDF domain per presentation and developmental subgroups were narratively synthesised. Using established guidelines (Rodgers et al., 2009) the quality appraisal of the included studies was summarised. The extracted results were then tabulated and grouped using the TDF domains. Under each presentation (i.e., anxiety disorders, OCD, PTSD, and anxiety-related presentations) and developmental subgroup (i.e., youth and adults), the body of results were organised from the most to least explored domains, with constructs under each described with reference to the consistency of results of statistical significance regarding their relationship with ET use. When a single study presented multiple results (e.g., for different ET variants) relevant to the same construct, presentation, and developmental subgroup, these were tabulated together, and the construct was recorded as related if any of the relationships were significant. Additionally, univariate and multivariate analyses of the same relationship were collapsed and only the multivariate result was used. No arbitrary cut-offs were used for the classification of results based on the percentage of significant results to avoid oversimplifying the literature by underemphasising important variations (Haddaway et al., 2015a, 2015b) including heterogeneity in the constructs explored.

## Results

### Included Studies

**Figure 1:**
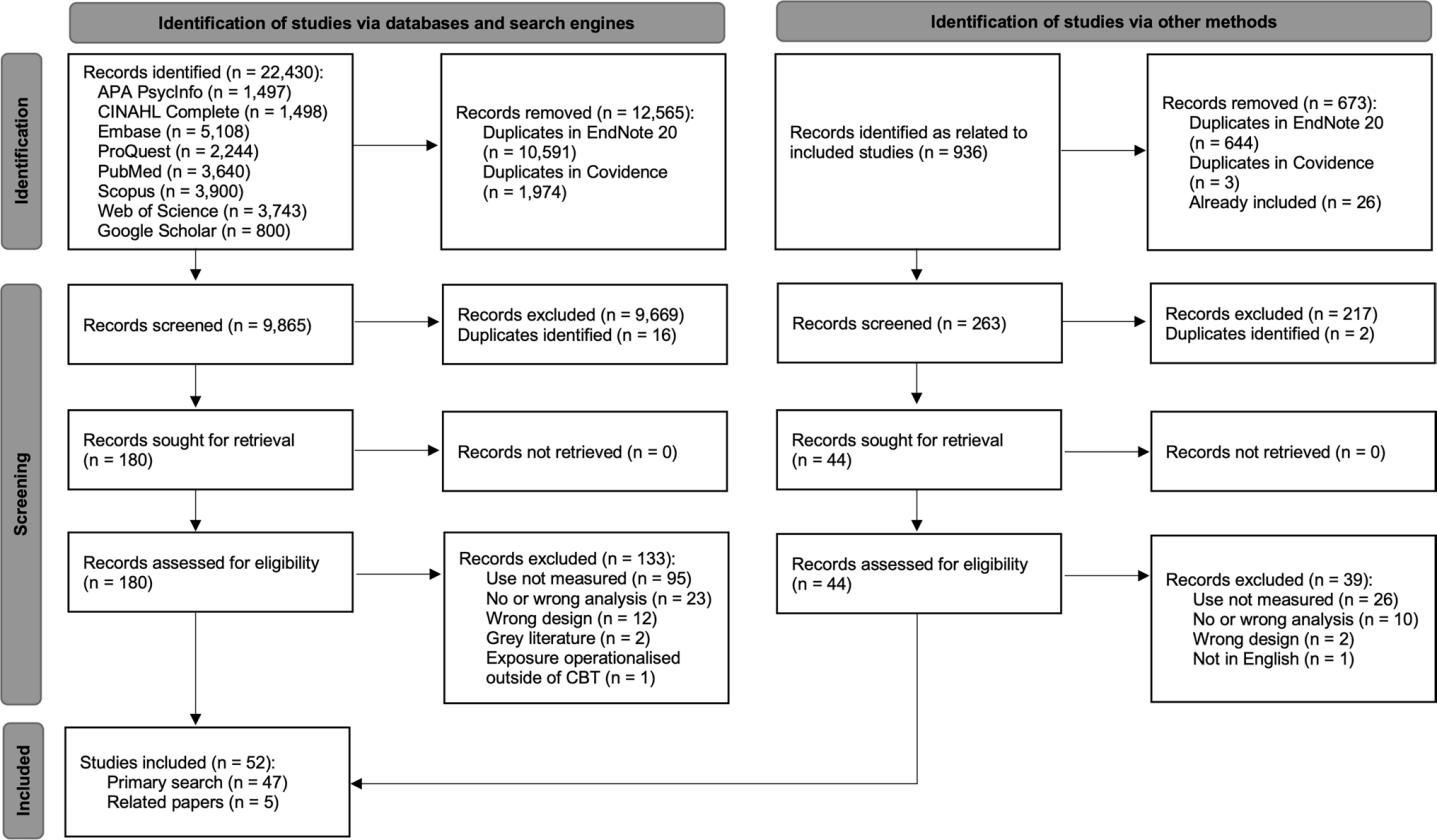
Preferred Reporting Items for Systematic Reviews and Meta-Analyses (PRISMA) Flow Diagram (Page et al., 2021)

The search retrieved 22,430 records, of which 12,565 were duplicates. After title and abstract screening, a further 9,685 records were excluded. The remaining 180 full-texts were screened, of which 133 were excluded, leaving 47 included studies. These studies were entered into Connected Papers (2023) producing 936 related records, of which 647 were duplicates and 26 were already included in the review. Screening the remaining 263 resulted in five additional relevant studies. Thus, 52 studies were included in this review (see Fig. 1).

### Study Characteristics and Quality Appraisal

The characteristics of the included studies are summarised in Table 1 (see Online Resource 2 for detailed characteristics). Over half of the studies (*n *= 28) were quantitative descriptive designs (e.g., surveys), 12 used a quantitative non-randomised design (e.g., cohort studies), seven were quantitative randomized controlled trials, and five utilised mixed-methods (e.g., convergent designs). All studies were conducted in high-income Westernised countries, i.e., the United States (*n *= 33), Europe (*n *= 11), Australia and New Zealand (*n *= 7), and the United States and United Kingdom (*n *= 1). Half of the studies examined determinants in specific diagnostic presentations (i.e., PTSD [*n *= 20], anxiety disorders [*n *= 3], OCD [*n *= 3]), while the remainder used mixed anxiety-related disorder samples (*n *= 26) and only six of these stratified their results into specific presentations. Over half examined determinants in either adult (*n *= 19; all unspecified age ranges) or youth clients (*n *= 11; ages ranged from 7 to 17 years), with the remaining related to clients across the lifespan (*n *= 22). Studies included samples of clinicians (*n *= 42; samples sizes ranged from 24 to 1,034), clients (*n *= 3; samples sizes ranged from 554 to 265,566), or clinicians and clients (*n *= 7; clinician sample sizes ranged from 4 to 103; client sample sizes ranged from 25 to 242). The samples of clinicians tended to be mostly female (frequencies ranged from 60% to 100%) and psychologists (frequencies ranged from 14% to 100%). Only ten samples were reported where another profession (e.g., social workers, counsellors, therapists) had greater representation than psychologists. Most studies (77%) utilised self-report to assess ET use; the remainder (21%) reviewed client records or session recordings, while one quantified interview responses.

**Table 1:** Summary of Included Studies Ordered by Presentation then Developmental Subgroup of Interest

Study	Design	Method	Presentation	Developmental Subgroup
(Whiteside et al., 2023)	Quantitative randomised controlled trial	–	Anxiety disorders (e.g., generalised anxiety disorder, specific phobia, social anxiety disorder, separation anxiety disorder).	Youth (9 to 17 years).
(Klan et al., 2017)	Quantitative non-randomised	Cohort	Anxiety disorders (agoraphobia).	Adults (unspecified).
(Becker et al., 2018)	Quantitative descriptive	Survey	Anxiety disorders (panic disorder and/or agoraphobia).	Lifespan (unspecified).
(Kannis-Dymand et al., 2022)	Quantitative descriptive	Survey	Anxiety disorders.	Lifespan (16 years or older).
(Keleher et al., 2020)	Quantitative descriptive	Survey	OCD.	Youth (unspecified).
(Hertz et al., 2023)	Quantitative non-randomised	Cross-sectional	OCD.	Adults (unspecified).
(Moritz et al., 2019)	Quantitative descriptive	Survey	OCD.	Lifespan (unspecified).
(Cook et al., 2015a, 2015b, 2015c)	Mixed methods	Sequential	PTSD.	Adults (unspecified).
(Cook et al., 2015a, 2015b, 2015c)	Quantitative descriptive	Survey	PTSD.	Adults (unspecified).
(Cook et al., 2014)	Mixed methods	Convergence	PTSD.	Adults (unspecified).
(Cook et al., 2013)	Mixed methods	Sequential	PTSD.	Adults (unspecified).
(Cook et al., 2020a, 2020b)	Mixed methods	Sequential	PTSD.	Adults (unspecified).
(Cook et al., 2015a, 2015b, 2015c)	Quantitative descriptive	Survey	PTSD.	Adults (unspecified).
(Cook et al., 2020a, 2020b)	Quantitative descriptive	Survey	PTSD.	Adults (unspecified).
(Finley et al., 2015)	Quantitative descriptive	Survey	PTSD.	Adults (unspecified).
(Foa et al., 2020)	Quantitative randomised controlled trial	-	PTSD.	Adults (unspecified).
(Garcia et al., 2020)	Quantitative descriptive	Survey	PTSD.	Adults (unspecified).
(Kline et al., 2021)	Quantitative descriptive	Survey	PTSD.	Adults (unspecified).
(Maguen et al., 2019)	Quantitative non-randomised	Cross-sectional	PTSD.	Adults (unspecified).
(Rosen et al., 2019)	Quantitative non-randomised	Cohort	PTSD.	Adults (unspecified).
(Rosen et al., 2017)	Quantitative descriptive	Survey	PTSD.	Adults (unspecified).
(Ruzek et al., 2017)	Quantitative non-randomised	Repeated measures	PTSD.	Adults (unspecified).
(Becker et al., 2004)	Quantitative descriptive	Survey	PTSD.	Lifespan (unspecified).
(Harned et al., 2021)	Quantitative non-randomised	Repeated measures	PTSD.	Lifespan (unspecified).
(Sherrill et al., 2021)	Quantitative non-randomised	Repeated measures	PTSD.	Lifespan (unspecified).
(van Minnen et al., 2010)	Quantitative randomised controlled trial	-	PTSD.	Lifespan (unspecified).
(Wade et al., 2020)	Quantitative non-randomised	Repeated measures	PTSD.	Lifespan (unspecified).
(Becker-Haimes et al., 2017)	Quantitative descriptive	Survey	Anxiety-related presentations (combined anxiety disorders, OCD, and PTSD).	Youth (unspecified).
(de Jong et al., 2020)	Quantitative descriptive	Survey	Anxiety-related presentations (unspecified).	Youth (unspecified).
(Reid et al., 2017)	Quantitative descriptive	Survey	Anxiety disorders, OCD, and PTSD.	Youth (7 to 17 years).
(Reid et al., 2018)	Quantitative descriptive	Survey	Anxiety disorders, OCD, and PTSD.	Youth (7 to 17 years).
(Vande Voort et al., 2010) ^a^	Quantitative non-randomised	Cross-sectional	Anxiety-related presentations (combined anxiety disorders and OCD).	Youth (6 to 18 years).
(Whiteside et al., 2022a, 2022b)	Quantitative non-randomised	Repeated measures	Anxiety-related presentations (combined anxiety disorders and OCD).	Youth (7 to 17 years).
(Whiteside et al., 2022a, 2022b) ^a^	Quantitative randomised controlled trial	-	Anxiety-related presentations (combined anxiety disorders and OCD).	Youth (8 to 18 years).
(Whiteside et al., 2016a, 2016b)	Quantitative descriptive	Survey	Anxiety-related presentations (combined anxiety disorders, OCD, and PTSD).	Youth (unspecified).
(Whiteside et al., 2016a, 2016b)	Quantitative non-randomised	Cross-sectional	Anxiety-related presentations (combined anxiety disorders, OCD, and PTSD).	Youth (7 to 17 years).
(Chen et al., 2022)	Quantitative descriptive	Survey	Anxiety-related presentations (unspecified).	Adults (unspecified).
(Rowe & Kangas, 2020)	Quantitative descriptive	Survey	Anxiety-related presentations (combined anxiety disorders and OCD).	Adults (unspecified).
(Becker‐Haimes et al., 2020)	Quantitative descriptive	Survey	Anxiety-related presentations (unspecified).	Lifespan (unspecified).
(Deacon et al., 2013) ^b^	Q uantitative non-randomised	Repeated measures	Anxiety-related presentations (unspecified).	Lifespan (unspecified).
(Harned et al., 2013)	Quantitative randomised controlled trial	-	Anxiety-related presentations (unspecified).	Lifespan (unspecified).
(Harned et al., 2014)	Quantitative randomised controlled trial	-	Anxiety-related presentations (combined anxiety disorders and PTSD).	Lifespan (unspecified).
(Harned et al., 2011)	Quantitative randomised controlled trial	-	Anxiety-related presentations (unspecified).	Lifespan (unspecified).
(Hipol & Deacon, 2013)	Quantitative descriptive	Survey	Anxiety-related presentations (combined anxiety disorders, OCD, and PTSD).	Lifespan (unspecified).
(Meyer et al., 2020)	Quantitative descriptive	Survey	Anxiety-related presentations (unspecified).	Lifespan (unspecified).
(Moses et al., 2022)	Mixed methods	Sequential	Anxiety-related presentations (combined anxiety disorders, OCD, and PTSD), anxiety disorders, OCD, and PTSD.	Lifespan (unspecified).
(Moses et al., 2021)	Quantitative descriptive	Survey	Anxiety-related presentations (combined anxiety disorders, OCD, and PTSD), anxiety disorders, OCD, and PTSD.	Lifespan (unspecified).
(Parker & Waller, 2019)	Quantitative descriptive	Survey	Anxiety-related presentations (unspecified).	Lifespan (unspecified).
(Pittig et al., 2019)	Quantitative descriptive	Survey	Anxiety-related presentations (combined anxiety disorders, OCD, and PTSD).	Lifespan (unspecified).
(Sars & van Minnen, 2015)	Quantitative descriptive	Survey	Anxiety disorders and OCD.	Lifespan (unspecified).
(Schumacher et al., 2018)	Quantitative descriptive	Survey	Anxiety disorders and PTSD.	Lifespan (unspecified).
(Živčić-Bećirević et al., 2019)	Quantitative descriptive	Survey	Anxiety-related presentations (unspecified).	Lifespan (unspecified).

*Note.* Lifespan is defined as unspecified developmental subgroups or a combination of youth and adult developmental subgroups. Anxiety-related presentations are defined as an unspecified or specified combination of anxiety disorders, OCD, and PTSD.

^a^ These studies were defined as relevant to youth, not lifespan, due to the predominant age-range of the sample.

^b^ This article reported on three separate but related studies. Only the results of the third study were relevant to this review and were extracted and appraised.

The quality appraisal of included studies is found in Online Resource 3. The quality of the studies varied greatly, although two-thirds possessed at least moderate quality (60% of criteria met). Specifically, seven studies possessed *very low*, 11 *low*, 17 *moderate*, 15 *high*, and two *very high *methodological quality. The most frequently identified quality issues for each design included assessors not being blind to the condition (71% of quantitative randomised controlled designs), that samples were unrepresentative of the target population (58% of quantitative non-randomised designs), a high likelihood of response bias (89% of quantitative descriptive designs), and that either the quantitative or qualitative methodological component of mixed-method studies were of poor quality (all mixed-method designs).

### Results Synthesised by Theoretical Domains Framework Domains

The original TDF domains are presented in Table 2, with the operationalisation of each domain for this review and the coded constructs. The frequency of results per TDF domain, by presentation and developmental subgroup are presented and described below and are presented in Online Resource 4. For further detail, the domains explored in each study are presented in Online Resource 5 and the individual results are summarised in Online Resource 6. Most results (*n* = 389) were mapped to the TDF. All domains were represented except for *optimism*, *reinforcement*, and *memory, attention, and decision processes*. Four domains (*knowledge*, *social/professional role and identity*, *beliefs about consequences*, and *environmental context and resources*) accounted for 70% of the mapped results, whilst the least represented domain was *behavioural regulation*. *Social/professional role and identity* and *environmental context and resources* appear to be particular focuses of the existing literature, with each contributing over one-fifth of results. Most other domains (*skills*, *beliefs about capabilities*, *intentions*, *goals*, *social influences*, and *emotion*) attracted at most 8% of results from the existing literature. Within each domain, there was considerable variability in the operationalisation and measurement of constructs, as seen in Table 2. Five results (e.g., positive attitudes towards multiple components of evidence-based practice) could not be classified to the TDF as they assessed a range of domains. This uneven representation also affected how presentations and developmental subgroups were explored with PTSD (46%) representing over three times more results than anxiety disorders (14%) and OCD (11%), and adult samples (44%) contributing over two times more results than youth (20%).

**Table 2:** Original Conceptualisation of TDF (Cane et al., 2012) and Operationalisation for the Review

TDF Domain	Definition and Constructs (Cane et al., 2012)	Current Operationalisation	Constructs Coded ^a^
Knowledge	An awareness of the existence of something. Includes knowledge (i.e., knowledge of condition/scientific rationale), procedural knowledge, and knowledge of task environment.	An awareness of the existence of therapeutic techniques including ET (i.e., knowledge of theory, rationale for use, application).	Knowledge of ET; unspecified ET training; unspecified CBT training; post-qualification accreditation.
Skills	An ability or proficiency acquired through practice. Includes skills, skill development, competence, ability, interpersonal skills, practice, and skill assessment.	An ability or proficiency in therapeutic techniques including ET acquired through practice.	Practical training in ET; experience using ET; experience treating anxiety-related presentations; general clinical experience.
Social/Professional Role and Identity	A coherent set of behaviours and displayed personal qualities of an individual in a social or work setting. Includes professional identity, professional role, social identity, identity, professional boundaries, professional confidence, group identity, leadership, and organisational commitment.	A coherent set of individual or group behaviours and displayed personal qualities in a social or work setting.	Clinician factors: Qualification in psychology (e.g., clinical psychology, other); registration status as a psychologist (e.g., provisional, general, with endorsement); education; profession; therapeutic theoretical orientation; self-identified specialisation in anxiety; demographics (i.e., age, gender). Client factors: Demographics (i.e., age, gender, race, relationship status, ethnicity, education); military experience (i.e., service branch, service component, rank, multiple deployments, deployment in combat zone).
Beliefs about Capabilities	Acceptance of the truth, reality, or validity about an ability, talent, or facility that a person can put to constructive use. Includes self-confidence, perceived competence, self-efficacy, perceived behavioural control, beliefs, self-esteem, empowerment, and professional confidence.	Acceptance of the truth, reality, or validity of about an ability, talent, or facility relating therapeutic techniques including ET that a person can put to constructive use.	Self-efficacy using ET; self-efficacy using other therapeutic techniques; self-esteem; perceived control over treatment plan and schedule; self-efficacy receiving ET referrals; perceived resilience of children with anxiety.
Optimism	The confidence that things will happen for the best or that desired goals will be attained. Includes optimism, pessimism, unrealistic optimism, and identity.	The confidence that therapeutic techniques including ET will have the best outcome or that desired goals will be attained.	None.
Beliefs about Consequences	Acceptance of the truth, reality, or validity about outcomes of a behaviour in a given situation. Includes beliefs, outcomes expectancies, characteristics of outcome expectancies, anticipated regret, and consequents.	Acceptance of the truth, reality, or validity about outcomes relating to therapeutic techniques including ET in a given situation.	Clinician factors: Negative beliefs about ET and CBT; perceived utility and risks of ET; perceived treatment credibility of ET; perceived superiority of ET to existing practices; perceived observability of ET results to others; perceived utility of ET treatment components; perceived utility and risks of other therapeutic techniques. Clinician and client factors: Outcome expectancies.
Reinforcement	Increasing the probability of a response by arranging a dependent relationship, or contingency, between the response and a given stimulus. Includes rewards (proximal/distal, valued/not valued, probable/improbable), incentives, punishment, consequents, reinforcement, contingencies, and sanctions.	Increasing the probability of using therapeutic techniques including ET by arranging a dependent relationship, or contingency, between the response and a given stimulus.	None.
Intentions	A conscious decision to perform a behaviour or a resolve to act in a certain way. Includes stability of intentions, stages of change model, and transtheoretical model and stages of change.	Motivation to use therapeutic techniques including ET.	Openness to and willingness to use ET; completion of an intervention aimed at improving motivation to use ET.
Goals	Mental representations of outcomes or end states that an individual wants to achieve. Includes goals (distal/proximal), goal priority, goal/target setting, goals (autonomous/controlled), action planning, and implementation intention.	Mental representations of outcomes or end states (including implementation intention) for therapeutic techniques including ET that an individual wants to achieve. Includes actual use of other techniques as a reflection of goal priority.	Use of other techniques; use of therapist safety behaviours; likelihood of excluding clients from ET due to their characteristics; intention to use ET; allocation to a treatment condition delivering parent-coached ET; personal preference for ET.
Memory, Attention, and Decision Processes	The ability to retain information, focus selectively on aspects of the environment, and choose between two or more alternatives. Includes memory, attention, attention control, decision-making, and cognitive overload/tiredness.	Factors influencing the retention of information, selective focusing on aspects of the environment, and choice between using therapeutic techniques and ET.	None.
Environmental Context and Resources	Any circumstance of a person’s situation or environment that discourages or encourages the development of skills and abilities, independence, social competence, and adaptive behaviour. Includes environmental stressors, resources/material resources, organisational culture/climate, salient events/critical incidents, person-environment interaction, and barriers and facilitators.	Circumstance of a person’s situation, context, or environment that can influence the development of skills and abilities, independence, social competence, and adaptive behaviour relating to using therapeutic techniques including ET.	Clinician factors: Current frequency of exposure to anxiety-related presentations; perceived impact of client’s resistance to change on ability to use ET; workload; consistency of prolonged exposure with the priorities of the adopter/service; practice setting; average anxiety treatment length; more frequently engaging in group, relative to individual therapy. Client factors: Comorbidities; residence relative to urban centres; military sexual trauma; smoking status; current medications; visit to Veterans Health Administration following implementation of evidence-based practices; number of non-evidence-based therapy sessions received before ET; PTSD diagnosis related to military service; diagnoses; recent hospitalisation; level of impairment due to anxiety. System factors: Organisational structure and support; workforce size; organisational turnover; demands on residential treatment programmes; availability of resources for prolonged exposure; practical barriers to ET use; availability of interested clients; working within a PTSD specialty clinic; working within an integrated behavioural health setting.
Social Influences	Those interpersonal processes that can cause individuals to change their thoughts, feelings, or behaviours. Includes social pressure, social norms, group conformity, social comparisons, group norms, social support, power, intergroup conflict, alienation, group identity, and modelling.	Interpersonal processes that can cause individuals to change their thoughts, feelings, or behaviours on using therapeutic techniques including ET.	Clinician factors: Social network; attachment style. Clinician and client factors: Therapeutic alliance. System factors: Organisational culture of implementation; leadership articulating goals to implement prolonged exposure; organisation prioritising cognitive processing and other therapies; organisational politics; fair treatment of clinicians by superiors; telephone consultations with prolonged exposure experts; group supervision on ET; amount of supervision given or received monthly; supervisors’ recommendation to use ET.
Emotion	A complex reaction pattern, involving experiential, behavioural, and physiological elements, by which the individual attempts to deal with a personally significant matter or event. Includes fear, anxiety, affect, stress, depression, positive/negative affect, and burnout.	A complex reaction pattern, involving experiential, behavioural, and physiological elements by which the individual attempts to deal with a personally significant matter or event.	Clinician factors: Intuitive appeal of evidence-based practice; anxious and depressive symptoms; anxiety and disgust sensitivity; distress and avoidance relating to exposure. Client factors: Anxiety and depressive symptoms; distress.
Behavioural Regulation	Anything aimed at managing or changing objectively observed or measured actions. Includes self-monitoring, breaking habit, and action planning.	Anything aimed at managing or changing objectively observed or measured actions, including using therapeutic techniques including ET.	Perceived divergence between evidence-based and current practices; willingness to use evidence-based practice if required to.

^a^Constructs relate to clinicians unless otherwise specified.

### Presentations

**Figure 2:**
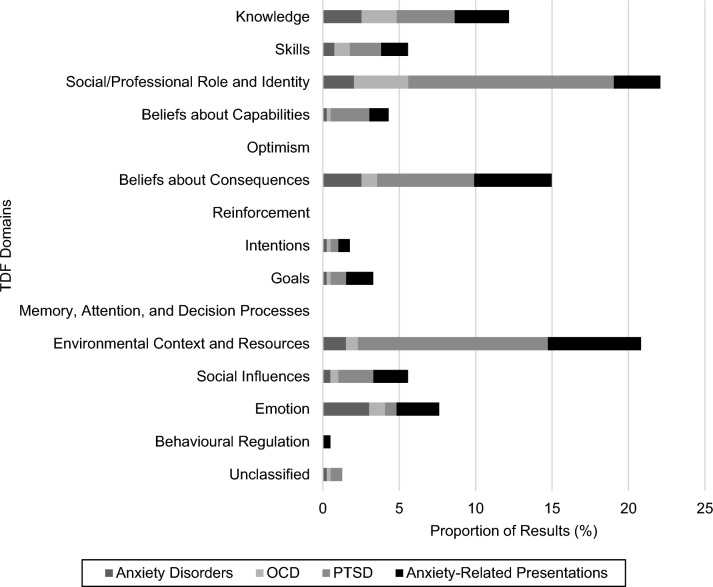
Results Exploring TDF Domains Categorised by Presentation. *Note.* Mapped results totalled 389 with 5 additional results unclassifiable. Anxiety-related presentations are defined as an unspecified or specified combination of anxiety disorders, OCD, and PTSD

The spread of results across domains of the TDF, per presentation, is presented in Figure 2.

#### Anxiety Disorders

Fifty-five results from 10 studies explored the use of ET for anxiety disorders. All of these studies used low quality, descriptive or non-randomised designs, with only one study rated as good quality. Overall, this subset of literature only met 54% MMAT criteria, which approximates moderate quality. Over half of the results (58%) pertained to clients from across the lifespan, with the remainder using samples of youth (18%) and adults (24%). *Emotion* was the most frequently explored domain. Five results exploring client’s anxious symptoms and suggested they were unrelated to ET use, whilst results examining other client emotions (e.g., depressive symptoms) and clinician emotions (e.g., anxiety) were too few to draw meaningful conclusions. In contrast, ten results examined the domain of *knowledge* (i.e., unspecified ET training, knowledge of ET) and widely found positive relationships with ET use. Likewise, ten results examined clinicians’ *beliefs about consequences* of ET (including both perceived risks and credibility) and were frequently associated with ET implementation, in particular, more negative beliefs about ET were associated with less use in five results.

Both the domains of *social/professional role and identity*, and *environmental context and resources* were investigated, but examined a broad range of constructs were examined. This resulted in the inability to draw meaningful conclusions. Trends suggested that most results categorised under both *social/professional role and identity* and *environmental context and resources* were unrelated or inconsistently related to ET use. The exceptions, however, were clinician age and perceived practical barriers to ET use, which tended to be negatively related to implementation, whereas current frequency of exposure to anxiety presentations appeared to be positively related. A small proportion of studies examined *skills*, *social influences*, *beliefs about capabilities*, *intentions*, or *goals*, with limited results. Trends suggested that clinicians’ self-efficacy and willingness to use ET, personal preference for ET, and experience treating anxiety presentations tended to be positively related to ET use. A result that was unable to be classified to one theoretical domain found that more barriers (ranging in focus from resources to consequences) perceived by clinicians to ET use was negatively related to use (Reid et al., 2017).

#### OCD

Forty-four results from eight studies related to ET use for the treatment of OCD. On average these studies met 60% of MMAT criteria which is indicative of moderate quality. However, six studies used descriptive designs, including surveys, thus a high proportion of results came from low quality studies with significant risk of bias. Like with anxiety disorders, most results (57%) came from clients from across the lifespan, with the rest pertaining to youth (32%) or adults (11%). The largest proportion of results related to *social/professional role and identity* and a broad range of constructs were examined (e.g., client and clinician demographics, clinician therapeutic theoretical orientation). This resulted in a low volume of results examining similar constructs and a lack of meaningful trends. Whilst most demographics appeared unrelated to ET use, client age tended to be negatively related to ET use, whilst being a psychologist or specialising in anxiety was seemingly positively related to ET use. Under the domain of *knowledge*, there were inconsistent results related to unspecified ET training, whilst a single result suggested knowledge of ET was positively related to its use.

The less frequently explored domains (i.e., *skills*, *beliefs about consequences*, *emotion*, *environmental context and resources*, *social influences*, *beliefs about capabilities*, *intentions*, and *goals*) were characterised by constructs that were only examined in one or two results, therefore limiting the conclusions that can be drawn from each. These trends indicated that general clinical experience, experience treating OCD, amount of supervision, and supervisor’s recommendations to use ET may be unrelated to ET use. Negative beliefs about ET tended to be negatively related to use in three results, as was clinicians’ avoidance of ET in one result. Self-efficacy, willingness, and a personal preference to use ET trended toward positively related to ET use. Current frequency of exposure to anxiety presentations was inconsistently related to ET use. An unclassified result found that more barriers (ranging in focus from resources to consequences) perceived by clinicians to ET use is negatively related to use (Reid et al., 2017).

#### PTSD

Twenty-five studies reported 181 results related to ET use for PTSD. On average these studies met 50% of MMAT criteria which approximated moderate quality. However, several of these studies analysed clinical notes documenting ET use, reducing the biases of self-report. Most of the results (78%) examined ET use in adults who were military personnel or veterans, which significantly reduces the generalisability of findings to the general community. Only a small subset of results pertained to youth (4%) and clients from across the lifespan (17%).

*Social/professional role and identity* was the most frequently examined domain and included constructs such as client and clinician demographics, client military service, and clinician profession or therapeutic theoretical orientation. Key results indicated that clinicians’ gender as male was positively related to ET use, their therapeutic theoretical orientation was unrelated, and results for clinician profession and client demographics (i.e., relationship status and race) were inconsistent. Other constructs were examined in too few results to draw conclusions. Studies also frequently examined *environmental context and resources*, measuring different constructs including organisational characteristics, exposure to presentations, client comorbidities, and availability of exposure resources. Whilst most constructs were only explored in a limited number of results, key trends emerged including that current frequency of exposure to anxiety and PTSD presentations was seemingly positively related to ET use, whilst demands on residential treatment programmes was unrelated, and results examining clinician workload and clients’ residence relative to urban centres were inconsistent.

A substantial twenty-five results also explored the domain of *beliefs about consequences*. Whilst clinicians’ perceptions of the utility of prolonged exposure was positively related to ET use, negative beliefs on ET were negatively related. Several other constructs were examined in two or less results, with one suggesting that the perceived effectiveness of cognitive processing therapy was negatively related to ET use. Under the domain of *knowledge*, unspecified imaginal and prolonged exposure training was positively related to ET use whilst unspecified ET training had inconsistent results. The remaining domains of *beliefs about capabilities*, *social influences*, *skills*, *goals*, *emotion*, and *intentions* and the constructs within them were only examined to a minimal extent, preventing conclusions from being drawn. Trends suggested that self-efficacy using ET, leadership articulating goals to implement prolonged exposure, weekly telephone consultations with prolonged exposure experts, experience treating PTSD, practical training for prolonged exposure, and intention to implement prolonged exposure appear positively related to ET use. Whilst clinical experience, experience with ET, and clinician anxiety tended to be unrelated to ET use, results examining self-efficacy using prolonged exposure were inconsistent. Three unclassified results that could not be classified to one theoretical domain reported that clinicians making more adaptations to prolonged exposure, holding more positive attitudes towards evidence-based practice, and perceiving more barriers (ranging in focus from resources to consequences) to ET use were all unrelated to ET use.

#### Anxiety-Related Presentations

Twenty-one studies presented 114 results exploring the use of ET for anxiety-related presentations. Whilst the overall quality of this literature was moderate (i.e., 60% of MMAT criteria met), one-third of the studies utilised a higher quality design (i.e., quantitative non-randomised [e.g., repeated measures] or randomised design), thus improving the ability to examine relationships between variables. Nevertheless, this body of evidence failed to stratify the results based on client presentations which significantly limits their generalisability. Most results came from clients across the lifespan (49%), with a subset from youth (39%) and adults (11%). The domain of *environmental context and resources* was the most frequently examined with results reporting that client’s possessing an anxiety or OCD diagnosis was positively related to ET use, whilst a broad anxiety-related presentation was unrelated, and results on a PTSD diagnosis were inconsistent. Organisational climate or culture was unrelated to ET use, whilst clinician workload was inconsistent. Beliefs about consequences was also frequently examined; negative beliefs about ET were negatively related to ET use. Other constructs were infrequently examined and thus conclusions were unable to be drawn.

Under the domain of *knowledge*, many constructs were investigated (e.g., knowledge of mechanisms of ET, knowledge of ET). Six results explored the relationship of unspecified ET training to ET use, reporting no relationship. Likewise, results under the *social/professional role and identity* (e.g., client and clinician demographics, clinician profession) and *emotion* domains (e.g., clinician stress, client anxiety) were inconsistent (e.g., for clinician therapeutic theoretical orientation and anxiety). Similar issues hindered conclusions being drawn from the results categorised under the *social influences* (e.g., organisational culture, supervision) and *skills* domains (e.g., self-report expertise in parent-coached ET, online practical training in ET) although clinicians’ attachment styles and general clinical experience were unrelated to ET use based on four results. The remaining domains (i.e., *goals*, *beliefs about capabilities*, *intentions*, and *behavioural regulation*) were less frequently examined and most results could not be sensibly grouped thus limiting the generalisability of the trends. However, trends suggested that self-efficacy using ET appeared to be positively related to its use. Clinicians who endorsed a higher willingness to use evidence-based practice if required to tended to use ET less. Interventions aimed at improving motivation to use ET (e.g., motivational interviewing calls) and the self-reported divergence between evidence-based and current practices appeared to be unrelated to ET use. The results on the use of relaxation techniques were inconsistent.

### Developmental Subgroups

**Figure 3:**
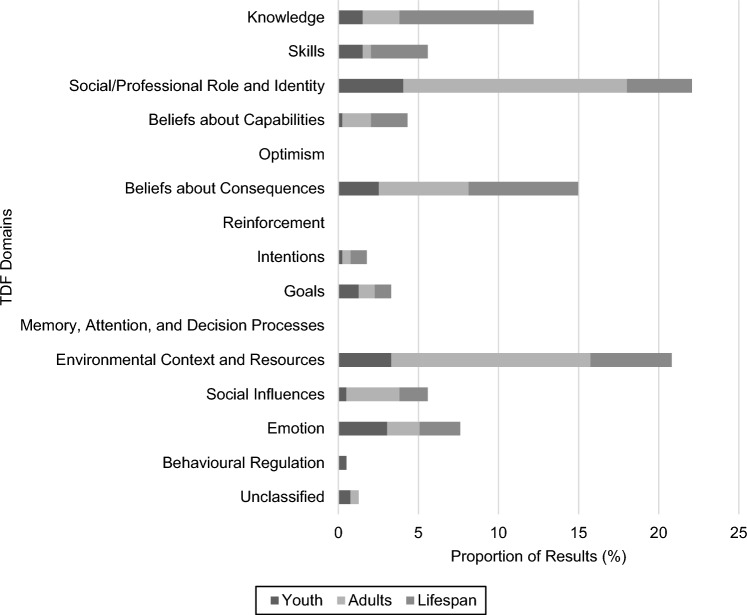
Results Exploring TDF Domains Categorised by Developmental Subgroup. *Note.* Mapped results totalled 389 with 5 additional results unclassifiable. Lifespan is defined as unspecified developmental subgroups or a combination of youth and adult developmental subgroups

The spread of results across domains of the TDF, per developmental subgroup, is presented in Figure 3.

#### Youth

Eleven studies reported 77 results that focused on ET use in the treatment of youth. Nearly half of these studies utilised a higher quality non-randomised (e.g., repeated measures) or randomised study design, whilst the remaining two-thirds (which produced most of the results) utilised a descriptive design. On average 62% of MMAT criteria were met suggesting high quality. However, sampling bias and the use of self-reported ET use dominated this body of evidence. Most of the results pertained to anxiety-related presentation (58%) with the remainder relatively evenly split between exploring OCD (18%), anxiety disorders (13%), and PTSD (10%). *Social/professional role and identity* was the most frequently explored domain and whilst some constructs (e.g., clinician age and profession) were only explored in one result, other constructs were examined more frequently. Clinician gender was unrelated to ET use, whilst results for other constructs (e.g., therapeutic theoretical orientation, specialisation in anxiety, education) were inconsistent. The domain of *environmental context and resources* was also frequently examined, but a wide range of constructs were investigated (e.g., workforce size, working in an integrated health setting, workload). This prevented the formation of meaningful conclusions. Trends suggested that an anxiety or OCD diagnosis and working in an anxiety speciality clinic may be associated with greater ET use, whilst organisational culture, workforce size, and the client’s initial impairment due to anxiety may be unrelated.

In comparison, results in the *beliefs about consequences* domain did provide some insights, including that negative beliefs about ET and perceived risks of ET for youth are related to less ET use. Likewise, results exploring *emotion* (e.g., clinician anxiety, depression, and stress; anxiety and disgust sensitivity) were frequently unrelated to ET use. Results exploring the *knowledge* domain which explored unspecified ET training were inconsistent, whilst in the *skills* domain, general clinical experience was unrelated to ET use. The remaining domains (i.e., *goals*, *social influences*, *behavioural regulation*, *beliefs about capabilities*, and *intentions*) were infrequently examined, thus limiting the ability to draw any meaningful inferences. Trends suggested that an organisational culture of implementation, the perceived resilience of children with anxiety, and openness to innovation appeared to be positively related to ET use, whilst those clinicians who used more anxiety management strategies or who endorsed a higher willingness to use evidence-based practice if required to were also less likely to use ET. Three results that could not be classified to one theoretical domain indicated that more barriers (ranging in focus from resources to consequences) perceived by clinicians to ET use produced inconsistent results (Reid et al., 2017).

#### Adults

Nineteen studies reported 173 results that related to the use of ET for the treatment of adults. Overall, the literature was of moderate quality with an average of 55% of the MMAT criteria met. However, the vast majority of these studies (82%) explored this within the treatment of clients with PTSD, relative to anxiety disorders (8%), anxiety-related presentations (8%), and OCD (3%). The *social/professional role and identity* and *environmental context and resources* domains accounted for over half of the results. A large number of variable constructs were classified to the former domain, including clinician demographics and client military service. Most of these could not be aggregated. However, the clinicians’ profession and therapeutic theoretical orientation, and client demographics had inconsistent results pertaining to ET use. This pattern was observed for the *environmental context and resources* domain where constructs investigated included organisational characteristics, exposure to presentation, client comorbidities, and availability of exposure resources. Findings indicated that current frequency of exposure to anxiety and PTSD presentations and working within a PTSD speciality clinic were positively related to ET use, whilst demands on residential treatment programmes was unrelated, and clinician workload and the geographic location of the clients’ residence relative to urban centres were inconsistent.

Findings classified under the *beliefs about consequences* domain were more homogeneous, particularly exploring the utility of prolonged exposure and negative beliefs about ET, which were positively and negatively related to ET use, respectively. Insufficient evidence existed on other constructs (e.g., outcome expectancies, perceived effectiveness of other techniques, perceived risks) thus conclusions could not be made. Other constructs across several other domains were also limited by little evidence. In the *social influences* domain (e.g., leadership articulating implementation goals, weekly telephone consultation with prolonged exposure experts, social network, therapeutic alliance), suggested the clinician’s attachment style was unrelated to ET use. In the *knowledge* domain (e.g., knowledge of ET mechanisms, unspecified CBT training) unspecified prolonged exposure training was positively related to ET use, whilst two unspecified ET training results were inconsistent. Within the *emotion* domain (e.g., clients’ depressive symptoms and distress), clients’ anxious symptoms were unrelated to ET use. Constructs across the remaining domains (i.e., *beliefs about capabilities*, *goals*, *skills*, and *intentions*) were rarely explored. Trends indicated that self-efficacy using prolonged exposure, general clinical experience, and openness and drive to adopt prolonged exposure appeared to be unrelated to ET use, whilst greater self-efficacy receiving referrals for prolonged exposure and intentions to implement prolonged exposure tended to be associated with more ET use. Two results, which could not be classified to one theoretical domain indicated that clinicians’ adaptations made to prolonged exposure and their positive attitudes towards evidence-based practices appeared to be unrelated to ET use.

## Discussion

This review addressed the paucity of synthesised evidence on the determinants of ET use, by systematically identifying evidence on ET use across anxiety-related presentations and developmental subgroups using an implementation science framework - the TDF. This review aimed to determine whether differences existed between these anxiety-related presentations (i.e., anxiety disorders, OCD, and PTSD) and developmental subgroups (i.e., youth and adults), whilst identifying potential determinants of ET use that remain unexplored. These findings may provide a theoretically cohesive guide for future explorations of this research-practice gap and the development of interventions to address it.

### Characteristics and Quality of the Existing Literature

Whilst the body of evidence spanned 52 studies across 19 years, it was dominated by studies with important limitations. Notably, descriptive study designs were prolific in the literature and were conducted entirely in high-income Western countries, severely limiting the ability to generalise findings to jurisdictions with alternate training and healthcare systems. Additionally, over half of the studies did not explore ET implementation in specific client presentations and/or developmental subgroups, limiting the generalisability of the results to specific treatment contexts. Overall, most included studies were of moderate quality or better, but had unrepresentative samples and a high risk of response bias. For example, several studies recruited participants from populations who likely possessed an increased underlying interest in ET, such as those interested in training, psychologists who received specialty therapeutic training, members of cognitive-behavioural associations, and clinicians working in specialty anxiety clinics. Whilst these samples are easily accessible, recruitment of individuals with a potential bias for ET has limited the understanding of the determinants of ET implementation to those most likely to implement it. These characteristics have limited the quality of the conclusions that can be drawn from the literature and generalised to routine clinical care.

### The Determinants of ET Implementation Across the TDF

Although a multitude of implementation science frameworks exist and are utilised for various reasons (Birken et al., 2017b), the TDF was selected to synthesise the evidence as its focuses on understanding provider behaviour and can inform the development of targeted interventions that align to each domain (Birken et al., 2017b). The TDF fulfilled this purpose as a useful framework for collating the existing evidence and illuminating the spread of evidence across the theoretical domains. Whilst the TDF aligned with the aim of the current review, future applications of implementation science frameworks to explorations of ET implementation in both primary and secondary research should carefully consider the purpose, conceptual level, theoretical heritage, and operationalisability of the framework chosen to ensure appropriate frameworks are selected (Birken et al., 2017a).

Overall, nearly 400 results were identified that evaluated clinician, client, and system variables on the implementation of ET. Evidence was classified into most of the TDF domains, however a subset of domains (i.e., *knowledge*, *social/professional role and identity*, *beliefs about consequences*, and *environmental context and resources*) accounted for the majority of research to date. Targeting domains relating to knowledge and beliefs makes intuitive sense, and interventions that have addressed these domains have produced results indicating moderate improvements in ET use (Trivasse et al., 2020). Most notably, through the provision of training (Frank et al., 2020). However, *knowledge* and *beliefs about consequences* are only two of the 14 TDF domains. The domains of *optimism*, *reinforcement*, and *memory, attention, and decision processes* are unexplored in relation to ET use and warrant consideration given their theoretical relevance to healthcare providers implementation behaviours (Cane et al., 2012; Michie et al., 2005). In addition, within domains there were typically a range of variables explored. This meant that most constructs were examined by one or two findings. Due to this low volume of evidence, there was limited ability to draw meaningful conclusions across many constructs. These small bodies of evidence could be attributed to an unintuitive relationship between the constructs (e.g., clients’ relationship status) and ET use, or the under exploration of seemingly relevant variables (e.g., primary diagnosis, session length). It is vital future research equally dedicate resources to examining all relevant variables across the full spectrum of domains, including capturing practical issues identified by clinicians delivering or attempting to implement ET in routine care. Nevertheless, there were some domains and constructs that were examined across several results that allowed conclusions to be drawn across client presentations and developmental subgroups, and are discussed, as relevant, below.

#### The Determinants of ET Implementation Across Presentations and Developmental Subgroups

Some domains and constructs demonstrated consistent findings across presentations, whilst others varied. An example of the former was clinician beliefs about ET which consistently related to its use in treating clients across all presentations. Specifically, clinicians who held positive beliefs about the consequences of ET, including its benefits and risks, were more likely to use it in their practice. These relationships have been suggested in primary studies and in commentaries on the issue (Blakey et al., 2018; Olatunji et al., 2009) which has likely resulted in the disproportionately high focus on this domain. In contrast, whilst knowledge, in the form of unspecified ET training, related to ET use for anxiety disorders, this was not apparent for OCD or PTSD. However, unspecified training in imaginal and prolonged exposure was positively related to ET use for PTSD. This suggests that whilst general ET training can encourage uptake for clients with anxiety disorders that are treated using foundational ET techniques, for more complex presentations like OCD and PTSD, additional specialised training covering disorder-specific techniques may be required to promote the use of ET. Furthermore, contextual factors including the clinician’s exposure to specific anxiety presentations and practical (e.g., role-plays, simulated cases) disorder-specific training, particularly in the context of PTSD, were positively associated with the use of ET. Whilst a small number of findings suggested that clinician age, as well as client age and female gender are negatively related to ET use in specific presentations or developmental subgroups, more exploration is required to determine if these relationships are direct or are products of extraneous variables like the therapeutic drift throughout a career (Waller & Turner, 2016) or the level of risk clinician’s perceive when using ET for certain demographics based on their assumptions around that demographics’ resiliency (e.g., Whiteside et al., 2016a, 2016b). The presence of such differences in the consistency of determinants of ET implementation across client presentations only emphasises the need for future research to treat these as truly distinct implementation contexts.

When considering developmental subgroups, similar trends emerged with a few key nuanced differences. For instance, unique negative beliefs about the impact of ET on youth appeared to be related to less use of ET for the treatment of this demographic. Findings suggested this may be compounded by concerns on the resiliency of anxious children. In contrast, for adults, especially in military or veteran populations, factors such as working within PTSD specialty clinics and experience with anxiety or PTSD presentations were positively related to ET use. Apart from these differences, the scarcity of research exploring specific developmental subgroups, especially youth has hampered the ability to distinguish important trends between these groups. Across the lifespan, beliefs about ET and its consequences, as well as specific training in ET were key factors that support its application. These patterns suggest that targeted interventions (e.g., practical disorder-specific training) to improve clinician knowledge, beliefs, and skills could increase the adoption of ET across presentations and developmental subgroups. Overall, it is vital the literature more evenly dedicate resources to exploring ET implementation across developmental subgroups, whilst respecting their uniqueness.

#### Underexplored Determinants of ET Implementation Across the TDF

The results of this review have highlighted several areas for future research. Foremost, it has identified several domains on the TDF where future research should focus to improve our understanding of the determinants of ET implementation. Such research should consider focusing on the determinants where there is conflicting evidence or a paucity of evidence to clarify relationships, and explore determinants theoretically linked to utilisation in implementation theories. For instance, the role of reinforcement (including incentives and other contingencies) has not been explored in relation to ET implementation, where doing so may maximise the benefits of this interventions. For example, understanding the relationship between making access to government rebates contingent upon providing evidence-based practices, including potential moderators (e.g., clinicians’ sentiments; Rieckmann et al., 2011), will ultimately help maximise any improvements in uptake. Whilst the relationship between ET use and reinforcement seems relatively intuitive, the roles of other underexplored determinants such as optimism, behavioural regulation, and memory, attention, and decision processes are equally important. For example, the proliferation of findings linking beliefs about ET as an important determinant of its implementation throughout the literature suggests that a clinician’s attitudes are central to their implementation behaviour. This suggests that their general disposition toward viewing the anticipated outcome of events either positively or negatively (i.e., optimism; Cane et al., 2012) likely has important implications for their implementation behaviour. Exploring the potential role of such determinants is important, not only because it may help identify areas for direct intervention, for example by improving optimism (Malouff & Schutte, 2017), but may encourage indirect intervention in other areas like clinician burnout (Bell et al., 2024). Such examples, highlight the need for thorough and considered investigations into factors that influence clinicians’ ET implementation behaviour.

### Implications for Improving the Implementation of ET in Practice

Given the patterns and differences across presentations and developmental subgroups outlined above, there are broad practical implications for improving ET uptake. Indeed, implementation determinants identified using the TDF can inform targeted interventions (Atkins et al., 2017) using the BCW, through their shared foundation (Michie et al., 2014)—the underlying COM-B model of behaviour (Michie et al., 2011). The COM-B model conceptualises volitional behaviour as generated by an interaction between an individual’s capability, opportunity, and motivation, which are in turn influenced by behaviour. Alongside the BCW, the TDF provides an empirically supported framework to assess determinants of ET use, that will enable future researchers to select, tailor, and implement interventions to improve it—important steps in translating knowledge into action (Graham et al., 2006) and improving the uptake of ET in the treatment of anxiety-related presentations. Given the prevalence of anxiety-related presentations (Kessler et al., 2012), clinicians’ opportunities to use ET are high, and the results from this review suggest that those clinicians more frequently exposed to relevant presentations in practice are indeed more likely to use ET. Additional steps can be taken, for example the reduction of practical barriers to ET may be warranted by structuring the social and physical environment within mental health services to facilitate the opportunity to practice ET in routine care, such as ensuring appropriate spaces and time are provided to conduct ET and clinicians have ready access to the necessary resources and support. Furthermore, service provision changes, such as incorporating ET as a standard component of care for anxiety-related presentations, can ensure that clinicians have the opportunity within their organisation to apply ET.

As suggested above, improving clinicians’ capabilities and motivation to use ET is perhaps the more pressing issue. Our results demonstrate that providing specific and practical education (e.g., simulation-based learning experiences), that is tailored to different presentations of anxiety disorders, OCD, PTSD, and other anxiety-related presentations (Harned et al., 2014; Sherrill et al., 2021) is necessary. This is vital as unspecified ET training dominates the literature and is associated with only medium-sized improvements in the use of ET (Trivasse et al., 2020). Additionally, training must target more than knowledge and skills, for example by targeting clinician’s confidence in applying ET or addressing misinformation on the benefits and, especially, the risks of ET. Such approaches should be integrated within university training programmes and as part of continuing professional development for mental health clinicians, including within large mental health systems, where clinicians commonly receive training (Sars & van Minnen, 2015). It is also likely that combining interventions, such as training and supervision, could produce additional gains in ET use, over a single intervention. For example, a systematic review of the impact of training on clinicians’ knowledge, beliefs, and behaviours by Frank and colleagues (2020) indicated that although training is helpful for improving knowledge and beliefs toward evidence-based practices, they rarely lead to increase implementation alone, but are more likely to if accompanied by consultation. Combining the benefits of interventions is important given that many single interventions, such as those aimed at improving motivation to use ET (e.g., motivational interviewing calls), may not influence ET use on their own. Addressing motivation is important because improving clinicians’ capabilities and opportunities to implement ET may not be sufficient if motivation is low. Additionally, evidence suggests that self-monitoring of current practices relative to evidence-based guidelines does not predict greater ET use (Becker-Haimes et al., 2017); thus the role of clinician motivation, both directly and indirectly, warrants further investigation. To address motivation considering interventions such as regulation and legislation may be warranted given that clinicians who are less likely to use ET contradictorily report a higher willingness to use evidence-based practices if they were required to (Becker-Haimes et al., 2017). Overall, by translating determinants of ET use from the literature into theoretically informed behaviour change interventions within healthcare systems, governments, and training providers can, through implementation science, create targeted, comprehensive, and evidence-based strategies that are more likely to drive sustained behaviour change in clinicians. This will ultimately encourage better treatment outcomes for clients in need of evidence-based treatment, in the form of ET.

### Limitations and Directions for Future Research

The current review possesses several limitations common in reviews. There is a possibility that relevant studies were missed (Gartlehner et al., 2020) but this was minimised by undertaking a comprehensive search of seven databases and Google Scholar, alongside using Connected Papers to search for related records. We limited the search to studies published in the English language, as is common (Jackson & Kuriyama, 2019), which likely limited research from different cultural settings. As recommended by systematic review guidelines (Page et al., 2021), the methodological quality of the included studies was evaluated, and the MMAT was selected to account for a range of study designs. Some study designs did not neatly fit those prescribed in the MMAT (e.g., Whiteside et al., 2023) and were categorised based on their dominant features. This may have resulted in the quality appraisal missing important aspects of the designs of some included studies. MMAT scores were arbitrarily classified into very poor, poor, moderate, good, and excellent, to concisely describe the methodological quality of a single and a group of studies. This approach, of tallying scores for individual items to provide a summary score, is variably described in the literature (Tod et al., 2022). Other quality appraisal tools could have been used and these may have provided a different overview of methodological quality or risk of bias of the included studies (Katrak et al., 2004). Additionally, limitations associated with the evidence must also be considered. For example, some studies explored a multitude of ET variants which were consolidated for the purposes of addressing our review questions. Whilst this assisted to gain an understanding of the relationship of the extracted determinants with overall ET implementation, future research should distinguish the determinants for specific ET delivery forms (e.g., in vivo, imaginal, in-session, homework). Finally, throughout the literature the use of self-reported ET implementation measures was also problematic, as was the self-report measure of other constructs, specifically under skills where role-plays or clinical observation would significantly improve the validity of findings. The use of self-reported ET implementation measures also meant the relationships identified may not adequately account for the influence of shared decision-making, a critical component of best-practice psychological intervention (Patel et al., 2008). Whilst it is widely accepted that clinicians drive the underutilisation of ET (Jaeger et al., 2010), it is nevertheless important to further understand the contribution of client influences, perhaps using qualitative or mixed-methods.

Beyond improving the coverage of theoretical implementation determinants in the literature, this review has also elucidated several other areas for future research. One of the primary issues in the literature is that the construct of interest being explored as a potential determinant of implementation behaviour must be clearly defined using established behaviour change taxonomies from implementation science (e.g., the Behaviour Change Technique Taxonomy; Michie et al., 2013). This may require the development of new, psychometrically sound measures to identify and differentiate a range of determinants. In addition to this, we also found that many studies do not specify the client presentations and age ranges that were sampled nor were subgroup analyses conducted. Exploring and analysing ET use in a range of specific presentations and developmental subgroups will assist to determine how ET use can be maximised in the different contexts where the research-practice gap is present.

### Conclusions

Based on existing evidence from 52 studies and nearly 400 results, it appears clinicians’ negative beliefs about the consequences of ET, including its benefits and risks, are related to reduced ET use. This is especially problematic in youth, where unique beliefs about its risks, or the capabilities of youth to tolerate ET were associated with less use. Whilst knowledge, including via unspecified ET training, is positively related to ET use for some presentations (i.e., anxiety disorders), this is inconsistent between presentations and suggests that training specific to complex presentations (i.e., PTSD) and involving practical components (e.g., observational learning, supervision, and competency-based assessment of skills) is more likely to improve ET use. Overall, the research undertaken to date was able to be classified into the TDF domains and the extent to which each domain was investigated varied significantly. Optimism, reinforcement, and memory, attention, and decision processes are currently unexplored in the context of ET implementation, and there is a paucity of research for constructs aligned to behavioural regulation. Research has focused on specific presentations and developmental subgroups (i.e., PTSD and adults) whilst others were under-researched (i.e., OCD and youth). Future research should utilise established scientific methods for evaluating and improving implementation to explore a range of determinants, clarify differences between presentations and developmental subgroups, and efficiently translate knowledge of the drivers of ET underuse into integrative multi-level interventions.

## Other Information

### Registration and Protocol

Minor amendments were made to the protocol prospectively registered on PROSPERO (CRD42022308100) throughout the study to maximise the quality of the synthesis produced. Given the breadth of available literature, eligibility criteria were refined to exclude lower quality designs (e.g., case studies, N of 1), qualitative designs, descriptive results (Daly et al., 2007; OCEBM Levels of Evidence Working Group & OCEBM Levels of Evidence Working Group, 2011), less relevant samples (e.g., students not completing practical clinical training), ET variants not operationalised within CBT, and studies that solely explored the use of ET workbooks or resources. Given the dominance of cross-sectional studies the review questions were altered from identifying domains as barriers and facilitators to only determining whether domains were related to ET use. Given a lack of literature exploring ET implementation in preschoolers, children, and adolescents, these developmental subgroups were collapsed into youth.

## Supplementary Information

Below is the link to the electronic supplementary material.

Online Resource 1 (PDF 121 kb)

Online Resource 2 (PDF 276 kb)

Online Resource 3 (PDF 169 kb)

Online Resource 4 (PDF 128 kb)

Online Resource 5 (PDF 182 kb)

Online Resource 6 (PDF 457 kb)

## Data Availability

Supplementary material, such as data extraction and quality appraisal templates can be requested from the corresponding author using the contact details provided.
