# Correction: Determinants of Exposure Therapy Implementation in Clinical Practice for the Treatment of Anxiety, OCD, and PTSD: A Systematic Review

**DOI:** 10.1007/s10567-024-00496-1

**Published:** 2024-09-11

**Authors:** J. I. Racz, A. Bialocerkowski, I. Calteaux, L. J. Farrell

**Affiliations:** 1 School of Applied Psychology, Grifth University, Southport, QLD Australia; 2 Grifth Health, Grifth University, Southport, QLD Australia

**Correction to: Clinical Child and Family Psychology Review (2024) 27:317–341** 10.1007/s10567-024-00478-3

The original version of this article unfortunately contained errors in Fig. 1.

The article was published with components of Figure 1 missing from the diagram. The original and complete Fig. 1 is provided below.

**Fig. 1:**
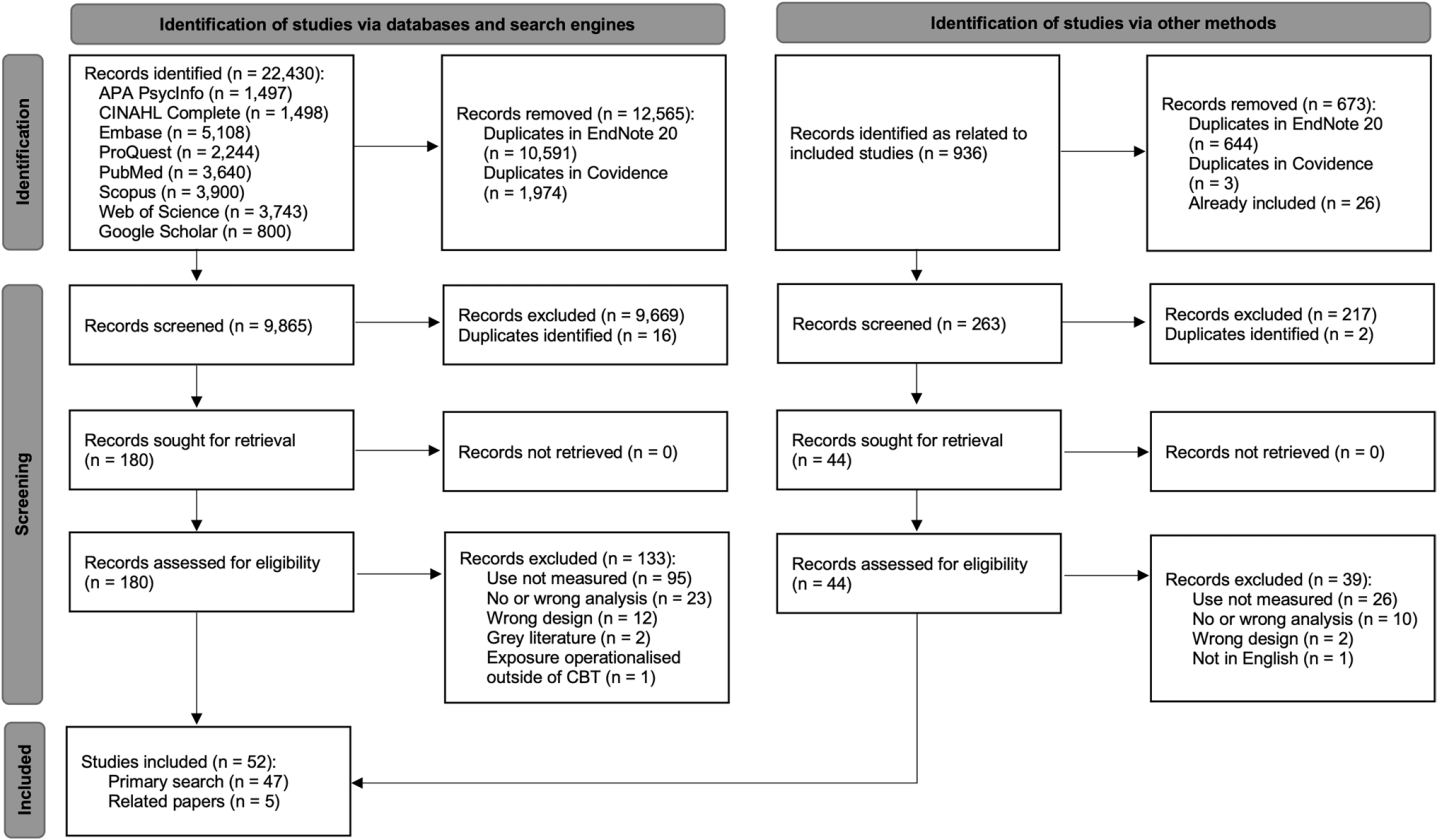
Preferred Reporting Items for Systematic Reviews and Meta-Analyses (PRISMA) Flow Diagram (Page et al., 2021)

The original article has been corrected.

