# Supplementary material for: Determinants of Exposure Therapy Implementation in Clinical Practice for the Treatment of Anxiety, OCD, and PTSD: A Systematic Review
Source: Clin Child Fam Psychol Rev. 2024 Apr 17;27(2):317–41. doi: 10.1007/s10567-024-00478-3 (PMC11222222; doi:10.1007/s10567-024-00478-3)
Supplement: Supplementary file 1 — Online Resource 1 (PDF 121 kb) [file 10567_2024_478_MOESM1_ESM.pdf]

Online Resource 1

Search Translation Summary

APA PsycInfo (Ovid)

Search Terms

(adher* OR administ* OR adopt* OR barrier* OR compl* OR deliver* OR diffus* OR disseminat* OR enabl* OR facilitat* OR imped* OR implement* OR integrat* OR know-do OR knowledge to action OR obstacle* OR research-practice OR translat* OR uptak* OR utili*) OR (exp information dissemination/) AND (desensiti*ation OR exposure and response prevention OR exposure-based OR exposure response prevention OR exposure session OR exposure task OR exposure therapy OR exposure therapies OR exposure treatment OR exposure with response prevention OR flooding OR graded exposure OR imaginal exposure OR implosive therapy OR interoceptive exposure OR in vivo exposure OR prolonged exposure) OR (exp exposure therapy/) AND (anxiety OR obsessive-compulsive OR post-traumatic stress OR stress and trauma OR trauma and stress) OR (exp anxiety disorders/ OR exp stress and trauma related disorders/)

Search Field

Keyword (Default)

Limits

- Peer-reviewed journal
- English language

CINAHL Complete (EBSCO)

Search Terms

(adher* OR administ* OR adopt* OR barrier* OR compl* OR deliver* OR diffus* OR disseminat* OR enabl* OR facilitat* OR imped* OR implement* OR integrat* OR “know-do” OR “knowledge to action” OR obstacle* OR “research-practice” OR translat* OR uptak* OR utili*) OR (MH “diffusion of innovation+” OR MH “translational medical research”) AND (desensiti*ation OR “exposure * response prevention” OR “exposure-based” OR “exposure session” OR “exposure task” OR “exposure therapy” OR “exposure therapies” OR “exposure treatment” OR flooding OR “graded exposure” OR “imaginal exposure” OR “implosive therapy” OR “interoceptive exposure” OR “in vivo exposure” OR “prolonged exposure”) OR (MH “desensitization, psychologic+”) AND

(anxiety OR “obsessive-compulsive” OR “post-traumatic stress” OR “stress and trauma” OR “trauma and stress”) OR (MH “anxiety disorders+”)

Search Field

Default

Limits

- English language
- Peer reviewed

Embase

Search Terms

(adher* OR administ* OR adopt* OR barrier* OR compl* OR deliver* OR diffus* OR disseminat* OR enabl* OR facilitat* OR imped* OR implement* OR integrat* OR ‘know-do’ OR ‘knowledge to action’ OR obstacle* OR ‘research-practice’ OR translat* OR uptak* OR utili*) OR (‘diffusion of innovation’/syn OR ‘implementation science’/syn OR ‘translational research’/syn) AND (desensiti*ation OR ‘exposure and response prevention’ OR ‘exposure-based’ OR ‘exposure response prevention’ OR ‘exposure session’ OR ‘exposure task’ OR ‘exposure therapy’ OR ‘exposure therapies’ OR ‘exposure treatment’ OR ‘exposure with response prevention’ OR flooding OR ‘graded exposure’ OR ‘imaginal exposure’ OR ‘implosive therapy’ OR ‘interoceptive exposure’ OR ‘in vivo exposure’ OR ‘prolonged exposure’) OR (‘exposure therapy’/syn) AND (anxiety OR ‘obsessive-compulsive’ OR ‘post-traumatic stress’ OR ‘stress and trauma’ OR ‘trauma and stress’) OR (‘anxiety disorder’/syn)

Search Field

Default

Limits

- Language: English

ProQuest

Databases Searched

- International Bibliography of the Social Sciences
- ProQuest Central
    - Australian & New Zealand Database

- Consumer Health Database
- Continental Europe Database
- East & South Asia Database
- East Europe, Central Europe Database
- Education Database
- Health & Medical Collection
- Healthcare Administration Database
- India Database
- Latin America & Iberia Database
- Middle East & Africa Database
- Military Database
- Nursing & Allied Health Database
- Psychology Database
- Public Health Database
- Publicly Available Content Database
- Research Library
- Science Database
- Social Science Database
- Turkey Database
- UK & Ireland Database

- PTSDpubs

Search Terms

(adher* OR administ* OR adopt* OR barrier* OR compl* OR deliver* OR diffus* OR disseminat* OR enabl* OR facilitat* OR imped* OR implement* OR integrat* OR “know-do” OR “knowledge to action” OR obstacle* OR “research-practice” OR translat* OR uptak* OR utili*) AND (desensiti*ation OR “exposure and response prevention” OR “exposure-based” OR “exposure response prevention” OR “exposure session” OR “exposure task” OR “exposure therapy” OR “exposure therapies” OR “exposure treatment” OR “exposure with response prevention” OR flooding OR “graded exposure” OR “imaginal exposure” OR “implosive therapy” OR

“interoceptive exposure” OR “in vivo exposure” OR “prolonged exposure”) AND (anxiety OR “obsessive-compulsive” OR “post-traumatic stress” OR “stress and trauma” OR “trauma and stress”)

Search Field

Anywhere except full text – NOFT

Limits

- Peer reviewed
- Language: English

PubMed

Search Terms

(adher* OR administ* OR adopt* OR barrier* OR compl* OR deliver* OR diffus* OR disseminat* OR enabl* OR facilitat* OR imped* OR implement* OR integrat* OR “know-do” OR “knowledge to action” OR obstacle* OR “research-practice” OR translat* OR uptak* OR utili*) OR (diffusion of innovation [mh] OR translational medical research [mh]) AND (desensitisation OR desensitization OR “exposure and response prevention” OR “exposure-based” OR “exposure response prevention” OR “exposure session” OR “exposure task” OR “exposure therapy” OR “exposure therapies” OR “exposure treatment” OR “exposure with response prevention” OR flooding OR “graded exposure” OR “imaginal exposure” OR “implosive therapy” OR “interoceptive exposure” OR “in vivo exposure” OR “prolonged exposure”) OR (implosive therapy [mh] OR virtual reality exposure therapy [mh]) AND (anxiety OR “obsessive-compulsive” OR “post-traumatic stress” OR “stress and trauma” OR “trauma and stress”) OR (anxiety disorders [mh] OR trauma and stressor related disorders [mh])

Search Field

All fields (Default)

Limits

- Language: English

Scopus

Search Terms

(adher* OR administ* OR adopt* OR barrier* OR compl* OR deliver* OR diffus* OR disseminat* OR enabl* OR facilitat* OR imped* OR implement* OR integrat* OR {know-do} OR {knowledge to action} OR

obstacle* OR {research-practice} OR {research practice} OR translat* OR uptak* OR utili*) AND (desensiti*ation OR {exposure and response prevention} OR {exposure-based} OR {exposure based} OR {exposure response prevention} OR {exposure session} OR {exposure task} OR {exposure therapy} OR {exposure therapies} OR {exposure treatment} OR {exposure with response prevention} OR flooding OR {graded exposure} OR {imaginal exposure} OR {implosive therapy} OR {interoceptive exposure} OR {in vivo exposure} OR {prolonged exposure}) AND (anxiety OR {obsessive-compulsive} OR {obsessive compulsive} OR {post-traumatic stress} OR {post traumatic stress} OR {stress and trauma} OR {trauma and stress})

Search Field

Article title, Abstract, Keywords (Default)

Limits

- Language: English

Web of Science

Databases

- Web of Science Core Collection

Search Terms

(adher* OR administ* OR adopt* OR barrier* OR compl* OR deliver* OR diffus* OR disseminat* OR enabl* OR facilitat* OR imped* OR implement* OR integrat* OR "know-do" OR "knowledge to action" OR obstacle* OR "research-practice" OR translat* OR uptak* OR utili*) AND (desensiti*ation OR "exposure and response prevention" OR "exposure-based" OR "exposure response prevention" OR "exposure session" OR "exposure task" OR "exposure therapy" OR "exposure therapies" OR "exposure treatment" OR "exposure with response prevention" OR flooding OR "graded exposure" OR "imaginal exposure" OR "implosive therapy" OR "interoceptive exposure" OR "in vivo exposure" OR "prolonged exposure") AND (anxiety OR "obsessive-compulsive" OR "post-traumatic stress" OR "stress and trauma" OR "trauma and stress")

Search Field

All fields (Default)

Limits

- Languages: English

Google Scholar

Pages Searched

- Up to and including the 80th page of results (800 results).

Search Terms

(disseminate OR implement OR utilise OR deliver OR administer) AND ("exposure therapy" OR "exposure task" OR "exposure and response prevention" OR "exposure with response prevention" OR “prolonged exposure” OR “interoceptive exposure”)

Limits

None
