# Supplementary material for: Determinants of Exposure Therapy Implementation in Clinical Practice for the Treatment of Anxiety, OCD, and PTSD: A Systematic Review
Source: Clin Child Fam Psychol Rev. 2024 Apr 17;27(2):317–41. doi: 10.1007/s10567-024-00478-3 (PMC11222222; doi:10.1007/s10567-024-00478-3)
Supplement: Supplementary file 2 — Online Resource 2 (PDF 276 kb) [file 10567_2024_478_MOESM2_ESM.pdf]

Online Resource 2

Details of Included Studies

Table 1

Details of Included Studies

Study	Design	Method	Country	Participants	Presentation	Developmental Subgroup	Therapy	Aim	Sample Details
(C. B. Becker et al., 2004)	Quantitative descriptive	Survey	United States	207 psychologists and 29 members of a trauma special interest group	PTSD	Lifespan (unspecified)	Imaginal exposure	To assess licensed psychologists use of imaginal exposure for PTSD and investigate the perceived barriers to its implementation.	Unknown age and gender. 100% psychologists.
(S. Becker et al., 2018)	Quantitative descriptive	Survey	Germany	209 trainee and licensed cognitive-behavioural therapists with experiencing treating panic disorder and/or agoraphobia	Anxiety disorders (panic disorder and/or agoraphobia)	Lifespan (unspecified)	Exposure therapy (general)	To determine the psychometric properties of a German version of the TBES and obtain preliminary indications of its construct validity.	M_{age} = 35 years (*SD* = 8). 82% female. 96% studied psychology at university, 2% pedagogy, 1% medicine, 1% social pedagogy, 1% teaching, and 1% political and social sciences.
(Becker-Haimes et al., 2020)	Quantitative descriptive	Survey	United States	24 leaders of specialty anxiety clinics and 19 community clinicians	Anxiety-related presentations (unspecified)	Lifespan (unspecified)	Exposure therapy (general)	To identify the innovation-specific organisational capacity necessary to deliver exposure therapy.	Clinic leaders: M_{age} = 48 years (range 32 – 69), 50% female, and 75% clinical psychologists. Community clinicians: M_{age} = 43, 79% female.

Study	Design	Method	Country	Participants	Presentation	Developmental Subgroup	Therapy	Aim	Sample Details
									Unknown occupations.
(Becker-Haimes et al., 2017)	Quantitative descriptive	Survey	United States	335 community mental health clinicians	Anxiety-related presentations (combined anxiety disorders, OCD, and PTSD)	Youth (unspecified)	Exposure therapy (general)	To characterise clinician and organisational predictors of exposure use and relaxation for anxiety.	M_{age} = 39 years (*SD* = 12). 78% female. Unknown occupations.
(Chen et al., 2022)	Quantitative descriptive	Survey	Australia	60 registered psychologists	Anxiety-related presentations (unspecified)	Adults (unspecified)	Exposure, cognitive, anxiety management, and other techniques	To examine the use of exposure therapy compared to other treatment techniques among clinicians who treat older adults with anxiety disorders.	M_{age} = 44 (*SD* = 14). 83% female.
(Cook, Dinnen, Coyne, et al., 2015)	Mixed methods	Sequential	United States	198 mental health providers from Department of Veterans Affairs' residential treatment programs	PTSD	Adults (unspecified)	Prolonged exposure and cognitive processing therapy	To evaluate factors influencing provider use of two evidence-based treatments for PTSD.	Unknown age. 64% female. 56% psychologists, 33% social workers, 6% nurses, 3% psychiatrists, and 3% other.
(Cook, Dinnen, Thompson, et al., 2015)	Quantitative descriptive	Survey	United States	201 PTSD treatment providers in Veterans Affairs residential treatment settings	PTSD	Adults (unspecified)	Prolonged exposure and cognitive processing therapy	To examine the implementation of two evidence-based psychotherapies, Prolonged Exposure and Cognitive Processing Therapy, in the Department of	Unknown age and gender. 51% doctoral-level psychologists.

Study	Design	Method	Country	Participants	Presentation	Developmental Subgroup	Therapy	Aim	Sample Details
								Veterans Affairs residential PTSD treatment programs.	
(Cook et al., 2014)	Mixed methods	Convergence	United States	190 Veterans Affairs residential PTSD treatment program providers	PTSD	Adults (unspecified)	Prolonged exposure and cognitive processing therapy	To determine the status of training, implementation of prolonged exposure and cognitive processing therapy, and its predictors in VA residential PTSD treatment programs.	M_{age} = 46 (*SD* = 11). 60% female. 63% psychologists, 31% social workers, 4% nurses, and 3% psychiatrists.
(Cook et al., 2013)	Mixed methods	Sequential	United States	267 directors, providers (sub-sample of 179), and staff members from 38 sites in the Veteran's Integrated Service Networks	PTSD	Adults (unspecified)	Prolonged exposure and cognitive processing therapy	To report on the initial adoption of prolonged exposure and cognitive processing therapy in these settings during the initial stages of the system-wide national Veterans Affairs dissemination initiative to train providers in these treatments for PTSD.	Unknown age and gender. 41% psychologists, 21% social workers, 14% nurses, 7% psychiatrists, and 17% other.
(Cook, Simiola, et al., 2020)	Mixed methods	Sequential	United States	526 providers from 39 Veteran Affairs residential PTSD treatment programs	PTSD	Adults (unspecified)	Prolonged exposure and cognitive processing therapy	To examine the patterns of adoption of prolonged exposure and cognitive processing therapy	Unknown age and gender. The most frequent profession (of unknown proportion) was psychologist,

Study	Design	Method	Country	Participants	Presentation	Developmental Subgroup	Therapy	Aim	Sample Details
								in Department of Veterans Affairs residential PTSD treatment programs.	followed by social worker, nurse, and other (e.g., psychiatrist, chaplain).
(Cook, Thompson, & Schnurr, 2015)	Quantitative descriptive	Survey	United States	215 Department of Veterans Affairs residential treatment providers	PTSD	Adults (unspecified)	Prolonged exposure and cognitive processing therapy	To examine the psychometric properties and factor structure of the Perceived Characteristics of Intervention Scale (PCIS), developed to capture provider perceptions of a particular mental health care intervention.	M_{age} = 45 (*SD* = 11). 62% female. 52% psychologists, 30% social workers, and 19% psychiatrists, nurses, or other.
(Cook, Thompson, et al., 2020)	Quantitative descriptive	Survey	United States	159 providers from 38 Department of Veterans Affairs residential PTSD programs	PTSD	Adults (unspecified)	Prolonged exposure and cognitive processing therapy	To examine the role of attitudes toward evidence-based psychotherapies in predicting use of prolonged exposure and cognitive processing therapy for PTSD among treatment providers within the Department of Veterans Affairs.	Unknown age and gender. 44% psychologists, 38% social workers, 8% psychiatrists, and 11% other.

Study	Design	Method	Country	Participants	Presentation	Developmental Subgroup	Therapy	Aim	Sample Details
(Deacon et al., 2013) [a]	Quantitative non-randomised	Repeated measures	United States	162 therapists	Anxiety-related presentations (unspecified)	Lifespan (unspecified)	Exposure therapy (general)	To examine the reliability and validity of the Therapist Beliefs about Exposure Scale (TBES) in three samples of practicing clinicians.	M_{age} = 51 (*SD* = 13). 76% female. Trainers reported most attendees were masters-level providers in the fields of social work or counselling.
(de Jong et al., 2020)	Quantitative descriptive	Survey	Netherlands and Belgium	207 youth mental health care professionals	Anxiety-related presentations (unspecified)	Youth (unspecified)	Exposure therapy (general)	To examine what therapist characteristics may be involved in the (non-)use of exposure in treating childhood anxiety disorders.	Most common age range was 30 to 39 years old (38%). 93% female. 34% health care psychologist, 29% psychologist without specialization, 13% social worker not specialized, all other professions less than 13%.
(Finley et al., 2015)	Quantitative descriptive	Survey	United States	128 non-prescribing providers from Veteran Affairs PTSD clinics	PTSD	Adults (unspecified)	Prolonged exposure and cognitive processing therapy	To describe the utilisation of prolonged exposure and cognitive processing therapy and to identify individual and organisational factors associated with uptake and adherence.	M_{age} = 44 (*SD* = 11). 70% female. 54% doctorate in clinical psychology, 33% masters in social work with advanced clinical certification, 12% doctorate in counselling psychology, and 2% masters in social work.

Study	Design	Method	Country	Participants	Presentation	Developmental Subgroup	Therapy	Aim	Sample Details
(Foa et al., 2020)	Quantitative randomised controlled trial	-	United States	242 clients (active military personnel with PTSD symptoms) and 103 active duty or civilian behavioral health providers at three US Army medical treatment facilities	PTSD	Adults (unspecified)	Prolonged exposure	To determine the utility of an implementation strategy and to assess clinical outcomes associated with the strategy.	Clients: Unknown age. 84% male. Providers: Unknown age. 63% female. 61% social workers, 34% psychologists, 1% mental health counsellors, and 4% other.
(Garcia et al., 2020)	Quantitative descriptive	Survey	United States	227 psychologists and social workers working with a PTSD clinical team at the Veterans Health Administration	PTSD	Adults (unspecified)	Cognitive processing therapy, prolonged exposure, and other techniques	To describe use of CPT, PE, and “other” psychotherapies among Veterans Health Administration PTSD specialty care providers, and examine factors associaed with providers' use of treatments.	The largest age group (37%) was 30-39 years. 69% female. 67% psychologists and 33% social workers.
(Harned et al., 2013)	Quantitative randomised controlled trial	-	United States	181 therapists naive to exposure therapy	Anxiety-related presentations (unspecified)	Lifespan (unspecified)	Exposure therapy (general)	To examine organisational, client, and therapist characteristics as predictors of use of and proficiency in exposure therapy after training.	M_{age} = 37 (*SD* = 10). 71% female. 29% masters level counsellors, 28% social workers, 23% student trainee, 14% psychologists, 4% psychiatrists, 2% psychiatric nurses, and 2%

Study	Design	Method	Country	Participants	Presentation	Developmental Subgroup	Therapy	Aim	Sample Details
									bachelors level counsellors.
(Harned et al., 2014)	Quantitative randomised controlled trial	-	United States	181 mental health professionals actively treating anxiety disorder patients	Anxiety-related presentations (combined anxiety disorders and PTSD)	Lifespan (unspecified)	Exposure therapy (general)	To evaluate three technology-based methods of training mental health providers in exposure therapy for anxiety disorders.	*M*$_{age}$ = 37 (*SD* = 10). 71% female. 29% masters level counsellors, 28% social workers, 23% student trainee, 14% psychologists, 4% psychiatrists, 2% psychiatric nurses, and 2% bachelors level counsellors.
(Harned et al., 2011)	Quantitative randomised controlled trial	-	United States	46 treatment providers at a mental health agency or students working to obtain a professional degree in a related field	Anxiety-related presentations (unspecified)	Lifespan (unspecified)	Exposure therapy (general)	To evaluate methods for training mental health providers in exposure therapies for anxiety disorders.	*M*$_{age}$ = 41 (*SD* = 11). 83% female. 26% social workers, 26% masters level counsellors, 24% psychologists, 4% bachelors level counsellors, 4% registered nurse or advanced registered nurse practitioner, and 15% other.
(Harned et al., 2021)	Quantitative non-randomised	Repeated measures	United States and United Kingdom	266 participants from five continuing education workshops focused on providing training in the dialectical	PTSD	Lifespan (unspecified)	Dialectical behaviour therapy prolonged exposure	To examine the impact of dialectical behaviour therapy prolonged exposure workshops on clinician beliefs, adoption, and	Unknown age. 83% cisgender women, 17% cisgender men, and 0.4% transgender. 85% licensed professionals, 9% postgraduate

Study	Design	Method	Country	Participants	Presentation	Developmental Subgroup	Therapy	Aim	Sample Details
				behaviour therapy prolonged exposure protocol				perceived patient outcomes.	trainee, and 5% graduate-level trainee.
(Hertz et al., 2023)	Quantitative non-randomised	Cross-sectional	United States	Records of 554 veterans newly diagnosed with OCD in the Veterans Health Administration	OCD	Adults (unspecified)	Exposure and response prevention	To examine the rates of ERP delivery in a national sample of veterans newly diagnosed with OCD in the Veterans Health Administration between 2016 and 2017.	Veterans: M_{age} = 51 (*SD* = 16). 79% male. Clinicians: ERP sessions (for 22 veterans) were mostly conducted by psychologists (68%), 23% by psychology trainees, and 9% by social workers. Unknown age and gender.
(Hipol & Deacon, 2013)	Quantitative descriptive	Survey	United States	51 licensed and practising psychotherapists from various professions in Wyoming	Anxiety-related presentations (combined anxiety disorders, OCD, and PTSD)	Lifespan (unspecified)	Cognitive-behavioural therapy techniques including exposure therapy (general)	To examine the utilisation of psychotherapy techniques, especially cognitive-behavioural therapy, for anxiety disorders among community practitioners in a rural setting.	M_{age} = 54 (*SD* = 11). 61% female. 29% psychologists, 27% licensed counsellors, 25% clinical social workers, 12% psychotherapists, 4% psychiatrists, and 2% licensed marriage and family counsellors.
(Kannis-Dymand et al., 2022)	Quantitative descriptive	Survey	Australia and New Zealand	164 registered and provisionally registered psychologists	Anxiety disorders	Lifespan (16 years or older)	Interoceptive exposure	To examine whether psychologists often avoid undertaking interoceptive	Unknown age. 82% female.

Study	Design	Method	Country	Participants	Presentation	Developmental Subgroup	Therapy	Aim	Sample Details
								exposure in Australia and New Zealand.	
(Keleher et al., 2020)	Quantitative descriptive	Survey	United Kingdom	107 mental health clinicians	OCD	Youth (unspecified)	Exposure and response prevention	To examine the utilisation of exposure and response prevention for paediatric obsessive-compulsive disorder via an anonymous online survey completed by clinicians.	Unknown age and gender. 55% clinical psychologists, 13% nurses, 8% psychiatrists, 5% trainee clinical psychologists, 5% social workers, 4% psychotherapists, and 10% others.
(Klan et al., 2017)	Quantitative non-randomised	Cohort	Germany	Records of 92 patients (with a main diagnosis agoraphobia) in an outpatient setting treated by 63 CBT therapists	Anxiety disorders (agoraphobia)	Adults (unspecified)	In vivo exposure	To analyse potential predictors of the application of exposure in vivo in the treatment of agoraphobia.	Patients: M_{age} = 36 (*SD* = 12). 66% female. Therapists: Unknown age and gender. 90% sessions conducted by pre-licensed CBT therapists in training under supervision and 10% by licensed CBT therapists.
(Kline et al., 2021)	Quantitative descriptive	Survey	United States	155 licensed mental health professionals	PTSD	Adults (unspecified)	Treatments for PTSD (e.g., prolonged exposure, cognitive processing therapy, eye movement desensitisation	To survey a range of licensed clinicians to evaluate the use of interventions to treat adults with PTSD and identify therapist characteristics associated with	M_{age} = 40 (*SD* = 11). 68% female. Unknown occupation.

Study	Design	Method	Country	Participants	Presentation	Developmental Subgroup	Therapy	Aim	Sample Details
							and reprocessing)	exposure training, beliefs, and utilization likelihood.	
(Maguen et al., 2019)	Quantitative non-randomised	Cross-sectional	United States	Records of 265,566 Iraq and Afghanistan war veterans with a post-deployment PTSD diagnosis at one of 130 Veterans Health Administration facilities	PTSD	Adults (unspecified)	Prolonged exposure and cognitive processing therapy	To examine patient and treatment factors associated with initiation and completion of EBP for PTSD in a large longitudinal cohort.	M_{age} = 32 – 34 (*SD* = 8 – 9). 89% male.
(Meyer et al., 2020)	Quantitative descriptive	Survey	Australia	98 psychologists	Anxiety-related presentations (unspecified)	Lifespan (unspecified)	Exposure therapy (general)	To identify the beliefs exposure therapists have regarding the necessity of therapist safety behaviours and examine their relationship with therapist safety behaviour use.	M_{age} = 37 (*SD* = 11). 81% female.
(Moritz et al., 2019)	Quantitative descriptive	Survey	Germany	216 therapists	OCD	Lifespan (unspecified)	Exposure and response prevention	To determine the extent to which exposure and response prevention is conducted for OCD among therapists along with their reasons for avoiding the approach.	M_{age} = 47 (*SD* = 10). 67% female. 84% psychologists, 12% physicians, and 4% others.
(Moses et al., 2022)	Mixed methods	Sequential	Australia	100 psychologists	Anxiety-related presentations	Lifespan (unspecified)	Exposure, CBT, third-wave CBT, and	To examine barriers to the use of	M_{age} = 41 (*SD* = 11). 84% female.

Study	Design	Method	Country	Participants	Presentation	Developmental Subgroup	Therapy	Aim	Sample Details
					(combined anxiety disorders, OCD, and PTSD), anxiety disorders, OCD, and PTSD		other techniques	exposure therapy in the clinical practice of psychologists treating anxiety, OCD and PTSD.	
(Moses et al., 2021)	Quantitative descriptive	Survey	Australia	100 psychologists working in clinical practice	Anxiety-related presentations (combined anxiety disorders, OCD, and PTSD), anxiety disorders, OCD, and PTSD	Lifespan (unspecified)	Exposure therapy (general), exposure and response prevention, interoceptive exposure	To examine the use and predictors of use of exposure therapy in psychologists who treat clients with an anxiety disorder, OCD, or PTSD.	Unknown age. 84% female.
(Parker & Waller, 2019)	Quantitative descriptive	Survey	United Kingdom	173 therapists	Anxiety-related presentations (unspecified)	Lifespan (unspecified)	Exposure therapy (general)	To assess therapists reported use of cognitive-behavioural techniques in the treatment of anxiety disorders, and which therapist variables influence this use.	M_{age} = 45 (*SD* = 11). 68% female. 25% clinical psychologists, 18% psychiatric nurses, 12% CBT therapists, 8% counselling psychologists, 5% licensed professional counsellors, 3% clinical social workers, 1% psychiatrists, 25% other healthcare professionals, and 3% unreported.

Study	Design	Method	Country	Participants	Presentation	Developmental Subgroup	Therapy	Aim	Sample Details
(Pittig et al., 2019)	Quantitative descriptive	Survey	Germany	684 licensed behavioural psychotherapists working in outpatient routine care	Anxiety-related presentations (combined anxiety disorders, OCD, and PTSD)	Lifespan (unspecified)	Exposure therapy (general), imaginal exposure, interoceptive exposure	To examine barriers to the dissemination of exposure from the perspective of behavioural psychotherapists.	M_{age} = 46 (*SD* = 9). 79% female.
(Reid et al., 2017)	Quantitative descriptive	Survey	United States	230 practising clinicians	Anxiety disorders, OCD, and PTSD	Youth (7 to 17 years)	Therapist-assisted in vivo exposure	To explore the prevalence of common barriers to the use of exposure therapy in the treatment of youth with anxiety disorders.	M_{age} = 49 (*SD* = 13). 72% female. 46% psychologists, 30% clinical social workers, 18% master level counsellors, 1% nurse practitioners, and 4% other mental health related professions.
(Reid et al., 2018)	Quantitative descriptive	Survey	United States	257 private practice therapists	Anxiety disorders, OCD, and PTSD	Youth (7 to 17 years)	Non-exposure CBT techniques, exposure techniques, third-wave CBT techniques, and other	To identify how practicing clinicians treat youth with anxiety disorders and what factors contribute to their use of exposure therapy.	M_{age} = 49 (*SD* = 12). 75% female. 42% psychologists, 31% clinical social workers, 20% master level counsellors, 3% nurse practitioners, 1% marriage and family therapists, and 3% other.
(Rosen et al., 2019)	Quantitative non-randomised	Cohort	United States	Records of 6,251 veterans receiving psychotherapy over one year in PTSD specialty	PTSD	Adults (unspecified)	Cognitive processing therapy and prolonged exposure	To identify clinical and demographics factors associated with initiating either cognitive	M_{age} = 48 – 52 (*SD* = 14 – 15). 77 – 84% male.

Study	Design	Method	Country	Participants	Presentation	Developmental Subgroup	Therapy	Aim	Sample Details
				clinics at nine Veterans Affairs medical centres				processing therapy or prolonged exposure, combined and separately.	
(Rosen et al., 2017)	Quantitative descriptive	Survey	United States	566 licensed Veterans Health Administration mental health clinicians treating one or more veterans with PTSD	PTSD	Adults (unspecified)	Prolonged exposure	To examine aspects of clinicians' work environment that facilitate sustained use of prolonged exposure therapy.	Unknown age. 65% female. 57% psychologists, 38% social workers, and 5% others.
(Rowe & Kangas, 2020)	Quantitative descriptive	Survey	Australia	115 psychologists	Anxiety-related presentations (combined anxiety disorders and OCD)	Adults (unspecified)	First- and second-wave CBT techniques, motivational interviewing, non-directive counselling, and other techniques	To investigate the relative contributions of different factors in relation to the delivery of exposure therapy by psychologists.	M_{age} = 42 (*SD* =11). 82% female.
(Ruzek et al., 2017)	Quantitative non-randomised	Repeated measures	United States	1,034 licensed Veterans Health Administration mental health clinicians (734 at six-months follow-up)	PTSD	Adults (unspecified)	Prolonged exposure	To examine how changes in beliefs during an intensive training process predict adoption of prolonged exposure therapy.	Unknown age. 67% female. 59% psychologists, 36% social workers, and 6% others.
(Sars & van Minnen, 2015)	Quantitative descriptive	Survey	Netherlands	490 members (i.e., psychologists, clinical psychologists, and supervisors) of the Dutch Association for	Anxiety disorders and OCD	Lifespan (unspecified)	Exposure therapy (general) and other cognitive techniques	To examine the use of exposure therapy among members of a Dutch CBT association, as well as explore therapist,	M_{age} = 46 (*SD* = 11). 75% female. 31% were therapists in training (i.e., psychologists receiving training in CBT), 39%

Study	Design	Method	Country	Participants	Presentation	Developmental Subgroup	Therapy	Aim	Sample Details
				Behavioural and Cognitive Therapists				educational and contextual variables that could facilitate its dissemination in clinical practice.	were certified therapists (i.e., clinical psychologists licensed as CBT therapists), and 30% were supervisors (therapists providing training in CBT).
(Schumacher et al., 2018)	Quantitative descriptive	Survey	Germany	331 cognitive behavioural therapists	Anxiety disorders and PTSD	Lifespan (unspecified)	Exposure therapy (general)	To examine the dissemination and application frequency of exposure among cognitive behavioural therapists working in routine care.	M_{age} = 43 (*SD* = 10). 73% female. 82% psychological psychotherapists, 9% child and youth psychotherapists, and 5% more than one qualification.
(Sherrill et al., 2021)	Quantitative non-randomised	Repeated measures	United States	40 prolonged exposure consultants, 85 of their trainees, and 102 patients of the trainees	PTSD	Lifespan (unspecified)	Prolonged exposure	To evaluate the effectiveness of a training program aimed at developing a national network of community-based prolonged exposure consultants.	Consultants: Unknown age. 68% female. 68% PhD psychologists, 15% PsyD psychologists, 13% social workers, 5% mental health counsellors, and 3% psychiatrists. Trainees: Unknown age. 71% female. 33% PhD psychologists, 29% social

Study	Design	Method	Country	Participants	Presentation	Developmental Subgroup	Therapy	Aim	Sample Details
									workers, 26% mental health counsellors, 9% PhD psychology students, and 1% psychiatrists. Patients: Unknown age and gender.
(van Minnen et al., 2010)	Quantitative randomised controlled trial	-	Unspecified (likely Netherlands)	255 trauma experts	PTSD	Lifespan (unspecified)	Imaginal exposure, eye movement reprocessing and desensitisation, and supportive counselling	To investigate when and why therapists opt for or rule out imaginal exposure for patients with PTSD.	M_{age} = 49 (*SD* = 10). 65% female. 46% psychologists or psychotherapists, 15% social workers or social psychiatric nurses, 13% psychiatrists or physicians, 27% other (e.g., researcher or policy maker).
(Vande Voort et al., 2010) [b]	Quantitative non-randomised	Cross-sectional	United States	43 child patients seen in an outpatient clinic and four treating therapists	Anxiety-related presentations (combined anxiety disorders and OCD)	Youth (6 to 18 years)	Exposure therapy, relaxation, anxiety management, and behaviour management	To examine the content of clinic-based treatment for childhood anxiety disorders.	Patients: M_{age} = 12 (*SD* = 3). 51% female. Therapists: Unknown age and gender. A licensed doctoral-level clinical psychologist, a postdoctoral clinical psychology fellow, and two master level psychologists.

Study	Design	Method	Country	Participants	Presentation	Developmental Subgroup	Therapy	Aim	Sample Details
(Wade et al., 2020)	Quantitative non-randomised	Repeated measures	Australia	45 psychologists	PTSD	Lifespan (unspecified)	Prolonged exposure	To evaluate the efficacy of a prolonged exposure training workshop plus intensive consultation programme.	Unknown age. 81% female.
(Whiteside, Biggs, Dammann, et al., 2022)	Quantitative non-randomised	Repeated measures	United States	24 therapists working in the community	Anxiety-related presentations (combined anxiety disorders and OCD)	Youth (7 to 17 years)	Exposure therapy (general) and other therapy techniques	To examine community-therapist response to a brief (90-minutes) training in technology-assisted exposure therapy for childhood anxiety disorders.	Unknown age. 96% female. 63% social workers, 17% licensed marriage and family therapists, 13% licensed professional counsellors, 8% doctoral level psychologists.
(Whiteside, Biggs, Ollendick, et al., 2022) [b]	Quantitative randomised controlled trial	-	United States	17 therapists and 32 youth and a parent presenting for outpatient treatment for an anxiety disorder	Anxiety-related presentations (combined anxiety disorders and OCD)	Youth (8 to 18 years)	Exposure therapy (in vivo, imaginal, and interoceptive)	To investigate whether brief training in the delivery of an exposure-focused and technology-assisted treatment protocol increased community therapist openness to exposure therapy, use of exposure in treatment, and improvement in patient symptoms.	Youth: M_{age} = 12 (*SD* = 3). 78% female. Therapists: Unknown age. 94% female. 71% social workers, 18% masters-level therapists, and 12% doctoral-level psychologists.
(Whiteside, Deacon, et al., 2016)	Quantitative descriptive	Survey	United States	331 therapists from a variety of backgrounds	Anxiety-related presentations (combined anxiety	Youth (unspecified)	Exposure therapy (general), anxiety management	To examine factors related to the use of exposure therapy by clinicians who	Unknown age and gender. 43% social workers, 30% doctoral level

Study	Design	Method	Country	Participants	Presentation	Developmental Subgroup	Therapy	Aim	Sample Details
					disorders, OCD, and PTSD)		strategies, CBT-related techniques, and non-CBT techniques	treat children with anxiety disorders.	psychologists, 16% masters degree licensed counsellors, 11% marriage and family therapists.
(Whiteside, Sattler, et al., 2016)	Quantitative non-randomised	Cross-sectional	United States	Records of 482 treatment sessions received by 86 youth at a medical centre by 39 clinicians	Anxiety-related presentations (combined anxiety disorders, OCD, and PTSD)	Youth (7 to 17 years)	Cognitive-behavioural therapy, exposure therapy (general), non-CBT, and medication management	To examine the clinical documentation for 482 treatment sessions received by 86 youth, aged 7 to 17, with an anxiety disorder.	Youth: M_{age} = 13 (SD = 4). 54% female. Clinicians: Unknown age and gender. 50% of sessions conducted by masters level therapists and licensed clinical social workers, 31% by doctoral-level psychologists, 16% by psychiatrists and psychiatric nurse practitioners, and 4% by non-mental health providers (e.g., primary care providers).
(Whiteside et al., 2023)	Quantitative randomised controlled trial	-	United States	25 youth with an anxiety disorder and 13 therapists working in a large regional health system	Anxiety disorders (e.g., generalised anxiety disorder, specific phobia, social anxiety disorder,	Youth (9 to 17 years)	Exposure therapy (general), anxiety management strategies, general therapy, and other	To advance efforts to increase the quality of care for childhood anxiety disorders through better understanding the treatment delivered in	Youth: M_{age} = 12 (SD = 2). 80% female. Therapists: Unknown age. 100% female. 77% social workers, 15% masters level psychologists,

Study	Design	Method	Country	Participants	Presentation	Developmental Subgroup	Therapy	Aim	Sample Details
					separation anxiety disorder)			community settings.	and 8% doctoral-level psychologists.
(Živčić-Bećirević et al., 2019)	Quantitative descriptive	Survey	Croatia	226 psychotherapists	Anxiety-related presentations (unspecified)	Lifespan (unspecified)	Exposure therapy (general)	To examine therapists' attitudes toward exposure therapy and differences in beliefs according to type and level of psychotherapeutic education as well as experience with using this type of therapy.	M_{age} = 35 (*SD* = 8). 87% female. 79% psychologists, 13% psychiatrists and psychiatry specialist trainees, and 8% others.

Note.
[a] This article reported on three separate but related studies. Only the results of the third study were relevant to this review and were extracted and appraised.
[b] These studies were defined as relevant to youth, not lifespan, due to the predominant age-range of the sample.
