# Supplementary material for: Determinants of Exposure Therapy Implementation in Clinical Practice for the Treatment of Anxiety, OCD, and PTSD: A Systematic Review
Source: Clin Child Fam Psychol Rev. 2024 Apr 17;27(2):317–41. doi: 10.1007/s10567-024-00478-3 (PMC11222222; doi:10.1007/s10567-024-00478-3)
Supplement: Supplementary file 3 — Online Resource 3 (PDF 169 kb) [file 10567_2024_478_MOESM3_ESM.pdf]

Online Resource 3

Quality Appraisal Using the MMAT

Table 1

Quality Appraisal Using the MMAT (2018)

Study	Screening and Design-Specific Criteria							Quality Score
	Research Questions	Appropriate Data	Appropriate Randomisation	Comparable Groups	Complete Outcome Data	Blinded Assessors	Intervention Adherence	
	Quantitative Randomised Controlled							
(Foa et al., 2020)	Y	Y	Y	Y	N	C	N	40%
(Harned et al., 2013)	Y	Y	Y	C	Y	C	C	40%
(Harned et al., 2014)	Y	Y	Y	Y	Y	C	Y	80%
(Harned et al., 2011)	Y	Y	Y	Y	Y	C	Y	80%
(van Minnen et al., 2010)	Y	Y	C	Y	Y	N	Y	60%
(Whiteside, Biggs, Ollendick, et al., 2022)	Y	Y	N	Y	Y	Y	Y	80%
(Whiteside et al., 2023)	Y	Y	C	N	N	Y	Y	40%
	Quantitative Non-Randomised							
	Research Questions	Appropriate Data	Representative Sample	Appropriate Measures	Complete Outcome Data	Confounds Addressed	Intervention Administered	
(Deacon et al., 2013) [a]	Y	Y	Y	Y	Y	N	Y	80%
(Harned et al., 2021)	Y	Y	Y	Y	N	N	Y	60%
(Hertz et al., 2023)	Y	Y	Y	Y	Y	N	Y	80%
(Klan et al., 2017)	Y	Y	N	Y	C	Y	Y	60%
(Maguen et al., 2019)	Y	Y	Y	Y	Y	Y	Y	100%
(Rosen et al., 2019)	Y	Y	N	Y	Y	Y	Y	80%

(Ruzek et al., 2017)	Y	Y	C	N	Y	Y	Y	60%
(Sherrill et al., 2021)	Y	Y	Y	Y	N	N	Y	60%
(Vande Voort et al., 2010)	Y	Y	N	Y	C	N	Y	40%
(Wade et al., 2020)	Y	Y	C	N	Y	Y	Y	60%
(Whiteside, Biggs, Dammann, et al., 2022)	Y	Y	C	Y	C	Y	Y	60%
(Whiteside, Sattler, et al., 2016)	Y	Y	N	N	Y	N	Y	40%
Quantitative Descriptive								
	Research Questions	Appropriate Data	Appropriate Sampling	Representative Sample	Appropriate Measures	Low Response Bias	Appropriate Analysis	
(C. B. Becker et al., 2004)	Y	Y	Y	N	N	N	N	20%
(S. Becker et al., 2018)	Y	Y	Y	Y	C	C	Y	60%
(Becker-Haimes et al., 2020)	Y	Y	N	Y	Y	Y	Y	80%
(Becker-Haimes et al., 2017)	Y	Y	Y	Y	Y	C	Y	80%
(Chen et al., 2022)	Y	Y	Y	Y	Y	C	Y	80%
(Cook, Dinnen, Thompson, et al., 2015)	Y	Y	Y	Y	Y	Y	Y	100%
(Cook, Thompson, et al., 2015)	Y	Y	Y	N	N	Y	Y	60%
(Cook, Thompson, et al., 2020)	Y	Y	Y	N	N	C	Y	40%
(de Jong et al., 2020)	Y	Y	N	N	Y	N	Y	40%
(Finley et al., 2015)	Y	Y	Y	Y	N	N	Y	60%
(Garcia et al., 2020)	Y	Y	Y	Y	Y	N	Y	80%
(Hipol & Deacon, 2013)	Y	Y	Y	Y	Y	N	Y	80%
(Kannis-Dymand et al., 2022)	Y	Y	Y	Y	Y	N	Y	80%
(Keleher et al., 2020)	Y	Y	Y	Y	Y	C	Y	80%
(Kline et al., 2021)	Y	Y	N	N	N	C	Y	20%

(Meyer et al., 2020)	Y	Y	N	Y	Y	N	Y	60%
(Moritz et al., 2019)	Y	Y	Y	Y	N	N	Y	60%
(Moses et al., 2021)	Y	Y	Y	Y	N	C	Y	60%
(Parker & Waller, 2019)	Y	Y	C	N	Y	N	Y	40%
(Pittig et al., 2019)	Y	Y	Y	Y	N	N	Y	60%
(Rosen et al., 2017)	Y	Y	Y	Y	N	N	Y	60%
(Reid et al., 2017)	Y	Y	C	Y	Y	N	Y	60%
(Reid et al., 2018)	Y	Y	Y	Y	Y	N	Y	80%
(Rowe & Kangas, 2020)	Y	Y	N	N	Y	N	Y	40%
(Sars & van Minnen, 2015)	Y	Y	C	N	N	N	Y	20%
(Schumacher et al., 2018)	Y	Y	Y	Y	N	N	N	40%
(Whiteside, Deacon, et al., 2016)	Y	Y	Y	Y	Y	N	Y	80%
(Živčić-Bećirević et al., 2019)	Y	Y	C	N	C	C	Y	20%

Mixed Methods

	Research Questions	Appropriate Data	Methods Rationale	Effective Integration	Adequate Interpretation	Divergences Addressed	Quality Components	
(Cook, Dinnen, Coyne, et al., 2015)	Y	Y	N	N	N	N	N	0%
(Cook et al., 2014)	Y	Y	C	C	N	N	N	0%
(Cook et al., 2013)	Y	Y	N	N	N	Y	N	20%
(Cook, Simiola, et al., 2020)	Y	Y	Y	Y	Y	C	C	60%
(Moses et al., 2022)	Y	Y	Y	Y	N	C	N	40%

Note. Y = Yes; N = No; C = Can't tell. The five core design-specific criteria were used to calculate a percentage indicative of overall quality, which was categorised as very low (i.e., ≤ 20%), low (i.e., 40%), moderate (i.e., 60%), high (i.e., 80%), and very high (i.e., 100%).

[a] This article reported on three separate but related studies. Only the results of the third study were relevant to this review and were extracted and appraised.
