# Supplementary material for: Determinants of Exposure Therapy Implementation in Clinical Practice for the Treatment of Anxiety, OCD, and PTSD: A Systematic Review
Source: Clin Child Fam Psychol Rev. 2024 Apr 17;27(2):317–41. doi: 10.1007/s10567-024-00478-3 (PMC11222222; doi:10.1007/s10567-024-00478-3)
Supplement: Supplementary file 4 — Online Resource 4 (PDF 128 kb) [file 10567_2024_478_MOESM4_ESM.pdf]

Online Resource 4

Frequency of Results Exploring Each Domain of the TDF Categorised by Presentation and Developmental Subgroup

Table 1

Frequency of Results Exploring Each Domain of the TDF Categorised by Presentation and Developmental Subgroup

Category	TDF Domains														Unclassified Results
	K	S	SPRI	BACA	O	BACO	R	I	G	MAD	ECR	SI	E	BR	
	Overall Number of Results (Percentage of Mapped Results)														
	48 (12%)	22 (6%)	87 (22%)	17 (4%)	0 (0%)	59 (15%)	0 (0%)	7 (2%)	13 (3%)	0 (0%)	82 (21%)	22 (6%)	30 (8%)	2 (1%)	5
	Number of Results (with Percentage of Results for that Domain) for Each Presentation														
Anxiety Disorders	10 (21%)	3 (14%)	8 (9%)	1 (6%)	0	10 (17%)	0	1 (14%)	1 (8%)	0	6 (7%)	2 (9%)	12 (40%)	0 (0%)	1
OCD	9 (19%)	4 (18%)	14 (16%)	1 (6%)	0	4 (7%)	0	1 (14%)	1 (8%)	0	3 (4%)	2 (9%)	4 (13%)	0 (0%)	1
PTSD	15 (31%)	8 (36%)	53 (61%)	10 (59%)	0	25 (42%)	0	2 (29%)	4 (31%)	0	49 (60%)	9 (41%)	3 (10%)	0 (0%)	3
Anxiety-Related Presentations	14 (29%)	7 (32%)	12 (14%)	5 (29%)	0	20 (34%)	0	3 (43%)	7 (54%)	0	24 (29%)	9 (41%)	11 (37%)	2 (100%)	0
	Number of Results (with Percentage of Results for that Domain) for Each Developmental Subgroup														
Youth	6 (13%)	6 (27%)	16 (18%)	1 (6%)	0	10 (17%)	0	1 (14%)	5 (38%)	0	13 (16%)	2 (9%)	12 (40%)	2 (100%)	3
Adults	9 (19%)	2 (9%)	55 (63%)	7 (41%)	0	22 (37%)	0	2 (29%)	4 (31%)	0	49 (60%)	13 (59%)	8 (27%)	0 (0%)	2
Lifespan	33 (69%)	14 (64%)	16 (18%)	9 (53%)	0	27 (46%)	0	4 (57%)	4 (31%)	0	20 (24%)	7 (32%)	10 (33%)	0 (0%)	0

Note. Mapped results totalled 389 with 5 additional results unclassifiable. K = Knowledge. S = Skills. SPRI = Social/Professional Role and Identity. BACA = Beliefs about Capabilities. O = Optimism. BACO = Beliefs about Consequences. R = Reinforcement. I = Intentions. G = Goals. MAD = Memory, Attention, and Decision Processes. ECR = Environmental Context and Resources. SI = Social Influences. E = Emotion. BR = Behavioural Regulation. Lifespan is defined as unspecified developmental subgroups or a combination of youth and adult developmental subgroups. Anxiety-related presentations are defined as an unspecified or specified a combination of anxiety disorders, OCD, and PTSD.
