# Supplementary material for: Determinants of Exposure Therapy Implementation in Clinical Practice for the Treatment of Anxiety, OCD, and PTSD: A Systematic Review
Source: Clin Child Fam Psychol Rev. 2024 Apr 17;27(2):317–41. doi: 10.1007/s10567-024-00478-3 (PMC11222222; doi:10.1007/s10567-024-00478-3)
Supplement: Supplementary file 5 — Online Resource 5 (PDF 182 kb) [file 10567_2024_478_MOESM5_ESM.pdf]

Online Resource 5

Domains on the TDF Explored by the Individual Studies

Table 1

Domains on the TDF Explored by the Individual Studies

Study	TDF Domains														Unclassified
	K	S	SPRI	BACA	O	BACO	R	I	G	MAD	ECR	SI	E	BR	
(C. B. Becker et al., 2004)	✓	✓													
(S. Becker et al., 2018)		✓				✓									
(Becker-Haimes et al., 2020)											✓				
(Becker-Haimes et al., 2017)	✓	✓						✓	✓		✓	✓	✓	✓	
(Chen et al., 2022)			✓			✓									
(Cook, Dinnen, Coyne, et al., 2015)						✓					✓	✓			
(Cook, Dinnen, Thompson, et al., 2015)			✓			✓		✓			✓	✓			
(Cook et al., 2014)	✓		✓												✓
(Cook et al., 2013)	✓														
(Cook, Simiola, et al., 2020)											✓	✓			
(Cook, Thompson, & Schnurr, 2015)						✓									
(Cook, Thompson, et al., 2020)			✓			✓			✓		✓				✓
(Deacon et al., 2013)						✓									
(de Jong et al., 2020)		✓	✓			✓					✓		✓		
(Finley et al., 2015)		✓	✓			✓					✓	✓			
(Foa et al., 2020)												✓			
(Garcia et al., 2020)	✓		✓												
(Harned et al., 2013)	✓	✓	✓	✓		✓		✓			✓	✓	✓		
(Harned et al., 2014)		✓										✓			

Study	TDF Domains														Unclassified
	K	S	SPRI	BACA	O	BACO	R	I	G	MAD	ECR	SI	E	BR	
(Harned et al., 2011)		✓						✓							
(Harned et al., 2021)		✓		✓		✓									
(Hertz et al., 2023)			✓												
(Hipol & Deacon, 2013)			✓												
(Kannis-Dymand et al., 2022)	✓					✓									
(Keleher et al., 2020)		✓	✓			✓						✓			
(Klan et al., 2017)			✓			✓					✓	✓	✓		
(Kline et al., 2021)	✓														
(Maguen et al., 2019)			✓								✓				
(Meyer et al., 2020)						✓			✓						
(Moritz et al., 2019)			✓												
(Moses et al., 2022)	✓			✓		✓					✓	✓	✓		
(Moses et al., 2021)		✓	✓								✓				
(Parker & Waller, 2019)	✓		✓	✓		✓					✓	✓	✓		
(Pittig et al., 2019)				✓		✓					✓		✓		
(Reid et al., 2017)															✓
(Reid et al., 2018)	✓		✓			✓							✓		
(Rosen et al., 2019)			✓								✓				
(Rosen et al., 2017)			✓	✓		✓					✓				
(Rowe & Kangas, 2020)	✓		✓			✓						✓			
(Ruzek et al., 2017)		✓	✓	✓		✓			✓		✓				
(Sars & van Minnen, 2015)	✓	✓				✓		✓	✓		✓		✓		
(Schumacher et al., 2018)		✓	✓			✓									
(Sherrill et al., 2021)		✓													
(van Minnen et al., 2010)	✓		✓			✓									
(Vande Voort et al., 2010)									✓		✓		✓		
(Wade et al., 2020)	✓														

Study	TDF Domains														Unclassified
	K	S	SPRI	BACA	O	BACO	R	I	G	MAD	ECR	SI	E	BR	
(Whiteside, Biggs, Dammann, et al., 2022)	✓														
(Whiteside, Biggs, Ollendick, et al., 2022)		✓							✓						
(Whiteside, Deacon, et al., 2016)		✓	✓	✓		✓									
(Whiteside, Sattler, et al., 2016)											✓				
(Whiteside et al., 2023)											✓				
(Živčić-Bećirević et al., 2019)						✓									
Number of Studies (% of Total) Exploring Each Domain	16 (31%)	17 (33%)	24 (46%)	8 (15%)	0 (0%)	27 (52%)	0 (0%)	5 (10%)	7 (13%)	0 (0%)	22 (42%)	13 (25%)	10 (19%)	1 (2%)	3 (6%)

Note. Mapped results totalled 389 with 5 additional results unclassifiable. K = Knowledge. S = Skills. SPRI = Social/Professional Role and Identity. BACA = Beliefs about Capabilities. O = Optimism. BACO = Beliefs about Consequences. R = Reinforcement. I = Intentions. G = Goals. MAD = Memory, Attention, and Decision Processes. ECR = Environmental Context and Resources. SI = Social Influences. E = Emotion. BR = Behavioural Regulation.
