# Supplementary material for: Determinants of Exposure Therapy Implementation in Clinical Practice for the Treatment of Anxiety, OCD, and PTSD: A Systematic Review
Source: Clin Child Fam Psychol Rev. 2024 Apr 17;27(2):317–41. doi: 10.1007/s10567-024-00478-3 (PMC11222222; doi:10.1007/s10567-024-00478-3)
Supplement: Supplementary file 6 — Online Resource 6 (PDF 457 kb) [file 10567_2024_478_MOESM6_ESM.pdf]

Online Resource 6

Summary of Results of Included Studies

Table 1

Summary of Results of Included Studies

TDF Domains	Study	Construct Focus	Constructs	Notes	Relationship to ET Use	Presentation	Developmental Subgroup
Knowledge	(C. B. Becker et al., 2004)	Clinician	Engagement in imaginal exposure training.	-	Positively related	PTSD	Lifespan
	(C. B. Becker et al., 2004)	Clinician	Engagement in imaginal exposure training.	-	Positively related	PTSD	Lifespan
	(Becker-Haimes et al., 2017)	Clinician	Knowledge of exposure as an evidence-based practice.	-	Unrelated	Anxiety-related presentations	Youth
	(Becker-Haimes et al., 2017)	Clinician	Training in exposure therapy.	-	Unrelated	Anxiety-related presentations	Youth
	(Cook et al., 2014)	Clinician	Level of training in prolonged exposure.	-	Positively related	PTSD	Adults
	(Cook et al., 2013)	Clinician	Level of training in prolonged exposure.	-	Positively related	PTSD	Adults
	(Garcia et al., 2020)	Clinician	Certified through the national prolonged exposure training initiative.	-	Positively related	PTSD	Adults
	(Garcia et al., 2020)	Clinician	Received CBT training in graduate school.	-	Unrelated	PTSD	Adults
	(Harned et al., 2013)	Clinician	Knowledge of exposure therapy and ability to apply it.	-	Positively related [a]	Anxiety-related presentations	Lifespan

TDF Domains	Study	Construct Focus	Constructs	Notes	Relationship to ET Use	Presentation	Developmental Subgroup
	(Kannis-Dymand et al., 2022)	Clinician	Receipt of specific training in exposure therapy (e.g., at university, reading, supervision).	-	Positively related	Anxiety disorders	Lifespan
	(Kline et al., 2021)	Clinician	Training in exposure therapy.	-	Positively related	PTSD	Adults
	(Moses et al., 2022)	Clinician	Training on exposure therapy within postgraduate education.	-	Unrelated	Anxiety-related presentations	Lifespan
	(Moses et al., 2022)	Clinician	Training on exposure therapy within postgraduate education.	Unrelated to the use of exposure therapy for anxiety and interoceptive exposure for panic disorder.	Unrelated	Anxiety disorders	Lifespan
	(Moses et al., 2022)	Clinician	Training on exposure therapy within postgraduate education.	-	Unrelated	OCD	Lifespan
	(Moses et al., 2022)	Clinician	Training on exposure therapy within postgraduate education.	-	Unrelated	PTSD	Lifespan
	(Moses et al., 2022)	Clinician	Training on exposure therapy via professional workshop or conference attendance.	-	Unrelated	Anxiety-related presentations	Lifespan
	(Moses et al., 2022)	Clinician	Training on exposure therapy via professional workshop or conference attendance.	Positively related to the use of exposure therapy for anxiety and interoceptive exposure for panic disorder.	Positively related	Anxiety disorders	Lifespan
	(Moses et al., 2022)	Clinician	Training on exposure therapy via professional workshop or conference attendance.	-	Unrelated	OCD	Lifespan
	(Moses et al., 2022)	Clinician	Training on exposure therapy via professional workshop or conference attendance.	-	Positively related	PTSD	Lifespan

TDF Domains	Study	Construct Focus	Constructs	Notes	Relationship to ET Use	Presentation	Developmental Subgroup
	(Moses et al., 2022)	Clinician	Training on exposure therapy via supervision with an experienced professional.	-	Unrelated	Anxiety-related presentations	Lifespan
	(Moses et al., 2022)	Clinician	Training on exposure therapy via supervision with an experienced professional.	Unrelated to the use of exposure therapy for anxiety and interoceptive exposure for panic disorder.	Unrelated	Anxiety disorders	Lifespan
	(Moses et al., 2022)	Clinician	Training on exposure therapy via supervision with an experienced professional.	-	Unrelated	OCD	Lifespan
	(Moses et al., 2022)	Clinician	Training on exposure therapy via supervision with an experienced professional.	-	Unrelated	PTSD	Lifespan
	(Moses et al., 2022)	Clinician	Training on exposure therapy within a specialist anxiety clinic.	-	Unrelated	Anxiety-related presentations	Lifespan
	(Moses et al., 2022)	Clinician	Training on exposure therapy within a specialist anxiety clinic.	Positively related to the use of exposure therapy for anxiety and interoceptive exposure for panic disorder.	Positively related	Anxiety disorders	Lifespan
	(Moses et al., 2022)	Clinician	Training on exposure therapy within a specialist anxiety clinic.	-	Unrelated	OCD	Lifespan
	(Moses et al., 2022)	Clinician	Training on exposure therapy within a specialist anxiety clinic.	-	Positively related	PTSD	Lifespan
	(Moses et al., 2022)	Clinician	Knowledge about exposure therapy.	-	Positively related [a]	Anxiety-related presentations	Lifespan
	(Moses et al., 2022)	Clinician	Knowledge about exposure therapy.	Positively related to the use of exposure therapy for anxiety and interoceptive exposure for panic disorder.	Positively related	Anxiety disorders	Lifespan

TDF Domains	Study	Construct Focus	Constructs	Notes	Relationship to ET Use	Presentation	Developmental Subgroup
	(Moses et al., 2022)	Clinician	Knowledge about exposure therapy.	-	Positively related	OCD	Lifespan
	(Moses et al., 2022)	Clinician	Knowledge about exposure therapy.	-	Positively related	PTSD	Lifespan
	(Parker & Waller, 2019)	Clinician	Additional post-qualification accreditation.	-	Unrelated	Anxiety-related presentations	Lifespan
	(Reid et al., 2018)	Clinician	Training in exposure-based techniques during and/or after graduate school.	-	Positively related	Anxiety disorders	Youth
	(Reid et al., 2018)	Clinician	Training in exposure-based techniques during and/or after graduate school.	-	Positively related	OCD	Youth
	(Reid et al., 2018)	Clinician	Training in exposure-based techniques during and/or after graduate school.	-	Unrelated	PTSD	Youth
	(Rowe & Kangas, 2020)	Clinician	Understanding of the mechanisms of change underlying exposure, according to the habituation model.	Negatively related to frequency of use. Unrelated to in-session duration of use.	Negatively related	Anxiety-related presentations	Adults
	(Rowe & Kangas, 2020)	Clinician	Understanding of the mechanisms of change underlying exposure, according to the cognitive model.	Positively related to frequency of use. Unrelated to in-session duration of use.	Positively related	Anxiety-related presentations	Adults
	(Rowe & Kangas, 2020)	Clinician	Understanding of the mechanisms of change underlying exposure, according to the inhibitory learning model.	Positively related to frequency and in-session duration of use.	Positively related	Anxiety-related presentations	Adults
	(Rowe & Kangas, 2020)	Clinician	Extent to which training received to date has covered exposure therapy.	Unrelated to frequency and in-session duration of use.	Unrelated	Anxiety-related presentations	Adults
	(Sars & van Minnen, 2015)	Clinician	Extent of exposure training in practical skills. Measured and analysed separately for each of the presentations (i.e., social anxiety, specific phobia, and panic disorder).	Positively related to use of therapist-directed in vivo exposure, in vivo self-exposure, and exposure-based homework assignments for social anxiety. Unrelated to use of	Positively related	Anxiety disorders	Lifespan

TDF Domains	Study	Construct Focus	Constructs	Notes	Relationship to ET Use	Presentation	Developmental Subgroup
				imaginal exposure, exposure and response prevention and interoceptive exposure for social anxiety.<br><br>Positively related to use of therapist-directed in vivo exposure, in vivo self-exposure, and exposure-based homework assignments for specific phobia. Unrelated to use of imaginal exposure, exposure and response prevention and interoceptive exposure for specific phobia.<br><br>Positively related to use of therapist-directed in vivo exposure, in vivo self-exposure, interoceptive exposure, and exposure-based homework assignments for panic disorder. Unrelated to use of imaginal exposure and exposure and response prevention for panic disorder.			
	(Sars & van Minnen, 2015)	Clinician	Extent of exposure training in practical skills for OCD.	Positively related to use of therapist-directed in vivo exposure, in vivo self-exposure, exposure and response prevention, and exposure-based homework assignments. Unrelated to use of imaginal exposure and interoceptive exposure.	Positively related	OCD	Lifespan

TDF Domains	Study	Construct Focus	Constructs	Notes	Relationship to ET Use	Presentation	Developmental Subgroup
	(Sars & van Minnen, 2015)	Clinician	Extent of exposure training in diagnostic skills. Measured and analysed separately for each of the presentations (i.e., social anxiety, specific phobia, and panic disorder).	Positively related to use of therapist-directed in vivo exposure, in vivo self-exposure, and exposure-based homework assignments for social anxiety. Unrelated to use of imaginal exposure, exposure and response prevention, interoceptive exposure for social anxiety.<br><br>Positively related to use of in vivo self-exposure and exposure-based homework assignments for specific phobia. Unrelated to use of therapist-directed in vivo exposure, imaginal exposure, exposure and response prevention and interoceptive exposure for specific phobia.<br><br>Positively related to use of therapist-directed in vivo exposure, in vivo self-exposure, interoceptive exposure, and exposure-based homework assignments for panic disorder. Unrelated to use of imaginal exposure and exposure and response prevention for panic disorder.	Positively related	Anxiety disorders	Lifespan
	(Sars & van Minnen, 2015)	Clinician	Extent of exposure training in diagnostic skills for OCD.	Positively related to use of therapist-directed in vivo exposure, in vivo self-exposure, exposure and response prevention, and exposure-based homework assignments. Unrelated to use of imaginal	Positively related	OCD	Lifespan

TDF Domains	Study	Construct Focus	Constructs	Notes	Relationship to ET Use	Presentation	Developmental Subgroup
				exposure and interoceptive exposure.			
	(Sars & van Minnen, 2015)	Clinician	Extent of exposure training in empirical skills. Measured and analysed separately for each of the presentations (i.e., social anxiety, specific phobia, and panic disorder).	Positively related to use of therapist-directed in vivo exposure, in vivo self-exposure, and exposure-based homework assignments for social anxiety. Unrelated to use of imaginal exposure, exposure and response prevention, interoceptive exposure for social anxiety.<br><br>Positively related to use of therapist-directed in vivo exposure, in vivo self-exposure, and exposure-based homework assignments for specific phobia. Unrelated to use of imaginal exposure, exposure and response prevention and interoceptive exposure for specific phobia.<br><br>Positively related to use of therapist-directed in vivo exposure, in vivo self-exposure, interoceptive exposure, and exposure-based homework assignments for panic disorder. Unrelated to use of imaginal exposure and exposure and response prevention for panic disorder.	Positively related	Anxiety disorders	Lifespan
	(Sars & van Minnen, 2015)	Clinician	Extent of exposure training in empirical skills for OCD.	Positively related to use of therapist-directed in vivo exposure, in vivo self-exposure, exposure and	Positively related	OCD	Lifespan

TDF Domains	Study	Construct Focus	Constructs	Notes	Relationship to ET Use	Presentation	Developmental Subgroup
				response prevention, and exposure-based homework assignments. Unrelated to use of imaginal exposure and interoceptive exposure.			
	(van Minnen et al., 2010)	Clinician	Training in imaginal exposure.	-	Positively related	PTSD	Lifespan
	(Wade et al., 2020)	Clinician	Completion of a three-day interactive prolonged exposure therapy workshop, followed by at least 10 fortnightly one-hour group telephone consultations for six-months.	For all participants: Positively related to proportionate provision of prolonged exposure in vivo. Unrelated to proportional provision of prolonged exposure: rational and psychoeducation; imaginal; breathing retraining.<br><br>For completers (83.33% of sample): Positively related to proportionate provision of prolonged exposure in vivo. Unrelated to proportionate provision of prolonged exposure: rational and psychoeducation; imaginal; breathing retraining.	Positively related	PTSD	Lifespan
	(Whiteside, Biggs, Dammann, et al., 2022)	Clinician	Completion of training in an online and mobile-based application (Mayo Clinic Anxiety Coach) to conduct parent-coached exposure therapy. Involved 60 to 90-minute training phone call and provision of an iPod Touch, instructions, and a manual.	Unrelated to number of therapists reporting use of exposure therapy. Positively related to proportional use of exposure therapy.	Positively related	Anxiety-related presentations	Youth
Skills	(C. B. Becker et al., 2004)	Clinician	Experience treating PTSD.	-	Positively related	PTSD	Lifespan
	(S. Becker et al., 2018)	Clinician	Number of outpatient psychotherapy sessions completed in career.	-	Positively related	Anxiety disorders	Lifespan

TDF Domains	Study	Construct Focus	Constructs	Notes	Relationship to ET Use	Presentation	Developmental Subgroup
	(Becker-Haimes et al., 2017)	Clinician	Years of experience.	-	Unrelated	Anxiety-related presentations	Youth
	(de Jong et al., 2020)	Clinician	Years of experience.	-	Unrelated	Anxiety-related presentations	Youth
	(Finley et al., 2015)	Clinician	Years licensed as a provider.	-	Unrelated	PTSD	Adults
	(Harned et al., 2013)	Clinician	Years of experience working as a treatment provider since receiving degree.	-	Unrelated	Anxiety-related presentations	Lifespan
	(Harned et al., 2014)	Clinician	Online training on exposure therapy consisting of didactic instruction and simulated clinical scenarios.	-	Positively related	Anxiety-related presentations	Lifespan
	(Harned et al., 2011)	Clinician	Online training in exposure therapy involving instructions and client simulations combined with one to two motivational interviewing phone calls. Compared to online control training in dialectical behaviour therapy.	-	Unrelated	Anxiety-related presentations	Lifespan
	(Harned et al., 2021)	Clinician	Experience with exposure for PTSD.	-	Unrelated	PTSD	Lifespan
	(Harned et al., 2021)	Clinician	Four-day workshop (facilitated by developer and co-trainer) compared to two-day workshop (facilitated by developer) on dialectical behaviour therapy prolonged exposure. Workshop involved group activities, case-based learning, demonstrations, discussions, and role-playing.	-	Unrelated	PTSD	Lifespan

TDF Domains	Study	Construct Focus	Constructs	Notes	Relationship to ET Use	Presentation	Developmental Subgroup
	(Keleher et al., 2020)	Clinician	Years of experience in current role.	-	Unrelated	OCD	Youth
	(Keleher et al., 2020)	Clinician	Number of OCD cases treated.	-	Unrelated	OCD	Youth
	(Moses et al., 2021)	Clinician	Years of experience.	Unrelated to general, imaginal, and interoceptive exposure use.	Unrelated	Anxiety disorders	Lifespan
	(Moses et al., 2021)	Clinician	Years of experience.	Unrelated to general exposure and exposure and response prevention use.	Unrelated	OCD	Lifespan
	(Moses et al., 2021)	Clinician	Years of experience.	Unrelated to general and imaginal exposure use.	Unrelated	PTSD	Lifespan
	(Ruzek et al., 2017)	Clinician	Years of experience.	-	Unrelated	PTSD	Adults
	(Sars & van Minnen, 2015)	Clinician	Treatment experience with anxiety disorders in years.	Positively related to use of imaginal exposure for social anxiety. Unrelated to use of therapist-directed in vivo exposure, in vivo self-exposure, exposure and response prevention, interoceptive exposure, and exposure-based homework assignments for social anxiety.<br><br>Positively related to use of imaginal exposure and interoceptive exposure for specific phobia. Unrelated to use of therapist-directed in vivo exposure, in vivo self-exposure, exposure and response prevention, and exposure-based homework assignments for specific phobia.	Positively related	Anxiety disorders	Lifespan

TDF Domains	Study	Construct Focus	Constructs	Notes	Relationship to ET Use	Presentation	Developmental Subgroup
				Positively related to use of imaginal exposure for panic disorder. Unrelated to use of therapist-directed in vivo exposure, in vivo self-exposure, exposure and response prevention, interoceptive exposure, and exposure-based homework assignments for panic disorder.			
	(Sars & van Minnen, 2015)	Clinician	Treatment experience with anxiety disorders in years.	Positively related to use of imaginal exposure and exposure and response prevention. Unrelated to use of therapist-directed in vivo exposure, in vivo self-exposure, interoceptive exposure, and exposure-based homework assignments.	Positively related	OCD	Lifespan
	(Schumacher et al., 2018)	Clinician	Number of exposure sessions completed in training.	Unrelated for being a user (in 1 – 80% of cases) and a frequent user (in 81% or more of cases), relative to non-users.	Unrelated	PTSD	Lifespan
	(Sherrill et al., 2021)	Clinician	Completion of a six-month competency-based learning program for prolonged exposure. Involved live weekly training calls, completion of at least six sessions of prolonged exposure with two patients, and one weekly group meeting.	-	Positively related	PTSD	Lifespan
	(Whiteside, Biggs, Ollendick, et al., 2022)	Clinician	Expertise in using parent-coached exposure therapy for childhood anxiety disorders.	Unrelated to overall implementation of exposure (i.e., combining education, planning, and completing) and during in-session	Positively related	Anxiety-related presentations	Youth

TDF Domains	Study	Construct Focus	Constructs	Notes	Relationship to ET Use	Presentation	Developmental Subgroup
				exposure during treatment phase. Positively related to in vivo and imaginal exposure use during treatment phase. Positively related to percentage of sessions with any imaginal or in vivo exposure use.			
	(Whiteside, Deacon, et al., 2016)	Clinician	Years of experience.	-	Unrelated	Anxiety-related presentations	Youth
Social/Professional Role and Identity	(Chen et al., 2022)	Client	Aged 60 years or older (relative to 18 to 59 years).	-	Negatively related	Anxiety-related presentations	Adults
	(Chen et al., 2022)	Clinician	Highest clinical qualification in psychology.	-	Unrelated	Anxiety-related presentations	Adults
	(Cook, Dinnen, Thompson, et al., 2015)	Clinician	Professional discipline (psychiatrist, psychologist, or social worker).	-	Unrelated	PTSD	Adults
	(Cook et al., 2014)	Clinician	Occupation (psychiatrist, psychologist, social worker, or nurse).	-	Unrelated	PTSD	Adults
	(Cook, Thompson, et al., 2020)	Clinician	Occupation as a psychiatrist.	-	Unrelated	PTSD	Adults
	(Cook, Thompson, et al., 2020)	Clinician	Occupation as a psychologist.	-	Unrelated	PTSD	Adults
	(Cook, Thompson, et al., 2020)	Clinician	Occupation as a social worker.	-	Unrelated	PTSD	Adults

TDF Domains	Study	Construct Focus	Constructs	Notes	Relationship to ET Use	Presentation	Developmental Subgroup
	(de Jong et al., 2020)	Clinician	Age.	-	Negatively related	Anxiety-related presentations	Youth
	(de Jong et al., 2020)	Clinician	Endorsement of a pure cognitive-behavioural theoretical orientation (vs. combined or other orientation).	-	Positively related	Anxiety-related presentations	Youth
	(Finley et al., 2015)	Clinician	Endorsement of a primary cognitive-behavioural theoretical orientation.	-	Unrelated	PTSD	Adults
	(Garcia et al., 2020)	Clinician	Occupation as a psychologist (relative to a social worker).	-	Unrelated	PTSD	Adults
	(Garcia et al., 2020)	Clinician	Theoretical orientation as cognitive-behavioural.	-	Unrelated	PTSD	Adults
	(Harned et al., 2013)	Clinician	Higher academic degree achieved (i.e., bachelors level, masters level, doctoral level).	-	Positively related	Anxiety-related presentations	Lifespan
	(Harned et al., 2013)	Clinician	Endorsement of cognitive and/or behavioural theoretical orientation.	-	Unrelated	Anxiety-related presentations	Lifespan
	(Hertz et al., 2023)	Client	Age.	-	Negatively related	OCD	Adults
	(Hertz et al., 2023)	Client	Gender, dichotomised as male, female.	-	Unrelated	OCD	Adults
	(Hertz et al., 2023)	Client	Race, categorised as American Indian or Alaska Native, Asian, Black or African American, Native Hawaiian or other Pacific Islander, White, or other.	-	Unrelated	OCD	Adults
	(Hertz et al., 2023)	Client	Marital status, categorised as divorced, married, never married, separated, widow, or unknown.	-	Unrelated	OCD	Adults

TDF Domains	Study	Construct Focus	Constructs	Notes	Relationship to ET Use	Presentation	Developmental Subgroup
	(Hertz et al., 2023)	Client	Ethnicity, categorised as Hispanic or Latino, not Hispanic or Latino, or unknown.	-	Unrelated	OCD	Adults
	Hipol & Deacon, 2013)	Clinician	Self-identification as an anxiety specialist.	Unrelated for therapist-assisted in vivo exposure, self-directed in vivo exposure, and imaginal exposure. Positively related for interoceptive exposure.	Positively related	Anxiety-related presentations	Lifespan
	(Keleher et al., 2020)	Clinician	Professional background in clinical psychology relative to other backgrounds (e.g., nursing, psychiatry, psychotherapy).	Positively related overall and for taboo symptoms but unrelated for other symptom domains (i.e., contamination, aggression, hoarding).	Positively related	OCD	Youth
	(Keleher et al., 2020)	Clinician	Cognitive-behavioural therapeutic orientation relative to other orientations (e.g., family therapy/systemic, psychodynamic, dialectical behaviour therapy).	Unrelated overall and for most symptom domains (i.e., taboo, contamination, hoarding) but positively related for aggressive symptoms.	Unrelated	OCD	Youth
	(Klan et al., 2017)	Client	Self-reported family status (i.e., in a stable relationship).	-	Unrelated	Anxiety disorders	Adults
	(Maguen et al., 2019)	Client	Aged 30-49 (relative to <30).	-	Positively related	PTSD	Adults
	(Maguen et al., 2019)	Client	Aged 50+ (relative to <30).	-	Unrelated	PTSD	Adults
	(Maguen et al., 2019)	Client	Gender as female (relative to male).	-	Negatively related	PTSD	Adults
	(Maguen et al., 2019)	Client	Race as Black/African American (relative to White).	-	Positively related	PTSD	Adults
	(Maguen et al., 2019)	Client	Race as American Indian or Alaska Native (relative to White).	-	Unrelated	PTSD	Adults

TDF Domains	Study	Construct Focus	Constructs	Notes	Relationship to ET Use	Presentation	Developmental Subgroup
	(Maguen et al., 2019)	Client	Race as Asian (relative to White).	-	Unrelated	PTSD	Adults
	(Maguen et al., 2019)	Client	Race as Native Hawaiian or Other Pacific Islander (relative to White).	-	Unrelated	PTSD	Adults
	(Maguen et al., 2019)	Client	Race as multi-Race (relative to White).	-	Unrelated	PTSD	Adults
	(Maguen et al., 2019)	Client	Ethnicity as Not Hispanic or Latino (relative to Hispanic or Latino).	-	Negatively related	PTSD	Adults
	(Maguen et al., 2019)	Client	Ethnicity as Other (relative to Hispanic or Latino).	-	Negatively related	PTSD	Adults
	(Maguen et al., 2019)	Client	Service branch as Navy (relative to Air Force).	-	Negatively related	PTSD	Adults
	(Maguen et al., 2019)	Client	Service branch as Army (relative to Air Force).	-	Unrelated	PTSD	Adults
	(Maguen et al., 2019)	Client	Service branch as Coast Guard (relative to Air Force).	-	Unrelated	PTSD	Adults
	(Maguen et al., 2019)	Client	Service branch as Marine (relative to Air Force).	-	Unrelated	PTSD	Adults
	(Maguen et al., 2019)	Client	Service component as National Guard (relative to Active Duty).	-	Positively related	PTSD	Adults
	(Maguen et al., 2019)	Client	Service component as Reserve (relative to Active Duty).	-	Positively related	PTSD	Adults
	(Maguen et al., 2019)	Client	Rank as Officer (relative to Enlisted).	-	Positively related	PTSD	Adults
	(Maguen et al., 2019)	Client	Rank as Warrant (relative to Enlisted).	-	Unrelated	PTSD	Adults
	(Maguen et al., 2019)	Client	Having had multiple deployments (relative to a single deployment).	-	Positively related	PTSD	Adults

TDF Domains	Study	Construct Focus	Constructs	Notes	Relationship to ET Use	Presentation	Developmental Subgroup
	(Maguen et al., 2019)	Client	Having served in a combat zone.	-	Positively related	PTSD	Adults
	(Maguen et al., 2019)	Client	Relationship status as never married (relative to married).	-	Negatively related	PTSD	Adults
	(Maguen et al., 2019)	Client	Relationship status as divorced/separated/single (relative to married).	-	Negatively related	PTSD	Adults
	(Maguen et al., 2019)	Client	Relationship status as widow/widower/widowed (relative to married).	-	Unrelated	PTSD	Adults
	(Maguen et al., 2019)	Client	Education level.	-	Positively related	PTSD	Adults
	(Moritz et al., 2019)	Clinician	Professional background as a psychologist, relative to a physician.	-	Positively related	OCD	Lifespan
	(Moses et al., 2021)	Clinician	Cognitive-behavioural theoretical orientation, relative to other orientations.	Unrelated to general and interoceptive exposure use.	Unrelated	Anxiety disorders	Lifespan
	(Moses et al., 2021)	Clinician	Cognitive-behavioural theoretical orientation, relative to other orientations.	Unrelated to general exposure and exposure and response prevention use.	Unrelated	OCD	Lifespan
	(Moses et al., 2021)	Clinician	Cognitive-behavioural theoretical orientation, relative to other orientations.	Unrelated to general and imaginal exposure use.	Unrelated	PTSD	Lifespan
	(Moses et al., 2021)	Clinician	Registration status as a psychologist, categorised as provisionally registered, fully registered, or fully registered with endorsement.	Related to general, imaginal, and interoceptive exposure use. Direction unspecified.	Related	Anxiety disorders	Lifespan
	(Moses et al., 2021)	Clinician	Highest level of training, categorised as bachelor’s degree, master of psychology, clinical masters/doctorate, research PhD.	Related to general exposure use. Unrelated to imaginal and interoceptive exposure use. Direction unspecified.	Related	Anxiety disorders	Lifespan

TDF Domains	Study	Construct Focus	Constructs	Notes	Relationship to ET Use	Presentation	Developmental Subgroup
	(Moses et al., 2021)	Clinician	Registration status as a psychologist, categorised as provisionally registered, fully registered, or fully registered with endorsement.	Unrelated to general exposure and exposure and response prevention use.	Unrelated	OCD	Lifespan
	(Moses et al., 2021)	Clinician	Highest level of training, categorised as bachelor's degree, master of psychology, clinical masters/doctorate, research PhD.	Unrelated to general exposure use. Related to exposure and response prevention use. Direction unspecified.	Related	OCD	Lifespan
	(Moses et al., 2021)	Clinician	Registration status as a psychologist, categorised as provisionally registered, fully registered, or fully registered with endorsement.	Unrelated to general exposure use. Related to imaginal exposure use. Direction unspecified.	Related	PTSD	Lifespan
	(Moses et al., 2021)	Clinician	Highest level of training, categorised as bachelor's degree, master of psychology, clinical masters/doctorate, research PhD.	Related to general exposure use. Unrelated to imaginal exposure use. Direction unspecified.	Related	PTSD	Lifespan
	(Parker & Waller, 2019)	Clinician	Profession as a counselling psychologist relative to psychiatric nurse, CBT therapist, clinical psychologist, or other.	-	Positively related	Anxiety-related presentations	Lifespan
	(Reid et al., 2018)	Clinician	Gender (female, relative to male).	-	Unrelated	Anxiety disorders	Youth
	(Reid et al., 2018)	Clinician	Gender (female, relative to male).	-	Unrelated	OCD	Youth
	(Reid et al., 2018)	Clinician	Gender (female, relative to male).	-	Unrelated	PTSD	Youth
	(Reid et al., 2018)	Clinician	Self-reported specialisation in anxiety disorders.	-	Unrelated	Anxiety disorders	Youth
	(Reid et al., 2018)	Clinician	Self-reported specialisation in anxiety disorders.	-	Positively related	OCD	Youth
	(Reid et al., 2018)	Clinician	Self-reported specialisation in anxiety disorders.	-	Positively related	PTSD	Youth

TDF Domains	Study	Construct Focus	Constructs	Notes	Relationship to ET Use	Presentation	Developmental Subgroup
	(Reid et al., 2018)	Clinician	More than two years of graduate education, relative to one to two years.	-	Unrelated	Anxiety disorders	Youth
	(Reid et al., 2018)	Clinician	More than two years of graduate education, relative to one to two years.	-	Unrelated	OCD	Youth
	(Reid et al., 2018)	Clinician	More than two years of graduate education, relative to one to two years.	-	Unrelated	PTSD	Youth
	(Rosen et al., 2019)	Client	Age.	Negatively related, relative to initiation of other psychotherapies – but not cognitive processing therapy.	Negatively related	PTSD	Adults
	(Rosen et al., 2019)	Client	Gender (male, relative to female).	-	Positively related	PTSD	Adults
	(Rosen et al., 2019)	Client	Race as White.	Negatively related, relative to cognitive processing therapy - but not other psychotherapies.	Negatively related	PTSD	Adults
	(Rosen et al., 2019)	Client	Race as Black.	Positively related, relative to cognitive processing therapy - but not other psychotherapies.	Positively related	PTSD	Adults
	(Rosen et al., 2019)	Client	Race as Other (American Indian, Asian, Hawaiian, Multi-Race, or Unknown).	Positively related, relative to other psychotherapies - but not cognitive processing therapy.	Positively related	PTSD	Adults
	(Rosen et al., 2019)	Client	Ethnicity as Hispanic or Latino.	-	Unrelated	PTSD	Adults
	(Rosen et al., 2019)	Client	Marital status as never married, separated/divorced/widowed, or married.	-	Unrelated	PTSD	Adults
	(Rosen et al., 2017)	Clinician	Profession as a psychologist (relative to a psychiatrist/other).	-	Unrelated	PTSD	Adults
	(Rosen et al., 2017)	Clinician	Profession as a social worker (relative to a psychiatrist/other).	-	Unrelated	PTSD	Adults

TDF Domains	Study	Construct Focus	Constructs	Notes	Relationship to ET Use	Presentation	Developmental Subgroup
	(Rosen et al., 2017)	Clinician	Gender as male (relative to female).	-	Positively related	PTSD	Adults
	(Rowe & Kangas, 2020)	Clinician	Highest qualification achieved in psychology (i.e., clinical psychology, other postgraduate degree, or other qualifications).	Positively related to frequency of use. Unrelated to in-session duration of use.	Positively related	Anxiety-related presentations	Adults
	(Ruzek et al., 2017)	Clinician	Gender (male, relative to female).	-	Positively related	PTSD	Adults
	(Ruzek et al., 2017)	Clinician	Cognitive-behavioural therapy orientation (relative to no cognitive-behavioural therapy orientation).	-	Unrelated	PTSD	Adults
	(Ruzek et al., 2017)	Clinician	Occupation as a psychologist (relative to psychiatrist/other).	-	Positively related	PTSD	Adults
	(Ruzek et al., 2017)	Clinician	Occupation as a social worker (relative to psychiatrist/other).	-	Positively related	PTSD	Adults
	(Schumacher et al., 2018)	Clinician	Age.	For panic disorder: Unrelated for being a user (in 1 – 80% of cases), relative to non-user. Negatively related for being a frequent user (in 81% or more of cases), relative to non-user.<br><br>For phobia: Unrelated for being a user (in 1 – 80% of cases), relative to non-user. Negatively related for being a frequent user (in 81% or more of cases), relative to non-user.	Negatively related	Anxiety disorders	Lifespan
	(van Minnen et al., 2010)	Clinician	Sex as male, relative to female.	-	Positively related	PTSD	Lifespan
	(Whiteside, Deacon, et al., 2016)	Clinician	Theoretical orientation other than just CBT.	However, pure CBT orientation reported greater endorsed use than both CBT plus other orientations	Negatively related	Anxiety-related presentations	Youth

TDF Domains	Study	Construct Focus	Constructs	Notes	Relationship to ET Use	Presentation	Developmental Subgroup
				and other orientations. CBT plus other orientations reported greater endorsed use than other orientations.			
	(Whiteside, Deacon, et al., 2016)	Clinician	Self-identified expertise in childhood anxiety disorders.	-	Unrelated	Anxiety-related presentations	Youth
	(Whiteside, Deacon, et al., 2016)	Clinician	Profession with a PhD.	PhD psychologists reported greater endorsed and proportional use, relative to PsyD psychologists, social workers, masters degree licensed counsellors, marriage and family therapists. PsyD psychologists reported higher proportional use than marriage and family therapists.	Positively related	Anxiety-related presentations	Youth
Beliefs about Capabilities	(Harned et al., 2013)	Clinician	Confidence in ability to use exposure-therapy.	Confidence in ability to use exposure-therapy was unrelated overall. However, high confidence in ability to use exposure-therapy predicted higher use in those who completed online training (10-hour online course), motivational enhancement (brief five-minute video and simulated discussion with a virtual exposure therapy consultant), and an online learning community (eight one-hour learning community meetings facilitated by an experienced exposure therapy clinician). Compared to those with high confidence who only completed online training and	Positively related	Anxiety-related presentations	Lifespan

TDF Domains	Study	Construct Focus	Constructs	Notes	Relationship to ET Use	Presentation	Developmental Subgroup
				online training plus motivational enhancement.			
	(Harned et al., 2021)	Clinician	Self-efficacy with dialectical behaviour therapy.	-	Unrelated	PTSD	Lifespan
	(Harned et al., 2021)	Clinician	Self-efficacy with dialectical behaviour therapy prolonged exposure.	-	Positively related	PTSD	Lifespan
	(Moses et al., 2022)	Clinician	Confidence to use exposure therapy.	-	Positively related [a]	Anxiety-related presentations	Lifespan
	(Moses et al., 2022)	Clinician	Confidence to use exposure therapy.	Positively related to the use of exposure therapy for anxiety and interoceptive exposure for panic disorder.	Positively related	Anxiety disorders	Lifespan
	(Moses et al., 2022)	Clinician	Confidence to use exposure therapy.	-	Positively related	OCD	Lifespan
	(Moses et al., 2022)	Clinician	Confidence to use exposure therapy.	-	Positively related	PTSD	Lifespan
	(Parker & Waller, 2019)	Clinician	Self-esteem.	-	Unrelated	Anxiety-related presentations	Lifespan
	(Pittig et al., 2019)	Clinician	Self-reported competence conducting exposure.	-	Positively related	Anxiety-related presentations	Lifespan
	(Rosen et al., 2017)	Clinician	Self-efficacy to deliver prolonged exposure.	-	Unrelated	PTSD	Adults
	(Rosen et al., 2017)	Clinician	Perceived control over treatment plan.	-	Unrelated	PTSD	Adults
	(Rosen et al., 2017)	Clinician	Perceived control over own schedule.	-	Unrelated	PTSD	Adults

TDF Domains	Study	Construct Focus	Constructs	Notes	Relationship to ET Use	Presentation	Developmental Subgroup
	(Ruzek et al., 2017)	Clinician	Self-efficacy to deliver prolonged exposure.	Pre-training baseline levels and change throughout training unrelated.	Unrelated	PTSD	Adults
	(Ruzek et al., 2017)	Clinician	Self-efficacy to receive referrals for prolonged exposure.	Pre-training baseline levels and change throughout training positively related.	Positively related	PTSD	Adults
	(Ruzek et al., 2017)	Clinician	Control over treatment plan.	Pre-training baseline levels and change throughout training unrelated.	Unrelated	PTSD	Adults
	(Ruzek et al., 2017)	Clinician	Control over schedule.	Pre-training baseline levels unrelated. Change throughout training positively related.	Positively related	PTSD	Adults
	(Whiteside, Deacon, et al., 2016)	Clinician	Perceived resiliency of children with anxiety disorders.	-	Positively related	Anxiety-related presentations	Youth
Optimism	-	-	-	-	-	-	-
Beliefs about Consequences	(S. Becker et al., 2018)	Clinician	Negative beliefs about exposure.	Negatively related to overall endorsement of exposure therapy use and at least one performance of therapist-guided exposures (relative to none) for most recent client.<br><br>Unrelated to frequency of therapist-guided exposures in vivo, self-administered exposures in vivo, and interoceptive exposures for most recent client. Also unrelated to both at least one performance of exposure or self-exposure (relative to none) for most recent client.	Negatively related	Anxiety disorders	Lifespan

TDF Domains	Study	Construct Focus	Constructs	Notes	Relationship to ET Use	Presentation	Developmental Subgroup
	(S. Becker et al., 2018)	Clinician	Concerns about rejection of exposure therapy by patient.	-	Negatively related	Anxiety disorders	Lifespan
	(S. Becker et al., 2018)	Clinician	Concerns about side-effects of exposure therapy.	-	Negatively related	Anxiety disorders	Lifespan
	(Chen et al., 2022)	Clinician	Negative beliefs about exposure.	Negatively related to exposure use in older anxious adults (60+ years).	Negatively related	Anxiety-related presentations	Adults
	(Cook, Dinnen, Coyne, et al., 2015)	Clinician	Degree to which the innovation (prolonged exposure) is considered superior to existing practices.	-	Unrelated	PTSD	Adults
	(Cook, Dinnen, Coyne, et al., 2015)	Clinician	Degree to which the innovation's (prolonged exposure) results are observable to others.	-	Unrelated	PTSD	Adults
	(Cook, Dinnen, Thompson, et al., 2015)	Clinician	Positive view of prolonged exposure.	-	Positively related	PTSD	Adults
	(Cook, Dinnen, Thompson, et al., 2015)	Clinician	Perceived risk or uncertainty of outcome associated with prolonged exposure.	-	Unrelated	PTSD	Adults
	(Cook, Thompson, & Schnurr, 2015)	Clinician	Perceived positive characteristics of prolonged exposure (e.g., relative advantage, compatibility, complexity, trialability, observability, potential for reinvention, task issues, knowledge, technical support).	-	Positively related	PTSD	Adults
	(Cook, Thompson, &	Clinician	Perceived positive characteristics of cognitive processing therapy (e.g., relative advantage, compatibility, complexity,	-	Unrelated	PTSD	Adults

TDF Domains	Study	Construct Focus	Constructs	Notes	Relationship to ET Use	Presentation	Developmental Subgroup
	Schnurr, 2015)		trialability, observability, potential for reinvention, task issues, knowledge, technical support).				
	(Cook, Thompson, et al., 2020)	Clinician	Perceived positive view of prolonged exposure (e.g., relative advantage, compatibility, complexity, trialability, observability, potential for reinvention, task issues, knowledge, technical support).	-	Positively related	PTSD	Adults
	(Cook, Thompson, et al., 2020)	Clinician	Perceived risk associated with prolonged exposure use.	-	Unrelated	PTSD	Adults
	(Cook, Thompson, et al., 2020)	Clinician	Perceived positive view of cognitive processing therapy (e.g., relative advantage, compatibility, complexity, trialability, observability, potential for reinvention, task issues, knowledge, technical support).	-	Unrelated	PTSD	Adults
	(Cook, Thompson, et al., 2020)	Clinician	Perceived risk associated with cognitive processing therapy.	-	Unrelated	PTSD	Adults
	(Deacon et al., 2013)	Clinician	Less change in negative beliefs about exposure therapy following single-day (seven-hour) didactic workshop on nature and use of exposure-based anxiety treatment.	-	Positively related	Anxiety-related presentations	Lifespan
	(Deacon et al., 2013)	Clinician	Negative beliefs about exposure.	Negatively related to dichotomous exposure use in last year before single-day workshop, relative to non-use. Unrelated following single-day workshop.	Negatively related	Anxiety-related presentations	Lifespan
	(de Jong et al., 2020)	Clinician	Negative beliefs about exposure therapy.	-	Negatively related	Anxiety-related presentations	Youth

TDF Domains	Study	Construct Focus	Constructs	Notes	Relationship to ET Use	Presentation	Developmental Subgroup
	(de Jong et al., 2020)	Clinician	Belief that exposure therapy places children at a greater risk of harm.	-	Negatively related	Anxiety-related presentations	Youth
	(de Jong et al., 2020)	Clinician	Belief that most children refuse to participate in exposure therapy.	-	Negatively related	Anxiety-related presentations	Youth
	(de Jong et al., 2020)	Clinician	Perceived utility of cognitive strategies in the exposure-based treatment of anxiety in youth.	-	Positively related	Anxiety-related presentations	Youth
	(de Jong et al., 2020)	Clinician	Perceived utility of relaxation strategies in the exposure-based treatment of anxiety in youth.	-	Unrelated	Anxiety-related presentations	Youth
	(Finley et al., 2015)	Clinician	Perceived effectiveness of prolonged exposure.	-	Positively related	PTSD	Adults
	(Finley et al., 2015)	Clinician	Perceived effectiveness of cognitive processing therapy.	-	Negatively related	PTSD	Adults
	(Harned et al., 2013)	Clinician	Negative attitudes towards exposure therapy.	-	Unrelated	Anxiety-related presentations	Lifespan
	(Harned et al., 2021)	Clinician	Belief about likelihood that patient problems will be worsened during or after exposure therapy for PTSD.	-	Unrelated	PTSD	Lifespan
	(Harned et al., 2021)	Clinician	Perceived usefulness of dialectical behaviour therapy prolonged exposure.	-	Positively related	PTSD	Lifespan
	(Kannis-Dymand et al., 2022)	Clinician	Negative beliefs about exposure.	-	Negatively related	Anxiety disorders	Lifespan
	(Keleher et al., 2020)	Clinician	Negative beliefs about exposure.	-	Negatively related	OCD	Youth

TDF Domains	Study	Construct Focus	Constructs	Notes	Relationship to ET Use	Presentation	Developmental Subgroup
	(Klan et al., 2017)	Client	Treatment outcome expectancies.	-	Unrelated	Anxiety disorders	Adults
	(Klan et al., 2017)	Clinician	Treatment outcome expectancies.	Unrelated to ET use or amount in a sample of treatment completers. In the intent to treat sample was positively related to ET use, but unrelated to ET amount.	Positively related	Anxiety disorders	Adults
	(Meyer et al., 2020)	Clinician	Beliefs that safety behaviours are helpful for a range of outcomes during exposure.	-	Negatively related	Anxiety-related presentations	Lifespan
	(Meyer et al., 2020)	Clinician	Beliefs that safety behaviours protect the client during exposure.	-	Negatively related	Anxiety-related presentations	Lifespan
	(Meyer et al., 2020)	Clinician	Beliefs that safety behaviours uphold ethical and legal standards during exposure.	-	Unrelated	Anxiety-related presentations	Lifespan
	(Meyer et al., 2020)	Clinician	Beliefs that safety behaviours help manage the therapist's own distress during exposure.	-	Unrelated	Anxiety-related presentations	Lifespan
	(Meyer et al., 2020)	Clinician	Negative beliefs about exposure.	-	Positively related	Anxiety-related presentations	Lifespan
	(Moses et al., 2022)	Clinician	Negative beliefs about exposure.	-	Negatively related	Anxiety-related presentations	Lifespan
	(Moses et al., 2022)	Clinician	Negative beliefs about exposure.	Negatively related to the use of exposure therapy for anxiety and interoceptive exposure for panic disorder.	Negatively related	Anxiety disorders	Lifespan

TDF Domains	Study	Construct Focus	Constructs	Notes	Relationship to ET Use	Presentation	Developmental Subgroup
	(Moses et al., 2022)	Clinician	Negative beliefs about exposure.	-	Negatively related	OCD	Lifespan
	(Moses et al., 2022)	Clinician	Negative beliefs about exposure.	-	Negatively related	PTSD	Lifespan
	(Parker & Waller, 2019)	Clinician	Negative attitudes towards cognitive-behavioural therapy.	-	Unrelated	Anxiety-related presentations	Lifespan
	(Pittig et al., 2019)	Clinician	Negative beliefs about exposure.	-	Negatively related	Anxiety-related presentations	Lifespan
	(Reid et al., 2018)	Clinician	Negative beliefs about exposure.	-	Negatively related	Anxiety disorders	Youth
	(Reid et al., 2018)	Clinician	Negative beliefs about exposure.	-	Negatively related	OCD	Youth
	(Reid et al., 2018)	Clinician	Negative beliefs about exposure.	-	Unrelated	PTSD	Youth
	(Rosen et al., 2017)	Clinician	Belief that prolonged exposure is effective.	-	Positively related	PTSD	Adults
	(Rosen et al., 2017)	Clinician	Belief that prolonged exposure is too time-consuming.	-	Unrelated	PTSD	Adults
	(Rowe & Kangas, 2020)	Clinician	Negative beliefs about exposure.	Unrelated to frequency and in-session duration of use.	Unrelated	Anxiety-related presentations	Adults
	(Ruzek et al., 2017)	Clinician	Belief that prolonged exposure helps patients.	Pre-training baseline levels positively related. Change throughout training unrelated.	Positively related	PTSD	Adults
	(Ruzek et al., 2017)	Clinician	Belief that prolonged exposure is time consuming for clinician.	Pre-training baseline levels negatively related. Change throughout training unrelated.	Negatively related	PTSD	Adults

TDF Domains	Study	Construct Focus	Constructs	Notes	Relationship to ET Use	Presentation	Developmental Subgroup
	(Ruzek et al., 2017)	Clinician	Belief that prolonged exposure may harm patients.	Pre-training baseline levels negatively related. Change throughout training unrelated.	Negatively related	PTSD	Adults
	(Ruzek et al., 2017)	Clinician	Belief that prolonged exposure is emotionally demanding for clinician.	Pre-training baseline levels and change throughout training positively related.	Positively related	PTSD	Adults
	(Sars & van Minnen, 2015)	Clinician	Perceived treatment credibility of exposure therapy.	Positively related to use of therapist-directed in vivo exposure, in vivo self-exposure, and exposure-based homework assignments for social anxiety. Unrelated to use of imaginal exposure, exposure and response prevention, and interoceptive exposure for social anxiety.<br><br>Positively related to use of therapist-directed in vivo exposure, in vivo self-exposure, and exposure-based homework assignments for specific phobia. Unrelated to use of imaginal exposure, exposure and response prevention, and interoceptive exposure for specific phobia.<br><br>Positively related to use of therapist-directed in vivo exposure, in vivo self-exposure, interoceptive exposure, and exposure-based homework assignments for panic disorder. Unrelated to use of imaginal exposure and exposure	Positively related	Anxiety disorders	Lifespan

TDF Domains	Study	Construct Focus	Constructs	Notes	Relationship to ET Use	Presentation	Developmental Subgroup
				and response prevention for panic disorder.			
	(Sars & van Minnen, 2015)	Clinician	Perceived treatment credibility of exposure therapy.	Positively related to use of therapist-directed in vivo exposure, in vivo self-exposure, exposure and response prevention, and exposure-based homework assignments. Unrelated to use of imaginal exposure and interoceptive exposure.	Positively related	OCD	Lifespan
	(Schumacher et al., 2018)	Clinician	Negative beliefs about exposure.	For panic disorder: Unrelated for being a user (in 1 – 80% of cases), relative to non-user. Negatively related for being a frequent user (in 81% or more of cases), relative to non-user.<br><br>For phobia: Unrelated for being a user (in 1 – 80% of cases), relative to non-user. Negatively related for being a frequent user (in 81% or more of cases), relative to non-user.	Negatively related	Anxiety disorders	Lifespan
	(Schumacher et al., 2018)	Clinician	Negative beliefs about exposure.	Negatively related for being a user (in 1 – 80% of cases) and a frequent user (in 81% or more of cases), relative to non-users.	Negatively related	PTSD	Lifespan
	(van Minnen et al., 2010)	Clinician	Perceived barriers to use of imaginal exposure (i.e., concerns about side effects, symptom exacerbation, perceived patient desire, treatment utility, and risk of dropout).	-	Unrelated	PTSD	Lifespan

TDF Domains	Study	Construct Focus	Constructs	Notes	Relationship to ET Use	Presentation	Developmental Subgroup
	(van Minnen et al., 2010)	Clinician	Perceived treatment credibility of imaginal exposure.	-	Positively related	PTSD	Lifespan
	(Whiteside, Deacon, et al., 2016)	Clinician	Negative beliefs about exposure.	-	Negatively related	Anxiety-related presentations	Youth
	(Živčić-Bećirević et al., 2019)	Clinician	Negative beliefs about exposure.	-	Negatively related	Anxiety-related presentations	Lifespan
Reinforcement	-	-	-	-	-	-	-
Intentions	(Becker-Haimes et al., 2017)	Clinician	Openness to innovation.	-	Positively related	Anxiety-related presentations	Youth
	(Cook, Dinnen, Thompson, et al., 2015)	Clinician	Drive to adopt prolonged exposure.	-	Unrelated	PTSD	Adults
	(Cook, Dinnen, Thompson, et al., 2015)	Clinician	Openness to prolonged exposure.	-	Unrelated	PTSD	Adults
	(Harned et al., 2013)	Clinician	Online training (10-hour online course) plus motivational enhancement (brief five-minute video and simulated discussion with a virtual exposure therapy consultant). Compared to only online training and online training, motivational enhancement, and an online learning community (eight one-hour learning community meetings facilitated by an experienced exposure therapy clinician).	-	Unrelated	Anxiety-related presentations	Lifespan

TDF Domains	Study	Construct Focus	Constructs	Notes	Relationship to ET Use	Presentation	Developmental Subgroup
	(Harned et al., 2011)	Clinician	Online training in exposure therapy combined with one to two motivational interviewing phone calls. Compared to online training in exposure therapy alone and online control training in dialectical behaviour therapy.	-	Unrelated	Anxiety-related presentations	Lifespan
	(Sars & van Minnen, 2015)	Clinician	Willingness to apply exposure techniques.	Positively related to use of therapist-directed in vivo exposure, in vivo self-exposure, and exposure-based homework assignments for social anxiety. Unrelated to use of imaginal exposure, exposure and response prevention, and interoceptive exposure for social anxiety.<br><br>Positively related to use of therapist-directed in vivo exposure, in vivo self-exposure, and exposure-based homework assignments for specific phobia. Unrelated to use of imaginal exposure, exposure and response prevention, and interoceptive exposure for specific phobia.<br><br>Positively related to use of therapist-directed in vivo exposure, in vivo self-exposure, interoceptive exposure, and exposure-based homework assignments for panic disorder. Unrelated to use of imaginal exposure and exposure	Positively related	Anxiety disorders	Lifespan

TDF Domains	Study	Construct Focus	Constructs	Notes	Relationship to ET Use	Presentation	Developmental Subgroup
				and response prevention for panic disorder.			
	(Sars & van Minnen, 2015)	Clinician	Willingness to apply exposure techniques.	Positively related to use of therapist-directed in vivo exposure, exposure and response prevention, and exposure-based homework assignments. Unrelated to use of in vivo self-exposure, imaginal exposure, and interoceptive exposure.	Positively related	OCD	Lifespan
Goals	(Becker-Haimes et al., 2017)	Clinician	Use of relaxation.	-	Negatively related	Anxiety-related presentations	Youth
	(Cook, Thompson, et al., 2020)	Clinician	Use of cognitive processing therapy on an individual basis.	-	Unrelated	PTSD	Adults
	(Cook, Thompson, et al., 2020)	Clinician	Use of cognitive processing therapy on a group basis.	-	Negatively related	PTSD	Adults
	(Meyer et al., 2020)	Clinician	Frequency of using therapist safety behaviours.	-	Negatively related	Anxiety-related presentations	Lifespan
	(Meyer et al., 2020)	Clinician	Likelihood of excluding a client from exposure therapy due to client characteristics (e.g., comorbidity, insight, fragility).	-	Negatively related	Anxiety-related presentations	Lifespan

TDF Domains	Study	Construct Focus	Constructs	Notes	Relationship to ET Use	Presentation	Developmental Subgroup
	(Ruzek et al., 2017)	Clinician	Estimate of number of patients per week planning on treating with prolonged exposure.	-	Positively related	PTSD	Adults
	(Ruzek et al., 2017)	Clinician	Intent to use prolonged exposure following training and consultation.	-	Positively related	PTSD	Adults
	(Sars & van Minnen, 2015)	Clinician	Personal preference for exposure.	Positively related to use of therapist-directed in vivo exposure, in vivo self-exposure, and exposure-based homework assignments for social anxiety. Unrelated to use of imaginal exposure, exposure and response prevention, and interoceptive exposure for social anxiety.<br><br>Positively related to use of therapist-directed in vivo exposure, in vivo self-exposure, and exposure-based homework assignments for specific phobia. Unrelated to use of imaginal exposure, exposure and response prevention, and interoceptive exposure for specific phobia.<br><br>Positively related to use of therapist-directed in vivo exposure, in vivo self-exposure, interoceptive exposure, and exposure-based homework assignments for panic disorder. Unrelated to use of imaginal exposure and exposure	Positively related	Anxiety disorders	Lifespan

TDF Domains	Study	Construct Focus	Constructs	Notes	Relationship to ET Use	Presentation	Developmental Subgroup
				and response prevention for panic disorder.			
	(Sars & van Minnen, 2015)	Clinician	Personal preference for exposure.	Positively related to use of therapist-directed in vivo exposure, in vivo self-exposure, exposure and response prevention, and exposure-based homework assignments. Unrelated to use of imaginal exposure and interoceptive exposure.	Positively related	OCD	Lifespan
	(Vande Voort et al., 2010)	Clinician	Use of relaxation techniques.	-	Unrelated	Anxiety-related presentations	Youth
	(Vande Voort et al., 2010)	Clinician	Use of anxiety management techniques.	-	Negatively related	Anxiety-related presentations	Youth
	(Vande Voort et al., 2010)	Clinician	Use of behaviour management techniques.	-	Unrelated	Anxiety-related presentations	Youth
	(Whiteside, Biggs, Ollendick, et al., 2022)	Clinician	Allocation to a treatment condition delivering parent coached exposure therapy assisted by an application. Involved receiving 30- to 90-minutes of phone training around how to use the application. Relative to the treatment as usual condition, which received equivalent training in how to maintain their traditional treatment within the study framework.	Positively related to implementing all aspects of exposure (i.e., education, planning, and completing), including in orientation phase (exposure education and fear ladder building), and treatment phase (implementing overall, in vivo, and imaginal exposure). Positively related to percentage of sessions with some in-session or in vivo exposure.	Positively related	Anxiety-related presentations	Youth

TDF Domains	Study	Construct Focus	Constructs	Notes	Relationship to ET Use	Presentation	Developmental Subgroup
Memory, Attention, and Decision Processes	-	-	-	-	-	-	-
Environmental Context and Resources	(Becker-Haimes et al., 2020)	System	Organisational characteristics for supporting the use of exposure therapy.	-	Unrelated	Anxiety-related presentations	Lifespan
	(Becker-Haimes et al., 2017)	Clinician	Caseload.	-	Negatively related	Anxiety-related presentations	Youth
	(Becker-Haimes et al., 2017)	System	Organisational climate of functionality (i.e., climates in which clinicians can get their job done effectively and have a well-defined understanding of how they fit into the organisation).	-	Unrelated	Anxiety-related presentations	Youth
	(Becker-Haimes et al., 2017)	System	Organisational culture of proficiency (i.e., cultures in which clinicians prioritize the well-being of clients and place emphasis on clinicians being up to date with the latest evidence).	-	Unrelated	Anxiety-related presentations	Youth
	(Becker-Haimes et al., 2017)	System	Organisational culture of rigidity (i.e., cultures in which clinicians are expected to show little interest in new ways to provide services and suppress efforts for change).	-	Unrelated	Anxiety-related presentations	Youth
	(Becker-Haimes et al., 2017)	System	Number of therapists in organisation.	-	Unrelated	Anxiety-related presentations	Youth
	(Cook, Dinnen, Coyne, et al., 2015)	Clinician	Consistency of the innovation (prolonged exposure) with existing values, experiences, and needs of adopter and system.	-	Unrelated	PTSD	Adults

TDF Domains	Study	Construct Focus	Constructs	Notes	Relationship to ET Use	Presentation	Developmental Subgroup
	(Cook, Dinnen, Coyne, et al., 2015)	System	Availability of means needed to implement the innovation (prolonged exposure; e.g., funding, time, access, administrative support).	-	Unrelated	PTSD	Adults
	(Cook, Dinnen, Thompson, et al., 2015)	System	Number of beds in residential treatment program.	-	Unrelated	PTSD	Adults
	(Cook, Dinnen, Thompson, et al., 2015)	System	Average length of patient stay in residential treatment program.	-	Unrelated	PTSD	Adults
	(Cook, Dinnen, Thompson, et al., 2015)	System	Structural context of risk-taking climate, managerial relations, goals and priorities, leadership and vision, high quality data capture, and enabling of knowledge sharing.	-	Unrelated	PTSD	Adults
	(Cook, Dinnen, Thompson, et al., 2015)	System	Supportive context including dedicated time and resources, and incentives and mandates.	-	Positively related	PTSD	Adults
	(Cook, Simiola, et al., 2020)	System	Changes to core members of team (i.e., staff turnover).	-	Negatively related	PTSD	Adults
	(Cook, Simiola, et al., 2020)	System	Program director encourages staff to implement evidence-based practices (i.e., cognitive processing therapy or prolonged exposure) and provides time and resources for adoption.	-	Unrelated	PTSD	Adults
	(Cook, Thompson, et al., 2020)	System	Number of beds in residential treatment program.	-	Unrelated	PTSD	Adults

TDF Domains	Study	Construct Focus	Constructs	Notes	Relationship to ET Use	Presentation	Developmental Subgroup
	(Cook, Thompson, et al., 2020)	System	Average length of stay in residential treatment program.	-	Unrelated	PTSD	Adults
	(de Jong et al., 2020)	Clinician	Caseload.	-	Unrelated	Anxiety-related presentations	Youth
	(Finley et al., 2015)	Clinician	Perception of clinical workload as too much.	-	Unrelated	PTSD	Adults
	(Finley et al., 2015)	System	Perception that clinic is understaffed.	-	Unrelated	PTSD	Adults
	(Harned et al., 2013)	System	The extent to which common organizational barriers interfered with ability to learn and apply exposure therapy.	-	Unrelated	Anxiety-related presentations	Lifespan
	(Harned et al., 2013)	Clinician	Primary work setting (i.e., private practice, outpatient, or non-outpatient).	-	Unrelated	Anxiety-related presentations	Lifespan
	(Harned et al., 2013)	Clinician	Perceived interference of high client caseload on ability to learn and apply exposure therapy.	-	Unrelated	Anxiety-related presentations	Lifespan
	(Harned et al., 2013)	Clinician	Perceived interference of lack of anxiety disorder clients on ability to learn and apply exposure therapy.	-	Unrelated	Anxiety-related presentations	Lifespan
	(Harned et al., 2013)	Clinician	Perceived interference of client's resistance to change on ability to learn and apply exposure therapy.	-	Unrelated	Anxiety-related presentations	Lifespan
	(Klan et al., 2017)	Client	Axis I comorbidity.	-	Unrelated	Anxiety disorders	Adults
	(Maguen et al., 2019)	Client	Residence as rural (relative to urban).	-	Negatively related	PTSD	Adults

TDF Domains	Study	Construct Focus	Constructs	Notes	Relationship to ET Use	Presentation	Developmental Subgroup
	(Maguen et al., 2019)	Client	Residence as highly rural (relative to urban).	-	Unrelated	PTSD	Adults
	(Maguen et al., 2019)	Client	Having had military sexual trauma.	-	Positively related	PTSD	Adults
	(Maguen et al., 2019)	Client	Having recently smoked.	-	Negatively related	PTSD	Adults
	(Maguen et al., 2019)	Client	On medication for PTSD.	-	Positively related	PTSD	Adults
	(Maguen et al., 2019)	Client	Number of comorbidities.	-	Positively related	PTSD	Adults
	(Maguen et al., 2019)	Client	Having a comorbidity of schizophrenia.	-	Negatively related	PTSD	Adults
	(Maguen et al., 2019)	Client	Having a comorbidity of bipolar.	-	Negatively related	PTSD	Adults
	(Maguen et al., 2019)	Client	Having a comorbidity of psychosis.	-	Unrelated	PTSD	Adults
	(Maguen et al., 2019)	Client	Having a comorbidity of pain.	-	Positively related	PTSD	Adults
	(Maguen et al., 2019)	Client	Having a comorbidity of traumatic brain injury.	-	Positively related	PTSD	Adults
	(Maguen et al., 2019)	Client	Having a comorbidity of depression.	-	Positively related	PTSD	Adults
	(Maguen et al., 2019)	Client	Having a comorbidity of alcohol abuse.	-	Positively related	PTSD	Adults
	(Maguen et al., 2019)	Client	Having a comorbidity of alcohol dependence.	-	Positively related	PTSD	Adults
	(Maguen et al., 2019)	Client	Having a comorbidity of drug abuse.	-	Unrelated	PTSD	Adults

TDF Domains	Study	Construct Focus	Constructs	Notes	Relationship to ET Use	Presentation	Developmental Subgroup
	(Maguen et al., 2019)	Client	Having a comorbidity of drug dependence.	-	Unrelated	PTSD	Adults
	(Maguen et al., 2019)	Client	Having a comorbidity of suicide ideation or attempt.	-	Positively related	PTSD	Adults
	(Maguen et al., 2019)	Client	Year of first mental health visit to the Veterans Health Administration as 2006 to 2017 (relative to 2001-2005).	-	Positively related	PTSD	Adults
	(Maguen et al., 2019)	Client	Number of non-evidence-based therapy sessions received by client before first session of prolonged exposure.	Positively related when both including and excluding clients who received no sessions of non-evidence-based therapy.	Positively related	PTSD	Adults
	(Maguen et al., 2019)	Client	Presentation is service connected.	-	Positively related	PTSD	Adults
	(Moses et al., 2022)	Client	Presentation categorised as OCD (relative to anxiety and PTSD) or anxiety (relative to PTSD).	Exposure and response prevention greatest for OCD, then anxiety, and then PTSD. No difference on self-directed in vivo, therapist-assisted in vivo, or interoceptive exposure.	Positively related	Anxiety-related presentations	Lifespan
	(Moses et al., 2022)	Client	Presentation categorised as anxiety, OCD, or PTSD.	No difference on self-directed in vivo, therapist-assisted in vivo, or interoceptive exposure.	Unrelated	Anxiety-related presentations	Lifespan
	(Moses et al., 2021)	Client	Presentation categorised as PTSD (relative to anxiety and OCD).	Imaginal exposure greater for PTSD than anxiety and OCD.	Positively related	Anxiety-related presentations	Lifespan
	(Moses et al., 2021)	Clinician	Frequency of working with clients with anxiety, OCD, and PTSD.	Unrelated to general exposure and exposure and response prevention use.	Unrelated	OCD	Lifespan
	(Moses et al., 2021)	Clinician	Frequency of working with clients with anxiety, OCD, and PTSD.	Positively related to general exposure use. Unrelated to imaginal exposure use.	Positively related	PTSD	Lifespan

TDF Domains	Study	Construct Focus	Constructs	Notes	Relationship to ET Use	Presentation	Developmental Subgroup
	(Moses et al., 2021)	Clinician	Frequency of working with clients with anxiety, OCD, and PTSD.	Positively related to general, imaginal, and interoceptive exposure use.	Positively related	Anxiety disorders	Lifespan
	(Parker & Waller, 2019)	Clinician	Hours worked per week.	-	Unrelated	Anxiety-related presentations	Lifespan
	(Parker & Waller, 2019)	Clinician	Average number of sessions when treating a client with an anxiety disorder.	-	Unrelated	Anxiety-related presentations	Lifespan
	(Parker & Waller, 2019)	Clinician	Hours spent with clients with anxiety disorders per week.	-	Unrelated	Anxiety-related presentations	Lifespan
	(Pittig et al., 2019)	Clinician	Perceived practicability barriers for conducting exposure.	-	Negatively related	Anxiety-related presentations	Lifespan
	(Rosen et al., 2019)	Client	Distance from home to nearest Veterans Affairs clinic.	Negatively related, relative to other psychotherapies – but unrelated relative to cognitive processing therapy.	Negatively related	PTSD	Adults
	(Rosen et al., 2019)	Client	Number of co-occurring psychiatric diagnoses.	Negatively related, relative to other psychotherapies – but unrelated relative to cognitive processing therapy.	Negatively related	PTSD	Adults
	(Rosen et al., 2019)	Client	Residence categorised as urban (relative to rural).	Positively related, relative to cognitive processing therapy – but negatively related, relative to other psychotherapies.	Related	PTSD	Adults
	(Rosen et al., 2019)	Client	Hospitalisation in last year for psychiatric treatment.	-	Unrelated	PTSD	Adults

TDF Domains	Study	Construct Focus	Constructs	Notes	Relationship to ET Use	Presentation	Developmental Subgroup
	(Rosen et al., 2019)	Client	Hospitalisation in last year for substance use treatment.	-	Unrelated	PTSD	Adults
	(Rosen et al., 2019)	Client	Having Veteran Affairs service-connected disability for PTSD.	Negatively related, relative to other psychotherapies – but unrelated relative to cognitive processing therapy.	Negatively related	PTSD	Adults
	(Rosen et al., 2017)	Clinician	Number of PTSD patients per week.	-	Positively related	PTSD	Adults
	(Rosen et al., 2017)	Clinician	Patient panel size.	-	Unrelated	PTSD	Adults
	(Rosen et al., 2017)	Clinician	Proportion of PTSD patients seen weekly.	-	Positively related	PTSD	Adults
	(Rosen et al., 2017)	System	Flow of interested patients.	-	Positively related	PTSD	Adults
	(Rosen et al., 2017)	Clinician	More common delivery of group (relative to individual) therapy.	-	Negatively related	PTSD	Adults
	(Rosen et al., 2017)	System	Clinic type as a PTSD outpatient clinic (relative to clinics that are not PTSD speciality programs).	-	Positively related	PTSD	Adults
	(Rosen et al., 2017)	System	Clinic type as a PTSD residential or day treatment (relative to clinics that are not PTSD speciality programs).	-	Unrelated	PTSD	Adults
	(Ruzek et al., 2017)	Clinician	Total patient caseload.	-	Positively related	PTSD	Adults
	(Ruzek et al., 2017)	Clinician	PTSD clients per week.	-	Positively related	PTSD	Adults
	(Ruzek et al., 2017)	System	Working in a PTSD speciality clinic.	-	Positively related	PTSD	Adults

TDF Domains	Study	Construct Focus	Constructs	Notes	Relationship to ET Use	Presentation	Developmental Subgroup
	(Sars & van Minnen, 2015)	Clinician	Proportion of caseload with an anxiety disorder.	Positively related to use of therapist-directed in vivo exposure and in vivo self-exposure for social anxiety. Unrelated to use of imaginal exposure, exposure and response prevention, interoceptive exposure, and exposure-based homework assignments for social anxiety.<br><br>Positively related to use of therapist-directed in vivo exposure and in vivo self-exposure for specific phobia. Unrelated to use of imaginal exposure, exposure and response prevention, interoceptive exposure, and exposure-based homework assignments for specific phobia.<br><br>Unrelated to use of therapist-directed in vivo exposure, in vivo self-exposure, imaginal exposure, exposure and response prevention, interoceptive exposure, and exposure-based homework assignments for panic disorder.	Positively related	Anxiety disorders	Lifespan
	(Sars & van Minnen, 2015)	Clinician	Proportion of caseload with an anxiety disorder.	Positively related to use of therapist-directed in vivo exposure. Unrelated to use of in vivo self-exposure, imaginal exposure, exposure and response prevention, interoceptive exposure, and	Positively related	OCD	Lifespan

TDF Domains	Study	Construct Focus	Constructs	Notes	Relationship to ET Use	Presentation	Developmental Subgroup
				exposure-based homework assignments.			
	(Sars & van Minnen, 2015)	System	Perceived practical limitations to use of exposure in workplace.	Negatively related to use of therapist-directed in vivo exposure for social anxiety. Unrelated to use of in vivo self-exposure, imaginal exposure, exposure and response prevention, interoceptive exposure, and exposure-based homework assignments for social anxiety.<br><br>Negatively related to use of therapist-directed in vivo exposure for specific phobia. Unrelated to use of in vivo self-exposure, imaginal exposure, exposure and response prevention, interoceptive exposure, and exposure-based homework assignments for specific phobia.<br><br>Negatively related to use of therapist-directed in vivo exposure for panic disorder. Unrelated to use of in vivo self-exposure, imaginal exposure, exposure and response prevention, interoceptive exposure, and exposure-based homework assignments for panic disorder.	Negatively related	Anxiety disorders	Lifespan
	(Sars & van Minnen, 2015)	System	Perceived practical limitations to use of exposure in workplace.	Negatively related to use of therapist-directed in vivo exposure. Unrelated to use of in vivo self-exposure, imaginal exposure,	Negatively related	OCD	Lifespan

TDF Domains	Study	Construct Focus	Constructs	Notes	Relationship to ET Use	Presentation	Developmental Subgroup
				exposure and response prevention, interoceptive exposure, and exposure-based homework assignments.			
	(Vande Voort et al., 2010)	Client	Pre- to post-treatment improved change in impact of anxiety on child and parent's daily functioning.	-	Positively related	Anxiety-related presentations	Youth
	(Vande Voort et al., 2010)	Client	Initial impact of anxiety on child and parent's daily functioning.	-	Unrelated	Anxiety-related presentations	Youth
	(Whiteside, Sattler, et al., 2016)	Client	Anxiety diagnosis as the primary presentation (relative to a comorbid presentation).	-	Positively related	Anxiety-related presentations	Youth
	(Whiteside, Sattler, et al., 2016)	Client	Anxiety diagnosis, as OCD, social phobia, or specific phobia (compared to not present for each).	Diagnoses of general anxiety disorder or overanxious disorder, panic disorder with or without agoraphobia, separation anxiety disorder, or PTSD were unrelated.	Positively related	Anxiety-related presentations	Youth
	(Whiteside, Sattler, et al., 2016)	System	Clinic specialisation as a childhood anxiety disorders speciality clinic (compared to both primary care clinic and general mental health clinic).	-	Positively related	Anxiety-related presentations	Youth
	(Whiteside et al., 2023)	System	Working in an integrated behavioural health setting (usually as part of a multi-disciplinary team with greater focus on continual training and efficiency), relative to a traditional setting (i.e., primary care clinics).	In relation to self-reported exposure use.	Positively related	Anxiety disorders	Youth
	(Whiteside et al., 2023)	System	Working in an integrated behavioural health setting (usually as part of a multi-disciplinary team with greater focus on continual training	In relation to coded exposure use via session audio recordings.	Unrelated	Anxiety disorders	Youth

TDF Domains	Study	Construct Focus	Constructs	Notes	Relationship to ET Use	Presentation	Developmental Subgroup
			and efficiency), relative to a traditional setting (i.e., primary care clinics).				
Social Influences	(Becker-Haimes et al., 2017)	System	Organisational implementation culture.	-	Positively related	Anxiety-related presentations	Youth
	(Cook, Dinnen, Coyne, et al., 2015)	System	Style of leadership with identified and articulated trajectory toward implementation of the innovation (prolonged exposure).	-	Positively related	PTSD	Adults
	(Cook, Dinnen, Coyne, et al., 2015)	Clinician	The structure and quality of the therapist's social network, both formal and informal.	-	Unrelated	PTSD	Adults
	(Cook, Dinnen, Thompson, et al., 2015)	Clinician	Quality and influence of social network relating to prolonged exposure.	-	Unrelated	PTSD	Adults
	(Cook, Simiola, et al., 2020)	System	Program prioritising another treatment over cognitive processing therapy.	-	Unrelated	PTSD	Adults
	(Cook, Simiola, et al., 2020)	System	Program prioritising cognitive processing therapy over prolonged exposure.	-	Unrelated	PTSD	Adults
	(Finley et al., 2015)	System	Perception that organizational politics negatively impacts work.	-	Unrelated	PTSD	Adults
	(Finley et al., 2015)	System	Perception of fair treatment by superiors.	-	Unrelated	PTSD	Adults
	(Foa et al., 2020)	System	Addition of one-hour weekly telephone consultation with prolonged exposure expert over the course of two client cases, relative	-	Positively related	PTSD	Adults

TDF Domains	Study	Construct Focus	Constructs	Notes	Relationship to ET Use	Presentation	Developmental Subgroup
			to just a four-day prolonged exposure workshop.				
	(Harned et al., 2013)	System	Online training (10-hour online course), motivational enhancement (brief five-minute video and simulated discussion with a virtual exposure therapy consultant), and an online learning community (eight one-hour learning community meetings facilitated by an experienced exposure therapy clinician). Compared to only online training and online training plus motivational enhancement.	-	Unrelated	Anxiety-related presentations	Lifespan
	(Harned et al., 2014)	System	Addition of a learning community (eight expert-facilitated one-hour community video-conference meetings over 12 weeks) on top of online training on exposure therapy consisting of didactic instruction and simulated clinical scenarios and a motivational enhancement (a video and simulated online conversation with an expert) aimed at addressing attitudinal barriers to exposure therapy.	-	Unrelated	Anxiety-related presentations	Lifespan
	(Keleher et al., 2020)	System	Amount of supervision received per month.	-	Unrelated	OCD	Youth
	(Klan et al., 2017)	Clinician / Client	Therapeutic process including alliance rated by both therapist and client.	-	Unrelated	Anxiety disorders	Adults
	(Moses et al., 2022)	System	Supervisor's recommendation to use exposure therapy, relative to no.	-	Unrelated	Anxiety-related presentations	Lifespan
	(Moses et al., 2022)	System	Supervisor's recommendation to use exposure therapy, relative to no.	Unrelated to the use of exposure therapy for anxiety and	Unrelated	Anxiety disorders	Lifespan

TDF Domains	Study	Construct Focus	Constructs	Notes	Relationship to ET Use	Presentation	Developmental Subgroup
				interoceptive exposure for panic disorder.			
	(Moses et al., 2022)	System	Supervisor's recommendation to use exposure therapy, relative to no.	-	Unrelated	OCD	Lifespan
	(Moses et al., 2022)	System	Supervisor's recommendation to use exposure therapy, relative to no.	-	Unrelated	PTSD	Lifespan
	(Parker & Waller, 2019)	System	Hours of supervision given or received per month.	-	Unrelated	Anxiety-related presentations	Lifespan
	(Rowe & Kangas, 2020)	Clinician	Secure attachment style.	Unrelated to frequency and in-session duration of use.	Unrelated	Anxiety-related presentations	Adults
	(Rowe & Kangas, 2020)	Clinician	Preoccupied (anxious) attachment style.	Unrelated to frequency and in-session duration of use.	Unrelated	Anxiety-related presentations	Adults
	(Rowe & Kangas, 2020)	Clinician	Dismissing (avoidant) attachment style.	Unrelated to frequency and in-session duration of use.	Unrelated	Anxiety-related presentations	Adults
	(Rowe & Kangas, 2020)	Clinician	Fearful attachment style.	Unrelated to frequency and in-session duration of use.	Unrelated	Anxiety-related presentations	Adults
Emotion	(Becker-Haimes et al., 2017)	Clinician	Perceived intuitive appeal of evidence-based practice.	-	Unrelated	Anxiety-related presentations	Youth
	(de Jong et al., 2020)	Clinician	Depressive symptoms.	-	Unrelated	Anxiety-related presentations	Youth
	(de Jong et al., 2020)	Clinician	Anxiety symptoms.	-	Unrelated	Anxiety-related presentations	Youth

TDF Domains	Study	Construct Focus	Constructs	Notes	Relationship to ET Use	Presentation	Developmental Subgroup
	(de Jong et al., 2020)	Clinician	Stress symptoms.	-	Unrelated	Anxiety-related presentations	Youth
	(Harned et al., 2013)	Clinician	Anxiety sensitivity.	-	Unrelated	Anxiety-related presentations	Lifespan
	(Klan et al., 2017)	Client	Self-reported cognitions around agoraphobia.	-	Unrelated	Anxiety disorders	Adults
	(Klan et al., 2017)	Client	Self-reported bodily sensations involved in panic disorder/agoraphobia.	-	Unrelated	Anxiety disorders	Adults
	(Klan et al., 2017)	Client	Self-reported agoraphobia avoidance when alone and with another person.	-	Unrelated	Anxiety disorders	Adults
	(Klan et al., 2017)	Client	Self-reported anxiety.	-	Unrelated	Anxiety disorders	Adults
	(Klan et al., 2017)	Client	Self-reported phobic anxiety.	Unrelated overall. Positively related in a subsample of treatment completers.	Positively related	Anxiety disorders	Adults
	(Klan et al., 2017)	Client	Self-reported depressive symptoms.	-	Unrelated	Anxiety disorders	Adults
	(Klan et al., 2017)	Client	Self-reported psychological distress.	-	Unrelated	Anxiety disorders	Adults
	(Klan et al., 2017)	Client	Perceived client psychosocial stress rated by therapist.	-	Unrelated	Anxiety disorders	Adults
	(Parker & Waller, 2019)	Clinician	Prospective anxiety.	-	Unrelated	Anxiety-related presentations	Lifespan
	(Parker & Waller, 2019)	Clinician	Inhibitory anxiety.	-	Negatively related	Anxiety-related presentations	Lifespan

TDF Domains	Study	Construct Focus	Constructs	Notes	Relationship to ET Use	Presentation	Developmental Subgroup
	(Pittig et al., 2019)	Clinician	Distress around exposure.	-	Negatively related	Anxiety-related presentations	Lifespan
	(Moses et al., 2022)	Clinician	Worry or generalised anxiety.	-	Unrelated	Anxiety-related presentations	Lifespan
	(Moses et al., 2022)	Clinician	Worry or generalised anxiety.	Unrelated to the use of exposure therapy for anxiety and interoceptive exposure for panic disorder.	Unrelated	Anxiety disorders	Lifespan
	(Moses et al., 2022)	Clinician	Worry or generalised anxiety.	-	Unrelated	OCD	Lifespan
	(Moses et al., 2022)	Clinician	Worry or generalised anxiety.	-	Unrelated	PTSD	Lifespan
	(Reid et al., 2018)	Clinician	Disgust sensitivity.	-	Unrelated	Anxiety disorders	Youth
	(Reid et al., 2018)	Clinician	Disgust sensitivity.	-	Unrelated	OCD	Youth
	(Reid et al., 2018)	Clinician	Disgust sensitivity.	-	Unrelated	PTSD	Youth
	(Reid et al., 2018)	Clinician	Anxiety sensitivity.	-	Unrelated	Anxiety disorders	Youth
	(Reid et al., 2018)	Clinician	Anxiety sensitivity.	-	Unrelated	OCD	Youth
	(Reid et al., 2018)	Clinician	Anxiety sensitivity.	-	Unrelated	PTSD	Youth
	(Sars & van Minnen, 2015)	Clinician	Fearful avoidance of exposure.	Unrelated to use of therapist-directed in vivo exposure, in vivo self-exposure, imaginal exposure,	Negatively related	Anxiety disorders	Lifespan

TDF Domains	Study	Construct Focus	Constructs	Notes	Relationship to ET Use	Presentation	Developmental Subgroup
				exposure and response prevention, interoceptive exposure, and exposure-based homework assignments for social anxiety.<br><br>Negatively related to use of in vivo self-exposure and exposure-based homework assignments for specific phobia. Unrelated to use of therapist-directed in vivo exposure, imaginal exposure, exposure and response prevention, and interoceptive exposure for specific phobia.<br><br>Negatively related to use of therapist-directed in vivo exposure for panic disorder. Unrelated to use of in vivo self-exposure, imaginal exposure, exposure and response prevention, interoceptive exposure, and exposure-based homework assignments for panic disorder.			
	(Sars & van Minnen, 2015)	Clinician	Fearful avoidance of exposure.	Negatively related to use of therapist-directed in vivo exposure. Unrelated to use of in vivo self-exposure, imaginal exposure, exposure and response prevention, interoceptive exposure, and exposure-based homework assignments.	Negatively related	OCD	Lifespan

TDF Domains	Study	Construct Focus	Constructs	Notes	Relationship to ET Use	Presentation	Developmental Subgroup
	(Vande Voort et al., 2010)	Client	Pre- to post-treatment change in anxiety.	-	Unrelated	Anxiety-related presentations	Youth
	(Vande Voort et al., 2010)	Client	Initial anxiety symptoms.	-	Unrelated	Anxiety-related presentations	Youth
Behavioural Regulation	(Becker-Haimes et al., 2017)	Clinician	Perceived divergence between evidence-based and current practices.	-	Unrelated	Anxiety-related presentations	Youth
	(Becker-Haimes et al., 2017)	Clinician	Willingness to use evidence-based practice if required by organisation, supervisor, or state.	-	Negatively related	Anxiety-related presentations	Youth
Unclassified	(Cook et al., 2014)	Clinician	Adaptations made to prolonged exposure ranging from tailoring to abandoning principles of the intervention.	-	Unrelated	PTSD	Adults
	(Cook, Thompson, et al., 2020)	Clinician	Positive attitudes towards evidence-based practices, including appeal, requirements, openness, and divergence with clinical utility.	-	Unrelated	PTSD	Adults
	(Reid et al., 2017)	Clinician	Number of perceived potential barriers to exposure therapy implementation (e.g., session length, concern about client reaction, lack of resources, lack of training, ethical considerations).	-	Negatively related	OCD	Youth
	(Reid et al., 2017)	Clinician	Number of perceived potential barriers to exposure therapy implementation (e.g., session length, concern about client reaction, lack of resources, lack of training, ethical considerations).	-	Negatively related	Anxiety disorders	Youth
	(Reid et al., 2017)	Clinician	Number of perceived potential barriers to exposure therapy implementation (e.g.,	-	Unrelated	PTSD	Youth

TDF Domains	Study	Construct Focus	Constructs	Notes	Relationship to ET Use	Presentation	Developmental Subgroup
			session length, concern about client reaction, lack of resources, lack of training, ethical considerations).				

Note.
[a] Discrepancies existed between the reported direction of the statistical result and its interpretation, as such the predominant interpretation was considered accurate.
